# Unscrambling phylogenetic effects and ecological determinants of chromosome number in major angiosperm clades

**DOI:** 10.1038/s41598-018-32515-x

**Published:** 2018-09-24

**Authors:** Angelino Carta, Gianni Bedini, Lorenzo Peruzzi

**Affiliations:** Department of Biology, Unit of Botany, University of Pisa, via Derna 1, 56126 Pisa, Italy

## Abstract

As variations in the chromosome number are recognized to be of evolutionary interest but are also widely debated in the literature, we aimed to quantitatively test for possible relationships among the chromosome number, plant traits, and environmental factors. In particular, the chromosome number and drivers of its variation were examined in 801 Italian endemic vascular plants, for a total of 1364 accessions. We estimated phylogenetic inertia and adaptation in chromosome number - based on an Ornstein-Uhlenbeck process - and related chromosome numbers with other plant traits and environmental variables. Phylogenetic effects in chromosome number varied among the examined clades but were generally high. Chromosome numbers were poorly related to large scale climatic conditions, while a stronger relationship with categorical variables was found. Specifically, open, disturbed, drought-prone habitats selected for low chromosome numbers, while perennial herbs, living in shaded, stable environments were associated with high chromosome numbers. Altogether, our findings support an evolutionary role of chromosome number variation, and we argue that environmental stability favours higher recombination rates in comparison to unstable environments. In addition, by comparing the results of models testing for the evolvability of 2*n* and of *x*, we provide insight into the presumptive ecological significance of polyploidy.

## Introduction

Chromosome evolution is an integral part of plant speciation^1,2^, and chromosome variations, such as polyploidy and dysploidy, provide genetic support for ecological differentiation and adaptation^3^. The crucial role of polyploidy has been widely demonstrated^4–7^, while more recently, the study of Escudero *et al*.^8^ highlighted that dysploidy can have a higher evolutionary impact than polyploidy, in the long run.

Several models have been proposed to account for the observed patterns of chromosome number variation. Some models hypothesised a progressive change from a starting number in a decreasing (‘Fusion Hypothesis’^9^), increasing (‘Fission Hypothesis’^10,11^) or diverging series (‘Modal Hypothesis’^12^). Bickham and Baker^13^ advocated a ‘Canalisation Model’, in which rapid karyotype evolution occurs immediately after a lineage enters a new adaptive zone and is followed by slower changes through time. Imai *et al*.^14,15^ developed the ‘Minimum Interaction Theory’ (MIT hereafter), which postulates that the only evolutionary relevant mutations are those linked to germinal cells, where non-homologous chromosomes are fixed during meiosis on the nuclear membrane by telomeres forming “suspension arches”. In these cells, interactions among non-homologous chromosomes would be counter-selected in two possible ways: by an increase in the nuclear volume and/or by an increase in chromosome number paralleled by a decrease of chromosome sizes. The same authors^14,15^ demonstrated that, indeed, in some animal taxa, a trend can be observed from low to high chromosome numbers, mediated by an imbalanced fission-to-fusion ratio, at least up to a theoretical maximum^16^.

Several works recorded a negative correlation between genome size and chromosome number in plants^17,18^ but a positive correlation between genome size and nuclear volume^19^. An increase in chromosome number by recurrent polyploidy can be counteracted by reductions brought about by descending dysploidy^20^. Accordingly, a chromosome duplication after polyploidisation, causing an increase in genome size, should be followed by an increase in nuclear volume, in agreement with MIT. Fewer and larger chromosomes, e.g., those originated through descending dysploidy, should be accompanied by larger nuclear volumes to reduce non-homologous chromosome interactions during meiosis consistent with MIT^14^. However, in this case no obvious increases in genome size should be expected, unless caused by massive appearance of repetitive non-coding DNA which is very frequent in plants^3^.

Although a generalised model of chromosome number variation is lacking and despite the failure of models to fully predict available experimental evidence, the development of such a diverse range of models is suggestive, *per se*, of the high evolutionary interest of chromosome number variation.

Despite the interest in chromosome number evolution over the last decades^21–24^, in the absence of studies linking changes of chromosome number to natural selection, it is impossible to identify the adaptive function of such variation. At present, an adaptive role has been demonstrated for other genomic phenotypic traits, such as genome size^25–27^ showing that species with large genomes may be at a selective disadvantage in extreme or unstable environmental conditions. However, for chromosome number variation, evolutionary hypotheses have been raised by a limited set of preliminary works^28^. It is generally admitted that chromosome number and genome size are not positively correlated in angiosperms and gymnosperms, while such a correlation is highly significant in ferns and lycophytes^29^. Further, Grant^30^ argued that in angiosperms, species with a basic chromosome number higher than 14 should be considered paleo-polyploid, thus linking high chromosome numbers with multiple ancient rounds of whole genome duplications. In addition, a series of recent studies suggested that paleo-polyploidy is widespread across angiosperms^31^ and seed plants^32^ in general, irrespective of their chromosome number. Unbalanced (i.e., odd) chromosome numbers often cause significant phenotypic changes and severely impact plant growth and sexual fitness^33^, with the exception of apomictic plants and those with holocentric chromosomes, for which no deleterious effect (i.e., counter-selection) is expected^34,35^. It has been postulated that descendant dysploid chromosome number changes, coupled with the transition to an annual life form, are the main trend in angiosperms^34^; however, this soon resulted in an overly broad generalization, like many other karyological assumptions concerning, for instance, the direction of variation in karyotype asymmetry^36^. Stebbins^1^ also hypothesized that a chromosome number reduction by dysploidy should be expected in plants occupying pioneer habitats to avoid excessive segregation and recombination of genes. Nevertheless, protection of favourable allelic co-occurrences against recombination is also achieved by inversions - with no effects on chromosome number - in some animal taxa^37^. However, high chromosome numbers carry an increased risk of mis-segregation during nuclear division^20^. Stebbins^1^ and Darlington^38^, together with Grant^2^, agreed that low recombination rates should be favoured in individuals living in unstable environments to quickly develop populations; in contrast, environmental stability is expected to select for increased recombination rates because loss of alleles is overbalanced by those rare allelic combinations with high fitness.

In particular, given that genetic recombination acts as a trade-off between the opposing needs of immediate fitness and evolutionary adaptability^2^, chromosome number should clearly play a central role in this balance^1,39^.

To date, the possible relationship among chromosome number, plant traits, and environmental parameters has scarcely been investigated quantitatively. Accordingly, the main aim of our study was to use a dataset of 801 Italian endemic vascular plants (1364 accessions) to test these relationships. Italian endemics represent an ideal case study because complete information is available for basic chromosome number (*x*), and they share a common geographical evolutionary history^40^. Whilst we expect a significant phylogenetic signal in chromosome numbers, we specifically aim to quantitatively test the hypothesis that environmental stability and longer life cycles select for higher chromosome numbers, while unstable habitats select for lower chromosome numbers. To this end, we used a phylogenetic comparative approach that estimates phylogenetic inertia and adaptation in chromosome number based on an Ornstein-Uhlenbeck process^41^. The method considers a single trait adapting to optima influenced by continuous, randomly changing predictor variables or in response to fixed categorical niches. One of the main parameters returned by the model is the phylogenetic half-life (t_1/2_), indicating the time it takes for half the ancestral influence on a trait to evolve towards the predicted optimal phenotype^42^. Using this method, we were able to estimate relationships between chromosome numbers, plant traits and environmental factors in an evolutionary framework. Finally, by comparing the results of the different models testing for evolvability of 2*n* and of *x*, we were able to assess the relative contribution of polyploidy to 2*n* and provide insight into its presumptive ecological significance.

## Results

### Phylogenetic effect in chromosome number

The phylogenetic effects in chromosome number varied among the examined clades, but were generally moderate to large (Table 1), thus rejecting the hypothesis of species independence. Diploid chromosome numbers (2*n*) exhibited significant phylogenetic effects (t_1/2_ > 0), and the supported values (values within two log-likelihood units lower than the maximum log-likelihood) ranged from a moderate to strong phylogenetic effect, not exceeding 1.0 total length (t_1/2_ < 1), with the exceptions of Malvids and Caryophyllales. Indeed, the best estimate for Malvids was t_1/2_ = 0.01, with a support region from 0 to 0.05, suggesting that the trait evolved rapidly, nearly instantly on the timescale of this phylogeny. In contrast, the half-life value for Caryophyllales was many times the total tree length (infinity), indicating that 2*n* evolved as if by Brownian motion in this clade.

**Table 1:** Phylogenetic regression results of the evolution of log-transformed chromosome numbers (2*n* and *x*) on climatic variables in each angiosperm clade (as indicated).

Clade	Trait	Predictor	n	t_½_ (support region)	v_y_	Intercept (±SE)	Phylogenetic regression slope (±SE)	R^2^	AICc	Simple AICw*	Global AICw**
Monocots	2*n*	(single-equilibrium O–U)	132	0.14 (0.09–0.27)	0.36	3.377 ± 0.103	—	—	192.26	—	0.1249
		(Brownian motion)		—	—	3.656 ± 0.584	—	—	226.90	0.0000	0.0000
		Mean Temperature		0.12 (0.08–0.24)	0.32	4.417 ± 0.576	−0.389 ± 0.209	0.026	191.14	0.2498	0.2186
		Temperature seasonality		0.13 (0.09–0.27)	0.34	0.063 ± 2.836	0.523 ± 0.448	0.011	192.97	0.1000	0.0876
		Temperature continentality		0.12 (0.08–0.24)	0.32	1.461 ± 0.941	0.627 ± 0.308	0.031	190.46	**0.3509**	0.3071
		Annual precipitation		0.14 (0.09–0.27)	0.36	3.308 ± 1.054	0.011 ± 0.161	0.000	194.38	0.0494	0.0433
		Precipitation seasonality		0.13 (0.08–0.25)	0.32	4.239 ± 0.479	−0.222 ± 0.120	0.024	191.14	0.2498	0.2186
	*x*	(single-equilibrium O–U)		1.48 (0.48-∞)	0.33	2.498 ± 0.16	—	—	−43.56	—	0.3323
		(Brownian motion)		—	—	2.514 ± 0.213	—	—	−39.27	0.0000	0.0415
		Mean Temperature		1.48 (0.48-∞)	0.33	2.617 ± 0.280	−0.044 ± 0.086	0.002	−41.76	0.2157	0.1351
		Temperature seasonality		1.48 (0.48-∞)	0.33	2.889 ± 1.102	−0.062 ± 0.172	0.000	−41.48	0.1875	0.1174
		Temperature continentality		1.48 (0.48-∞)	0.33	2.330 ± 0.398	0.055 ± 0.119	0.002	−41.68	0.2073	0.1298
		Annual precipitation		1.48 (0.48-∞)	0.33	2.391 ± 0.438	0.016 ± 0.062	0.001	−41.53	0.1923	0.1204
		Precipitation seasonality		1.48 (0.48-∞)	0.33	2.436 ± 0.240	0.016 ± 0.047	0.001	−41.58	0.1972	0.1235
Fabids	2*n*	(single-equilibrium O–U)	106	0.26 (0.14–0.70)	0.22	3.231 ± 0.103	—	—	96.63	—	0.2571
		(Brownian motion)		—	—	3.315 ± 0.267	—	—	107.91	0.0012	0.0009
		Mean Temperature		0.28 (0.14–0.72)	0.22	2.639 ± 0.402	0.237 ± 0.154	0.026	96.61	0.3496	0.2597
		Temperature seasonality		0.28 (0.14–0.72)	0.22	7.686 ± 3.080	−0.703 ± 0.486	0.022	96.81	0.3163	0.2350
		Temperature continentality		0.28 (0.14–0.72)	0.22	5.523 ± 1.449	−0.731 ± 0.463	0.026	97.93	0.1807	0.1342
		Annual precipitation		0.26 (0.14–0.70)	0.22	4.021 ± 1.075	−0.120 ± 0.163	0.006	99.67	0.0757	0.0562
		Precipitation seasonality		0.26 (0.14–0.70)	0.22	2.895 ± 0.457	0.089 ± 0.117	0.006	99.65	0.0765	0.0568
	*x*	(single-equilibrium O–U)		0.76 (0.32-∞)	0.13	2.351 ± 0.088	—	—	−75.42	−	0.4485
		(Brownian motion)		—	—	2.367 ± 0.145	—	—	−67.58	0.0161	0.0089
		Mean Temperature		0.64 (0.32-∞)	0.11	2.034 ± 0.173	0.125 ± 0.060	0.039	−71.57	0.1187	0.0654
		Temperature seasonality		0.92 (0.36-∞)	0.15	4.207 ± 1.232	−0.985 ± 0.653	0.021	−75.40	0.8053	0.4441
		Temperature continentality		0.56 (0.28-∞)	0.11	2.443 ± 0.597	−0.031 ± 0.190	0.000	−67.43	0.0150	0.0083
		Annual precipitation		0.56 (0.28-∞)	0.11	2.283 ± 0.435	0.010 ± 0.065	0.000	−67.43	0.0150	0.0083
		Precipitation seasonality		0.72 (0.32-∞)	0.13	2.130 ± 0.205	0.059 ± 0.049	0.015	−68.82	0.0300	0.0165
Malvids	2*n*	(single-equilibrium O–U)	67	0.01 (0.00–0.05)	0.2	3.117 ± 0.057	—	—	94.07	—	0.1671
		(Brownian motion)		—	—	3.271 ± 0.7	—	—	132.41	0.0000	0.0000
		Mean Temperature		0.01 (0.00–0.06)	0.2	3.687 ± 0.956	−0.210 ± 0.351	0.013	94.93	0.1305	0.1087
		Temperature seasonality		0.01(0.00–0.05)	0.2	−0.746 ± 5.76	0.607 ± 0.905	0.016	95.27	0.1101	0.0917
		Temperature continentality		0.01(0.00–0.05)	0.2	1.161 ± 2.096	0.642 ± 0.687	0.031	94.24	0.1843	0.1535
		Annual precipitation		0.01(0.00–0.04)	0.18	0.849 ± 1.912	0.345 ± 0.291	0.050	92.97	**0.3477**	0.2896
		Precipitation seasonality		0.01(0.00–0.04)	0.2	3.958 ± 0.843	−0.229 ± 0.228	0.036	93.82	0.2273	0.1894
	*x*	(single-equilibrium O–U)		0.38 (0.14-∞)	0.09	2.303 ± 0.105	—	—	−35.62	—	0.3206
		(Brownian motion)		—	—	2.306 ± 0.267	—	—	−23.05	0.0009	0.0006
		Mean Temperature		0.28 (0.14-∞)	0.07	1.959 ± 0.291	0.123 ± 0.102	0.025	−34.29	**0.2427**	0.1649
		Temperature seasonality		0.38 (0.14-∞)	0.09	1.247 ± 1.368	0.166 ± 0.215	0.009	−33.95	0.2047	0.1391
		Temperature continentality		0.38 (0.14-∞)	0.09	2.025 ± 0.508	0.091 ± 0.163	0.005	−33.63	0.1745	0.1185
		Annual precipitation		0.38 (0.14-∞)	0.09	2.548 ± 0.425	−0.038 ± 0.063	0.005	−33.71	0.1816	0.1234
		Precipitation seasonality		0.38 (0.14-∞)	0.09	2.167 ± 0.216	0.036 ± 0.050	0.008	−33.86	0.1957	0.1330
Caryophyllales	2*n*	(single-equilibrium O–U)	140	∞ (0.28-∞)	0.18	3.402 ± 0.163	—	—	99.16	—	0.1851
		(Brownian motion)		—	—	3.402 ± 0.184	—	—	97.52	0.5157	0.4203
		Mean Temperature		∞ (0.24-∞)	0.18	3.545 ± 0.663	−0.057 ± 0.234	0.004	100.93	0.0937	0.0764
		Temperature seasonality		∞ (0.28-∞)	0.18	3.464 ± 3.411	−0.008 ± 0.538	0.007	100.92	0.0942	0.0768
		Temperature continentality		∞ (0.24-∞)	0.18	2.831 ± 1.185	0.189 ± 0.390	0.002	101.16	0.0835	0.0681
		Annual precipitation		∞ (0.32-∞)	0.16	4.272 ± 1.082	−0.129 ± 0.164	0.017	100.32	0.1272	0.1036
		Precipitation seasonality		∞ (0.28-∞)	0.18	3.078 ± 0.619	0.085 ± 0.155	0.003	101.11	0.0857	0.0698
	*x*	(single-equilibrium O–U)		∞ (0.56-∞)	0.07	2.371 ± 0.091	—	—	−258.67	—	0.0120
		(Brownian motion)		—	—	2.364 ± 0.147	—	—	−267.43	0.9718	0.9601
		Mean Temperature		∞ (0.52-∞)	0.07	2.403 ± 0.190	−0.011 ± 0.061	0.001	−256.56	0.0042	0.0042
		Temperature seasonality		1.92 (0.52-∞)	0.06	1.711 ± 0.887	0.103 ± 0.139	0.015	−257.82	0.0080	0.0079
		Temperature continentality		∞ (0.48-∞)	0.07	2.159 ± 0.308	0.070 ± 0.098	0.007	−257.19	0.0058	0.0057
		Annual precipitation		∞ (0.56-∞)	0.07	2.390 ± 0.270	−0.002 ± 0.038	0.001	−256.56	0.0042	0.0042
		Precipitation seasonality		∞ (0.56-∞)	0.07	2.256 ± 0.175	0.029 ± 0.038	0.007	−257.21	0.0059	0.0058
Lamiids	2*n*	(single-equilibrium O–U)	94	0.28 (0.16–0.84)	0.24	3.285 ± 0.106	—	—	114.43	—	0.2434
		(Brownian motion)		—	—	3.338 ± 0.302	—	—	119.99	0.0199	0.0151
		Mean Temperature		0.28 (0.16–0.82)	0.24	3.39 ± 0.609	−0.039 ± 0.225	0.000	116.59	0.1092	0.0826
		Temperature seasonality		0.28 (0.14–0.82)	0.24	1.162 ± 3.119	0.334 ± 0.49	0.005	116.18	0.1341	0.1014
		Temperature continentality		0.26 (0.14–0.80)	0.22	1.35 ± 1.069	0.628 ± 0.346	0.037	113.48	**0.5172**	0.3913
		Annual precipitation		0.28 (0.16–0.84)	0.24	2.969 ± 1.431	0.048 ± 0.216	0.001	116.57	0.1103	0.0835
		Precipitation seasonality		0.28 (0.16–0.82)	0.24	3.366 ± 0.483	−0.022 ± 0.128	0.000	116.59	0.1092	0.0826
	*x*	(single-equilibrium O–U)		0.88 (0.24-∞)	0.12	2.301 ± 0.108	—	—	26.45	—	0.2391
		(Brownian motion)		—	—	2.306 ± 0.152	—	—	25.87	0.4199	0.3195
		Mean Temperature		0.92 (0.24-∞)	0.12	2.559 ± 0.442	−0.097 ± 0.16	0.005	28.35	0.1215	0.0925
		Temperature seasonality		0.92 (0.24-∞)	0.12	3.985 ± 2.242	−0.265 ± 0.352	0.008	28.17	0.1330	0.1012
		Temperature continentality		0.88 (0.24-∞)	0.12	2.364 ± 0.783	−0.021 ± 0.253	0.000	28.62	0.1062	0.0808
		Annual precipitation		0.88 (0.24-∞)	0.12	2.048 ± 0.993	0.038 ± 0.149	0.001	28.57	0.1089	0.0828
		Precipitation seasonality		0.88 (0.24-∞)	0.12	2.191 ± 0.354	0.03 ± 0.092	0.002	28.54	0.1105	0.0841
Campanulids	2*n*	(single-equilibrium O–U)	200	0.14 (0.09–0.24)	0.16	3.142 ± 0.069	—	—	150.75	—	0.1777
		(Brownian motion)		—	—	3.26 ± 0.328	—	—	168.84	0.0000	0.0000
		Mean Temperature		0.13 (0.10–0.24)	0.14	3.814 ± 0.379	−0.233 ± 0.129	0.016	150.24	0.2789	0.2293
		Temperature seasonality		0.14 (0.09–0.25)	0.16	0.856 ± 1.927	0.361 ± 0.304	0.007	151.50	0.1485	0.1221
		Temperature continentality		0.13 (0.09–0.25)	0.14	1.766 ± 0.738	0.458 ± 0.245	0.017	149.46	**0.4119**	0.3387
		Annual precipitation		0.14 (0.09–0.24)	0.16	3.036 ± 0.667	0.016 ± 0.102	0.000	152.81	0.0771	0.0634
		Precipitation seasonality		0.14 (0.09–0.24)	0.16	3.261 ± 0.278	−0.031 ± 0.071	0.001	152.65	0.0836	0.0687
	*x*	(single-equilibrium O–U)		1.96 (0.44-∞)	0.24	2.415 ± 0.134	—	—	−71.87	—	0.0000
		(Brownian motion)		—	—	2.44 ± 0.229	—	—	−108.28	1.0000	1.0000
		Mean Temperature		∞ (0.44-∞)	0.24	3.022 ± 0.344	−0.209 ± 0.109	0.023	−70.84	0.0000	0.0000
		Temperature seasonality		∞ (0.44-∞)	0.24	0.442 ± 1.244	0.311 ± 0.195	0.017	−71.78	0.0000	0.0000
		Temperature continentality		∞ (0.44-∞)	0.24	1.667 ± 0.520	0.247 ± 0.166	0.017	−71.90	0.0000	0.0000
		Annual precipitation		∞ (0.44-∞)	0.24	2.682 ± 0.439	−0.040 ± 0.063	0.003	−70.12	0.0000	0.0000
		Precipitation seasonality		∞ (0.44-∞)	0.24	2.654 ± 0.232	−0.062 ± 0.048	0.011	−71.15	0.0000	0.0000

All phylogenetic trees are scaled to 1.0 total length. Predictor variables are given in the committed column, except when a Brownian motion or a single equilibrium O–U was fit, which has only an intercept. In the latter case, the phylogenetic half-life (t_½_, with 2-unit support interval in parentheses) is a measure of the overall effect of the phylogeny on the response variable (phylogenetic signal). In models with predictors, t_½_ indicates the time it takes the trait to evolve half the way from an ancestral state to the optimal state (rate of adaptation). Stationary variance (v_y_), intercept (±SE), slope (±SE) from phylogenetic regression and the amount of variance explained by the model (R^2^), Akaike Information Criterion (AIC) and Akaike Information Criterion weights (AICw) in comparison with the no-specific-adaptation model (single-equilibrium O–U model: 2*n* ~ 1 or *x* ~ 1) are shown. *Simple AICw is the AIC weight for each model relative to the no-predictor O–U model. **Global AICw is the AIC weight for each model relative to all models tested. For each set of models, the highest simple AICw is in bold when associated with a reduction of the phylogenetic half-life.

Compared to diploid numbers, the basic chromosome number (*x*) showed stronger phylogenetic effects with a half-life that included t_1/2_ > 1 and a supporting region that included t_1/2_ = ∞ in all clades. Furthermore, the wider support regions suggested that estimates of t_1/2_ for *x* were more uncertain than those for 2*n*.

The stronger phylogenetic effects exhibited by *x* were associated with coefficients of variation (CV) lower than 0.5, while the CV calculated for 2*n* was larger and was followed by weaker phylogenetic effects (Table 2).

**Table 2:** Mean chromosome numbers (±SD) and coefficient of variation (CV) for major clades of Italian endemics.

	2*n*		*x*	
	Mean ± sd	CV	Mean ± sd	CV
Monocots	33.7 ± 25.1	0.74	10.8 ± 5.2	0.48
Fabids	32.1 ± 17.9	0.56	10.5 ± 2.8	0.27
Malvids	26 ± 20.3	0.78	8.8 ± 2.4	0.28
Caryophyllales	27 ± 11.3	0.42	10.2 ± 2.2	0.22
Lamiids	29.7 ± 18.4	0.62	9.89 ± 3.6	0.37
Campanulids	25.6 ± 14.1	0.55	10.6 ± 4.4	0.42

### Adaptation and inertia in chromosome number

We found clear evidence for adaptation of chromosome number to environmental and morphological predictors, but in several cases, and especially for basic chromosome number (*x*), a pure Brownian motion model best explained the evolution of chromosome number on the phylogeny (Tables 1 and 3).

**Table 3:** Primary optima (±SE) of log-transformed chromosome numbers (2*n* and *x*) for each categorical predictor variable in each angiosperm clade (as indicated).

Clade	Trait	Predictor	n	t_½_ (support region)	v_y_	Primary optimum (θ ± SE)	R^2^	AICc	Simple AICw*	Global AICw**
Monocots	2*n*	(single-equilibrium O–U)	132	0.14 (0.09–0.27)	0.36	3.377 ± 0.103	—	192.26	—	0.0003
		(Brownian motion)		—	—	3.656 ± 0.584	—	226.9	0.0000	0.0000
		Growth form		0.14 (0.09–0.27)	0.36	Geophyte = 3.397 ± 0.117	0.001	194.26	0.0001	0.0001
						Perennial = 3.309 ± 0.217				
		Flower size		0.13 (0.09–0.27)	0.34	Incospicuous = 3.302 ± 0.202	0.010	195.26	0.0001	0.0001
						Small = 3.366 ± 0.113				
						Large = 4.029 ± 0.593				
		Inflorescence		0.13 (0.09–0.27)	0.34	Single = 3.399 ± 0.118	0.001	194.24	0.0001	0.0001
						Inflorescence = 3.307 ± 0.213				
		Habitat light		0.1 (0.07–0.16)	0.26	Open = 3.096 ± 0.091	0.154	175.75	**0.9844**	0.9842
						Semi = 3.872 ± 0.159				
						Forest = 3.966 ± 0.261				
		Habitat nutrient		0.1 (0.07–0.18)	0.28	Oligotrophic = 3.198 ± 0.097	0.099	184.23	0.0142	0.0142
						Mesotrophic = 3.489 ± 0.138				
						Eutrophic = 4.817 ± 0.441				
		Habitat moisture		0.11 (0.08–0.20)	0.3	Dry = 3.116 ± 0.12	0.058	189.26	0.0011	0.0011
						Moist = 3.585 ± 0.126				
						Wet = 3.986 ± 0.596				
	*x*	(single-equilibrium O–U)	132	1.48 (0.48-∞)	0.33	2.498 ± 0.16	—	−43.56	—	0.1314
		(Brownian motion)		—	—	2.514 ± 0.213	—	−39.27	0.0189	0.0164
		Growth form		1.48 (0.48-∞)	0.33	Geophyte = 2.509 ± 0.175	0.000	−41.46	0.0529	0.0460
						Perennial = 2.368 ± 0.855				
		Flower size		1.48 (0.48-∞)	0.33	Incospicuous = 2.684 ± 0.875	0.001	−39.44	0.0193	0.0167
						Small = 2.458 ± 0.191				
						Large = 2.942 ± 1.253				
		Inflorescence		1.48 (0.48-∞)	0.33	Single = 2.485 ± 0.176	0.000	−41.47	0.0532	0.0462
						Inflorescence = 2.652 ± 0.87				
		Habitat light		0.64 (0.24-∞)	0.15	Open = 2.28 ± 0.13	0.068	−46.40	**0.6257**	0.5435
						Semi = 2.861 ± 0.32				
						Forest = 3.602 ± 0.431				
		Habitat nutrient		1.00 (0.40-∞)	0.23	Oligotrophic = 2.068 ± 0.33	0.017	−41.15	0.0453	0.0394
						Mesotrophic = 2.685 ± 0.214				
						Eutrophic = 2.704 ± 0.93				
		Habitat moisture		0.92 (0.40-∞)	0.21	Dry = 1.828 ± 0.41	0.040	−43.96	0.1847	0.1605
						Moist = 2.591 ± 0.151				
						Wet = 4.744 ± 1.643				
Fabids	2*n*	(single-equilibrium O–U)	106	0.26 (0.14–0.70)	0.22	3.231 ± 0.103	—	96.63	—	0.0379
		(Brownian motion)		—	—	3.315 ± 0.267	—	107.91	0.0001	0.0001
		Growth form		0.22 (0.1–0.48)	0.20	Annual = 2.346 ± 0.672	0.064	95.33	0.0754	0.0725
						Perennial = 3.059 ± 0.116				
						Woody = 3.478 ± 0.147				
		Flower size		0.22 (0.12–0.50)	0.20	Incospicuous = 3.066 ± 0.118	0.051	96.34	0.0455	0.0438
						Small = 3.461 ± 0.192				
						Large = 3.72 ± 0.361				
		Inflorescence		0.26 (0.14–0.70)	0.20	Single = 3.238 ± 0.109	0.000	98.75	0.0136	0.0131
						Inflorescence = 3.171 ± 0.339				
		Habitat light		0.22 (0.12–0.46)	0.18	Open = 3.079 ± 0.1	0.104	91.00	**0.6568**	0.6319
						Semi = 3.718 ± 0.428				
						Forest = 3.976 ± 0.272				
		Habitat nutrient		0.24 (0.12–0.50)	0.20	Oligotrophic = 3.037 ± 0.125	0.060	95.23	0.0792	0.0762
						Mesotrophic = 3.511 ± 0.17				
						Eutrophic = 3.913 ± 0.63				
		Habitat moisture		0.24 (0.14–0.52)	0.20	Dry = 3.043 ± 0.122	0.070	94.25	0.1293	0.1244
						Moist = 3.464 ± 0.186				
						Wet = 3.914 ± 0.387				
Fabids	*x*	(single-equilibrium O–U)	106	0.76 (0.32-∞)	0.13	2.351 ± 0.088	—	−75.42	—	0.1643
		(Brownian motion)		—	—	2.367 ± 0.145	—	−67.58	0.0039	0.0033
		Growth form		0.64 (0.32-∞)	0.11	Annual = 1.654 ± 0.848	0.043	−75.58	0.2129	0.1780
						Perennial = 2.231 ± 0.101				
						Woody = 2.671 ± 0.181				
		Flower size		0.64 (0.28-∞)	0.11	Incospicuous = 2.278 ± 0.103	0.026	−73.80	0.0874	0.0731
						Small = 2.362 ± 0.379				
						Large = 2.614 ± 0.226				
		Inflorescence		0.76 (0.32-∞)	0.13	Single = 2.36 ± 0.09	0.002	−73.45	0.0734	0.0613
						Inflorescence = 2.139 ± 0.492				
		Habitat light		0.64 (0.32-∞)	0.11	Open = 2.268 ± 0.088	0.052	−76.55	**0.3458**	0.2890
						Semi = 2.753 ± 0.445				
						Forest = 2.988 ± 0.305				
		Habitat nutrient		0.64 (0.28-∞)	0.11	Oligotrophic = 2.2 ± 0.106	0.046	−75.88	0.2474	0.2068
						Mesotrophic = 2.659 ± 0.176				
						Eutrophic = 2.982 ± 0.688				
		Habitat moisture		0.76 (0.32-∞)	0.13	Dry = 2.305 ± 0.108	0.005	−71.60	0.0291	0.0243
						Moist = 2.494 ± 0.239				
						Wet = 2.511 ± 0.456				
Malvids	2*n*	(single-equilibrium O–U)	67	0.01 (0.00–0.05)	0.2	3.117 ± 0.057	—	94.07	—	0.0335
		(Brownian motion)		—	—	3.271 ± 0.7	—	132.41	0.0000	0.0000
		Growth form		0.01 (0.00–0.04)	0.2	Annual = 2.83 ± 0.313	0.072	93.69	0.0420	0.0406
						Perennial = 3.093 ± 0.057				
						Woody = 3.53 ± 0.2				
		Flower size		0.01 (0.00–0.05)	0.2	Small = 3.028 ± 0.141	0.007	95.86	0.0142	0.0137
						Large = 3.134 ± 0.062				
		Habitat light		0.01 (0.00–0.02)	0.16	Open = 3.083 ± 0.054	0.153	87.61	**0.8775**	0.8481
						Semi = 3.122 ± 0.188				
						Forest = 4.167 ± 0.302				
		Habitat nutrient		0.01 (0.00–0.04)	0.18	Oligotrophic = 3.047 ± 0.065	0.083	93.53	0.0455	0.0439
						Mesotrophic = 3.466 ± 0.167				
						Eutrophic = 3.331 ± 0.278				
		Habitat moisture		0.01 (0.00–0.05)	0.18	Dry = 3.063 ± 0.062	0.053	95.09	0.0208	0.0201
						Moist = 3.352 ± 0.135				
						Wet = 3.181 ± 0.223				
	*x*	(single-equilibrium O–U)	67	0.38 (0.14-∞)	0.09	2.303 ± 0.105	—	−35.62	—	0.0006
		(Brownian motion)		—	—	2.306 ± 0.267	—	−23.05	0.0000	0.0000
		Growth form		0.16 (0.10–0.66)	0.05	Annual = 2.053 ± 0.33	0.096	−35.29	0.0005	0.0005
						Perennial = 2.139 ± 0.073				
						Woody = 2.471 ± 0.103				
		Flower size		0.38 (0.14-∞)	0.09	Small = 2.253 ± 0.27	0.001	−33.40	0.0002	0.0002
						Large = 2.309 ± 0.111				
		Habitat light		0.28 (0.12-∞)	0.07	Open = 2.264 ± 0.091	0.015	−32.03	0.0001	0.0001
						Semi = 2.207 ± 0.487				
						Forest = 2.58 ± 0.299				
		Habitat nutrient		0.22 (0.10–0.42)	0.05	Oligotrophic = 2.151 ± 0.073	0.263	−50.57	**0.9977**	0.9971
						Mesotrophic = 2.919 ± 0.168				
						Eutrophic = 3.195 ± 0.361				
		Habitat moisture		0.2 (0.12–0.90)	0.05	Dry = 2.153 ± 0.079	0.110	−37.65	0.0016	0.0016
						Moist = 2.462 ± 0.105				
						Wet = 2.922 ± 0.384				
Caryophyllales	2*n*	(single-equilibrium O–U)	140	∞ (0.28-∞)	0.18	3.402 ± 0.163	—	99.16	—	0.1270
		(Brownian motion)		—	—	3.402 ± 0.184	—	97.52	0.3304	0.2884
		Growth form		∞ (0.28-∞)	0.18	Perennial = 3.408 ± 0.165	0.001	101.43	0.0468	0.0408
						Woody = 2.992 ± 1.763				
		Flower size		∞ (0.28-∞)	0.18	Small = 3.569 ± 0.966	0.001	101.24	0.0514	0.0449
						Large = 3.360 ± 0.286				
		Habitat light		∞ (0.28-∞)	0.18	Open = 3.402 ± 0.164	0.001	101.28	0.0504	0.0440
						Semi = 3.449 ± 3.951				
		Habitat nutrient		1.92 (0.24-∞)	0.16	Oligotrophic = 3.344 ± 0.164	0.023	102.12	0.0331	0.0289
						Mesotrophic = 8.275 ± 8.812				
						Eutrophic = 4.905 ± 1.441				
		Habitat moisture		1 (0.08-∞)	0.06	Dry = 2.058 ± 0.54	0.099	96.74	0.4879	0.4259
						Moist = 3.6 ± 0.141				
	*x*	(single-equilibrium O–U)	140	∞ (0.56-∞)	0.07	2.371 ± 0.091	—	−258.67	—	0.0006
		(Brownian motion)		—	—	2.364 ± 0.147	—	−267.43	0.0462	0.0461
		Growth form		1.84 (0.48-∞)	0.04	Perennial = 2.341 ± 0.072	0.167	−273.48	**0.9520**	0.9515
						Woody = 4.812 ± 0.540				
		Flower size		∞ (0.56-∞)	0.07	Small = 1.794 ± 0.364	0.024	−258.96	0.0007	0.0007
						Large = 2.486 ± 0.125				
		Habitat light		∞ (0.56-∞)	0.07	Open = 2.373 ± 0.092	0.001	−256.61	0.0002	0.0002
						Semi = 2.037 ± 1.299				
		Habitat nutrient		1.92 (0.60-∞)	0.06	Oligotrophic = 2.407 ± 0.117	0.043	−258.67	0.0006	0.0006
						Mesotrophic = 0.152 ± 1.216				
						Eutrophic = 1.976 ± 0.588				
		Habitat moisture		∞ (0.56-∞)	0.07	Dry = 3.028 ± 0.584	0.013	−257.84	0.0004	0.0004
						Moist = 2.342 ± 0.095				
Lamiids	2*n*	(single-equilibrium O–U)	94	0.28 (0.16–0.84)	0.24	3.285 ± 0.106	—	114.43	—	0.0796
		(Brownian motion)		—	—	3.338 ± 0.302	—	119.99	0.0054	0.0049
		Growth form		0.26 (0.14–0.80)	0.22	Annual = 2.636 ± 0.561	0.036	115.60	0.0482	0.0443
						Perennial = 3.277 ± 0.1				
						Woody = 4.005 ± 0.548				
		Flower size		0.26 (0.14–0.74)	0.22	Small = 2.789 ± 0.416	0.016	115.22	0.0582	0.0536
						Large = 3.306 ± 0.099				
		Inflorescence		0.26 (0.14–0.70)	0.22	Single = 3.31 ± 0.099	0.026	114.32	0.0913	0.0841
						Inflorescence = 2.594 ± 0.457				
		Habitat light		0.26 (0.14–0.74)	0.2	Open = 3.149 ± 0.105	0.082	111.38	**0.3972**	0.3656
						Semi = 3.559 ± 0.297				
						Forest = 3.909 ± 0.259				
		Habitat nutrient		0.24 (0.14–0.60)	0.2	Oligotrophic = 3.146 ± 0.102	0.077	111.91	0.3047	0.2805
						Mesotrophic = 3.638 ± 0.204				
						Eutrophic = 3.887 ± 0.38				
		Habitat moisture		0.2 (0.12–0.50)	0.2	Dry = 3.085 ± 0.146	0.060	114.24	0.0950	0.0875
						Moist = 3.292 ± 0.111				
						Wet = 3.776 ± 0.261				
	*x*	(single-equilibrium O–U)	94	0.88 (0.24-∞)	0.12	2.301 ± 0.108	—	—	—	0.0352
		(Brownian motion)		—	—	2.306 ± 0.152	—	25.87	0.0488	0.0470
		Growth form		1.52 (0.24-∞)	0.16	Annual = 0.96 ± 1.719	0.089	24.58	0.0930	0.0897
						Perennial = 2.293 ± 0.118				
						Woody = 6.071 ± 1.632				
		Flower size		1.04 (0.24-∞)	0.12	Small = 0.356 ± 0.782	0.085	22.53	0.2591	0.2500
						Large = 2.33 ± 0.108				
		Inflorescence		1.04 (0.24-∞)	0.12	Single = 2.325 ± 0.108	0.067	23.85	0.1339	0.1292
						Inflorescence = 0.462 ± 0.83				
		Habitat light		1.24 (0.24-∞)	0.12	Open = 2.237 ± 0.111	0.121	22.14	0.3149	0.3038
						Semi = 2.256 ± 0.656				
						Forest = 3.839 ± 0.572				
		Habitat nutrient		0.44 (0.20–1.92)	0.06	Oligotrophic = 2.198 ± 0.077	0.107	24.02	0.1230	0.1187
						Mesotrophic = 2.606 ± 0.185				
						Eutrophic = 2.961 ± 0.315				
		Habitat moisture		0.56 (0.20-∞)	0.08	Dry = 1.928 ± 0.198	0.058	27.02	0.0274	0.0265
						Moist = 2.402 ± 0.1				
						Wet = 2.119 ± 0.332				
Campanulids	2*n*	(single-equilibrium O–U)	200	0.14 (0.09–0.24)	0.16	3.142 ± 0.069	—	150.75	—	0.0413
		(Brownian motion)		—	—	3.26 ± 0.328	—	168.84	0.0000	0.0000
		Growth form		0.13 (0.10–0.23)	0.14	Annual = 2.841 ± 0.351	0.030	149.07	0.0997	0.0956
						Perennial = 3.106 ± 0.066				
						Woody = 3.497 ± 0.166				
		Flower size		0.11 (0.07–0.21)	0.14	Small = 3.102 ± 0.06	0.026	148.31	0.1459	0.1398
						Large = 3.702 ± 0.255				
		Inflorescence		0.11 (0.08–0.21)	0.14	Single = 3.525 ± 0.197	0.021	149.26	0.0907	0.0870
						Inflorescence = 3.097 ± 0.061				
		Habitat light		0.12 (0.08–0.21)	0.14	Open = 3.092 ± 0.064	0.037	147.82	0.1863	0.1787
						Semi = 3.27 ± 0.205				
						Forest = 3.886 ± 0.292				
		Habitat nutrient		0.13 (0.09–0.22)	0.14	Oligotrophic = 3.065 ± 0.069	0.044	146.03	**0.4561**	0.4372
						Mesotrophic = 3.301 ± 0.142				
						Eutrophic = 3.882 ± 0.287				
		Habitat moisture		0.14 (0.09–0.24)	0.16	Dry = 3.086 ± 0.077	0.014	152.16	0.0213	0.0204
						Moist = 3.252 ± 0.166				
						Wet = 3.442 ± 0.226				
Campanulids	*x*	(single-equilibrium O–U)		1.96 (0.44-∞)	0.24	2.415 ± 0.134	—	−71.87	—	0.0000
		(Brownian motion)		—	—	2.44 ± 0.229	—	−108.28	1.0000	1.0000
		Growth form		1.88 (0.44-∞)	0.22	Annual = 2.317 ± 2.277	0.006	−71.40	0.0000	0.0000
						Perennial = 2.395 ± 0.131				
						Woody = 4.421 ± 1.050				
		Flower size		∞ (0.36-∞)	0.22	Small = 2.345 ± 0.130	0.051	−77.53	0.0000	0.0000
						Large = 9.762 ± 2.578				
		Inflorescence		∞ (0.36-∞)	0.24	Single = 3.836 ± 1.114	0.011	−71.43	0.0000	0.0000
						Inflorescence = 2.350 ± 0.143				
		Habitat light		1.16 (0.32-∞)	0.14	Open = 2.284 ± 0.197	0.044	−74.07	0.0000	0.0000
						Semi = 2.875 ± 0.755				
						Forest = 3.818 ± 0.711				
		Habitat nutrient		1.16 (0.32-∞)	0.14	Oligotrophic = 2.294 ± 0.123	0.049	−74.83	0.0000	0.0000
						Mesotrophic = 2.634 ± 0.476				
						Eutrophic = 4.298 ± 0.700				
		Habitat moisture		1.88 (0.40-∞)	0.22	Dry = 2.321 ± 0.139	0.029	−72.05	0.0000	0.0000
						Moist = 2.645 ± 0.903				
						Wet = 4.334 ± 0.915				

The niches of each predictor variable were mapped onto the internal branches of the phylogeny using a parsimony reconstruction. All phylogenies are scaled to a total length of 1. Predictor variables are given in the column, except when a Brownian motion or a single equilibrium O–U was fit, which has only an intercept. Stationary variance (v_y_) and the amount of variance explained by the model (R^2^), Akaike Information Criterion (AIC) and Akaike Information Criterion weights (AICw) in comparison with the no-specific-adaptation model (single-equilibrium O–U model: 2*n* ~ 1 or *x* ~ 1) are also shown. *Simple AICw is the AIC weight for each model relative to the no-predictor O–U model. **Global AICw is the AIC weight for each model relative to all models tested. For each set of models, the highest simple AICw is in bold when associated with a reduction of the phylogenetic half-life.

The effects of the continuous (climatic) predictors were generally weak. Indeed, only eight of the 60 models that used climatic predictors had lower AICc and lower half-life (indicating that not all the phylogenetic effect was due to phylogenetic inertia) compared to the model without the predictor (Table 1). Nevertheless, even in these cases (all these models refer to 2*n*), the half-life reduction was small and the AICc decrease never exceeded 2 units which indicated that there was a weak tendency for chromosome numbers to evolve towards the optimum. Hence, these results should be regarded with caution as they represent only a tentative indication of climatic effects on chromosome numbers.

In the eight models mentioned above, the climatic variables that explained variation in chromosome number, at least marginally, were mean temperature, temperature continentality and precipitation seasonality. Overall, the association of mean temperature with chromosome number was negative with the slope of the phylogenetic regression nearly flat in Caryophyllales and Lamiids, while it was steeper in other clades, despite explaining less than 4% of the variance. However, although the relationship was not significant, it was positive in Fabids. A negative relationship with chromosome number was also found for precipitation seasonality, while temperature continentality was generally positively associated with chromosome number.

**Figure 1:**
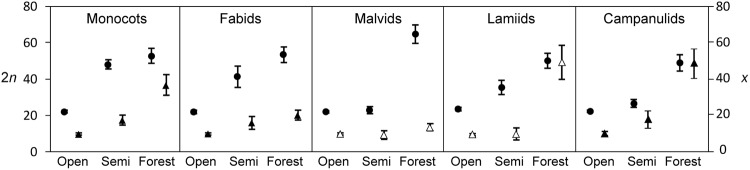
Adaptation of chromosome number (2*n*: circles and *x*: triangles) to habitat light. Mean ± SE [back transformed] of primary equilibria for the three habitat categories (open, semi-shaded and forest) mapped on the phylogeny using parsimony reconstruction. Empty symbols show estimates obtained from a model that is not outperforming a model with a single optima.

For categorical predictors, the relationship with both 2*n* and *x* was overall robust with 32 out of the 84 models outperforming the model without the predictor (Table 3). Chromosome number was mostly affected by the habitat categories (light, moisture and nutrients), but in some clades, also by morphological categories (growth form and flower size). The gain in AICc values for the outperforming models attained more than 2 units with the percentage of the variance explained largely exceeding 5%. These models also exhibited a significant reduction in half-life, which indicated that chromosome number evolved in response to categorical variables while some, but not all, of the phylogenetic effects in chromosome number is due to phylogenetic inertia. Specifically, open habitat selected for low chromosome numbers while shaded, stable environments (e.g., forest) selected for higher chromosome numbers in Monocots, Fabids, and Campanulids (for both 2*n* and *x*) and in Malvids and Lamiids (for 2*n* only) (Fig. 1). Habitat moisture and nutrient availability were positively associated with chromosome number, although some optimal values (θ) had little biological meaning. In particular, this lack of biological meaning applies to eutrophic and wet categories owing to the low number of branches attributed to these categories. Hence, we regard these results with caution; nevertheless, the overall patterns of the models were robust and understandable. Chromosome number was also positively related with plant morphological traits, namely growth form (life forms with longer life cycles had higher chromosome numbers) and flower size but in a few cases, was also negatively associated with flowers clustered into a flower-like inflorescence.

## Discussion

### Adaptation and inertia in chromosome number

We found evidence for adaptation of chromosome number to environmental and morphological predictors, especially for 2*n*, while, not surprisingly, *x* exhibited a lower degree of variation and less significant adaptive evidence, providing insight into the possible ecological significance of polyploidy. Therefore, although the majority of variation remains unexplained, a Brownian motion process alone is certainly inadequate to explain the evolution of chromosome number^42,43^.

Our study indicates that phylogenetic inertia is a significant component in all models (t_1/2_ > 0). Nevertheless, categorical predictors and, to a lesser extent, some climatic variables (mean temperature and precipitation seasonality) supported the hypothesis^1,2,38^ that environmental instability and seasonality select for low chromosome numbers (i.e., decreased recombination rates). Specifically, open, disturbed, drought-prone habitats selected for low chromosome numbers, while shaded, stable environments with good availability of water and nutrients selected for high chromosome numbers and consequently for increased recombination rates^2^.

In addition, in agreement with Bell^44^ and Stebbins^1^, who argued that rapid reproductive cycles correlate with low chromosome numbers, we found that low chromosome numbers are associated with small flowers densely clustered in inflorescences. In contrast, we found that higher chromosome numbers are linked to perennial herbs, especially geophytes, in agreement with previous studies^1,17,45^.

From our results, it seems that chromosome number is poorly related to large scale climatic conditions. Only three climate variables had a relationship with the chromosome number, so that species with higher chromosome numbers tend to occur in sites with lower annual temperature, lower precipitation seasonality and higher continentality, consistent with previous work^46^. Nevertheless, the effects of climatic predictors are best explained by models of 2*n* evolution, indicating the relative contribution of polyploidy and its ecological significance to diploid chromosome number. In addition, among categorical predictors, models fit on 2*n* exhibited stronger association with environmental and morphological predictors than the models fit on *x*, again suggesting a putative ecological significance of polyploidy. The increase of polyploid taxa with latitude and in colder sites with continental climates has been already shown by previous studies^34,47–49^, albeit questioned by others^50–52^. The recent availability of large chromosome number databases^21,53^ has spurred further research on this subject, spanning a substantially wider taxonomic space and confirming this trend across the whole Arctic flora^54^ and at other geographical scales^22^.

### Phylogenetic effect in chromosome number and clade-specific implications

In this study, we found phylogenetic effects in chromosome number ranging from moderate to large. This finding is congruent with data published previously^23^, which highlighted that closely related species share similar patterns of chromosome number variation. Despite this, chromosome number *per se* has little systematic significance, because it is well known that many taxa share the same chromosome number across different groups^1^. Further, the use of other analytical approaches to measuring phylogenetic signal (e.g., Blomberg’s K andPagel’s lambda) returned highly congruent results with patterns of phylogenetic effects higher for basic chromosome number (*x*) than those for 2*n* (Table S3).

The degree of chromosome number variation detected here allows for the investigation of possible mechanisms regulating chromosome number together with genome size. Indeed, the six considered clades, despite their origin from ancestors with very small genomes^55,56^, show remarkable differences in genome size, which is larger on average in Monocots (1C = 11.9 pg) and Campanulids (1C = 4.03 pg) than in Fabids (1C = 2.35 pg), Lamiids (1C = 2.32), Caryophyllales (1C = 1.7 pg), and Malvids (1 C = 1.45 pg) (data from^57^). Hence, in the first two clades, large nuclear volumes^19^ allow an increase in chromosome numbers and/or chromosome sizes, in agreement with the MIT^14^. In contrast, in the remaining four clades, an increase in chromosome number should be paralleled by the occurrence of smaller chromosomes in the absence of significantly larger genomes and nuclear volumes, again consistent with the MIT^14^. Accordingly, a reduction of chromosome number by descending dysploidy should be seriously constrained by limited nuclear volumes in Fabids, Lamiids, Caryophyllales, and Malvids. Indeed, in our dataset, these clades show smaller CV values concerning *x*, linked to a lower frequency of dysploidy than that observed in Monocots and Campanulids.

Monocots exhibit a high variation in chromosome number and include genera (e.g., in Poales) in which holocentric chromosomes occur, which is a peculiar condition allowing for rapid diversification and an extended range of chromosome numbers^58^. However, the high frequency of geophytes in this clade also explains the obtained results, especially for 2*n*. Indeed, in geophytes, large cells are assumed to be an advantage during the rapid development of the plant body and cell expansion^59^; as a consequence, they show higher tolerance to genome duplication with a high frequency of polyploids^60^. Under the assumptions of^19^, the occurrence of polyploidy in Monocots and Campanulids should be accompanied by significantly larger nuclear volumes, given their larger average genome sizes^57^. If polyploidisation events are evolutionarily followed by descending dysploidy events, the consequent reduction of chromosome number and simultaneous increase of chromosome size in these two clades should not be subjected to selective constraint under MIT^14^. Finally, in Monocots the co-occurrence of massive polyploidisation and large genome size variation points towards evolutionary phenomena linked to the large genome constraint hypothesis^27^.

The performance of the models on Malvids might be biased by low sampling^61^, while in Fabids, the results are partly obscured by the co-occurrence of dysploidy and polyploidy phenomena in our dataset, especially in Mediterranean taxa (e.g., *Genista*). In Caryophyllales, results are biased by the predominance of taxa belonging to the genus *Limonium*, but model fits on 2*n* and *x* are largely comparable. In this latter clade, habitat patchiness/stochasticity and high frequency of hybridization, rather than polyploidy, might have influenced speciation and chromosome number changes.

In the present study, several associations among chromosome number, plant traits and environmental factors were found. However, as mentioned by other authors^28,62^, there is no reason to expect chromosome number *per se* to affect plant fitness. Instead, chromosome number variation can be driven by cellular processes that affect meiosis and mitosis^14,20^. Hence, we also interpret the evolution towards an optimal state as a karyotypic equilibrium determined by mutation rates^63^, by nuclear division dynamics^14,20^, and possibly by epigenomic surveillance systems^18^, rather than an adaptive optimum.

Albeit limited in sample size, at first glance, our results showed that the evolutionary process is homogeneous across the phylogeny^41^ among different clades of Italian endemics. Yet, having demonstrated that phylogenetic inertia is a significant component in chromosome number evolution, future studies can be extended to larger samples and also using different approaches^64^, where not only the primary optima but also the α-parameter (strength of selection) and σ-parameter (strength of drift) differ among niches, allowing clade-dependent rates of adaptation to be tested^65^.

In conclusion, our study presents non-stochastic demonstrations for chromosome number variation, and we argue that environmental stability favors higher recombination rates in comparison to unstable environments. In addition, whilst phylogeny is a strong predictor of trait values, especially for *x*, we highlight that a simple phylogenetic explanation is inadequate to account for its variation in 2*n* and *x*.

## Methods

### Chromosome data

Chromosome counts for 1364 accessions of 801 vascular plants endemic to Italy have been extracted from Chrobase.it (http://bot.biologia.unipi.it/chrobase/)^66^. Chrobase.it is an online dataset of chromosome counts for the Italian vascular flora^21^, hosting cytogenetic data for endemic and non-endemic species. For this study we only selected counts of endemic plants because they are the most sensitive components of a flora, often being restricted to ecologically selective habitats^67^, for which we are confident that the environmental variables calculated in the present study can be a good proxy for the total ecological requirements of the species. Most counts in Chrobase.it are associated with an exact geographic locality. For those chromosome counts lacking precise information (<10%), we identified an approximate locality based on the restricted distribution range of the species^68,69^.

Mean chromosome numbers were estimated for each species, while within-species variation to be incorporated into the phylogenetic analysis was not estimated separately for each species, because within-species samples were limited to a few counts^70^. Hence, we estimated the pooled variance across the species and used it, weighted by sample size, to estimate the observation variance of the individual species, as recommended by^71^. Preliminary analyses revealed that log transformation improved model fit by over 500 log-likelihood units relative to untransformed data, thus mean chromosome numbers were log transformed prior to analysis^62^.

Chromosome number evolution was analysed on a dataset separated into 6 major angiosperm clades^72^ (Monocots, Fabids, Malvids, Caryophyllales, Lamiids, and Campanulids) to guarantee an adequate number of taxa per clade (ranging from a minimum of 67 to a maximum of 200). This analysis allowed us to evaluate whether chromosome number evolution proceeded differently or homogeneously along different evolutionary histories. The complete data set assembled for the present study is reported in supplementary Table S2.

### Phylogeny

**Figure 2:**
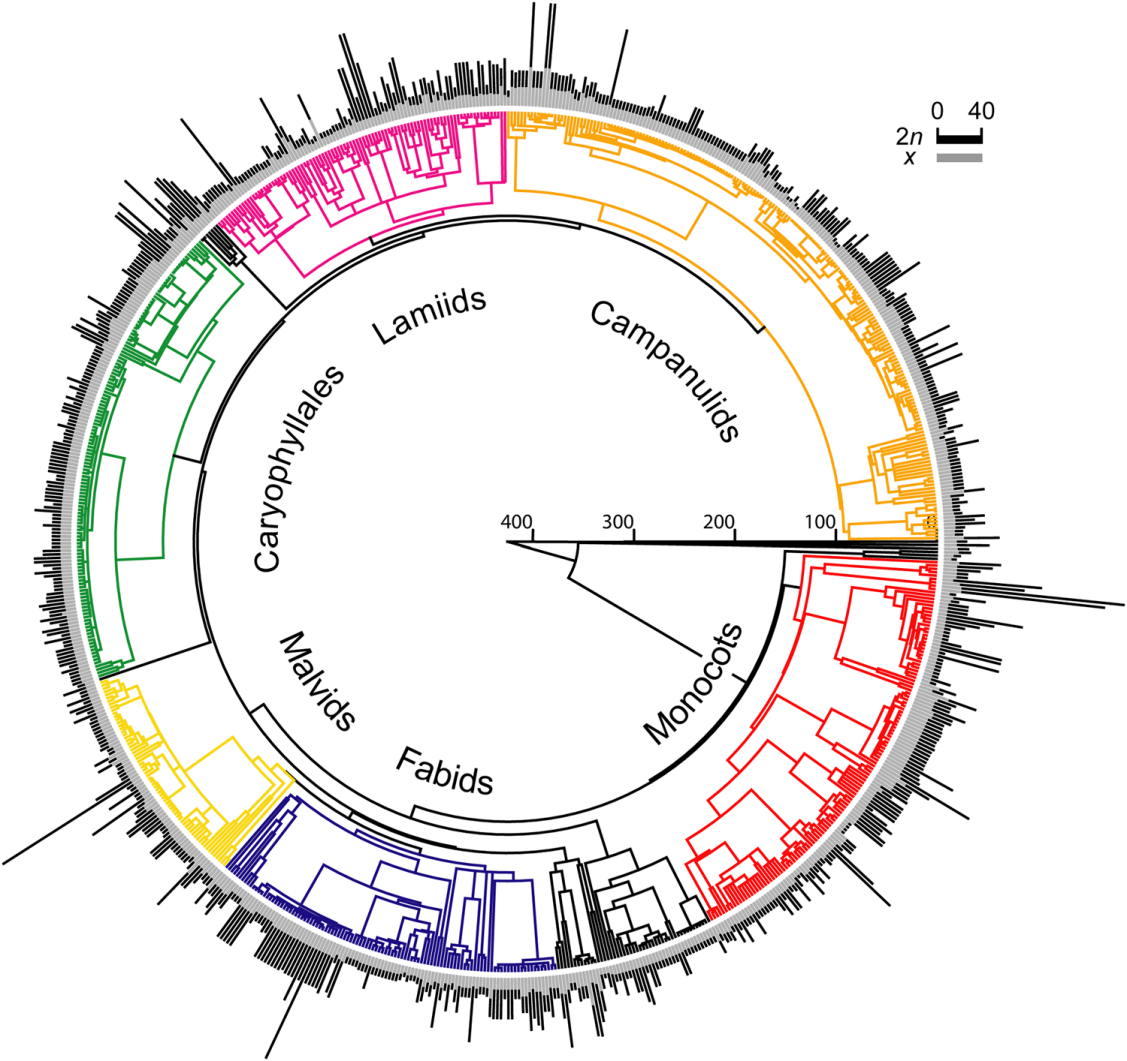
Ultrametric phylogenetic tree of 801 vascular plants endemic to Italy. The clades considered in this study are coloured and named according to^72^. Bars at the tips of the tree are proportional to the mean value of 2*n* and *x* per species (as indicated).

We compiled a phylogeny using the dated, ultrametric supertree for 4685 European vascular plants (DaPhnE 1.0 supertree)^73^ based on 518 recent molecular phylogenies. We first completed this supertree for the 281 species in our data set that were absent from the supertree (or for which related species were missing in the supertree) following the methods of Durka and Michalski^73^, using more than 90 phylogenetic and systematic studies. We then reduced this tree to the 801 species in our dataset. The complete list of sources used in this paper is reported in supplementary Table S1. Mean diploid (2*n*) and basic (*x*, see^74^) chromosome numbers for each taxon were visualized on the phylogenetic tree (Figs 2 and S1) with the plotsimmap function in the package phytools^75^ of R^76^. Basic chromosome numbers (*x*) were obtained by a taxon by taxon screening of relevant karyological literature previously published by^40^.

### Climatic, ecological and morphological data

Climatic data associated with the sampling sites were downloaded from the Worldclim database at 2.5 min scale (http://www.worldclim.org^77^). The climate data considered were: mean annual temperature (°C), temperature seasonality (SD × 100), temperature continentality (°C), mean annual precipitation (mm) and precipitation seasonality (coefficient of variation). Means and standard deviations were estimated for each species within a buffer of 10 min over georeferenced sites using QGIS v. 2.18 (Quantum GIS, http://www.qgis.org).

Data on morphological traits and habitat characteristics for each of the considered species were retrieved from the literature^78,79^ (Table S2). To characterise the relevant features of vegetative morphology and reproductive strategies, we considered the growth form (annual herb, geophytes, perennial herb, and woody), the flower size (large >5 mm, small <5 mm, and inconspicuous ≪1 mm) and whether the flowers are densely clustered in inflorescences. Habitats were classified using ecologically meaningful categorical variables in terms of stability vs. instability of the habitats and reliable vs. unreliable resource availability. For this purpose, we classified the species into three categories according to soil moisture (dry [1–4], moist [5–8], and wet [9–12]), habitat light (open [9–12], semi-shaded [5–8], and forest [1–4]) and soil nutrient contents (oligo- [1–3], meso- [4–6], and eu-trophic [7–9]) based on Ellenberg indicator values (grouped as reported in square brackets) reported for the species^79^.

### Phylogenetic comparative analysis

We investigated whether the evolution towards optima of diploid (2*n*) and basic (*x*) chromosome numbers is influenced by climatic variables (continuous predictors), habitat characteristics or plant traits (categorical predictors) within different angiosperm clades.

Thus, we used the phylogenetic comparative method implemented in the R program SLOUCH, established to study adaptive evolution of a trait along a phylogenetic tree^41^. The method assumes that the response trait evolves as if by an Ornstein–Uhlenbeck model of adaptive evolution towards a primary optimum θ, defined as the optimal state that species will approach in a given niche^42^.

Whilst we illustrated the overall evolutionary relationships among clades in Fig. 2, the phylogenetic comparative analyses were run only on the subtrees associated with each clade. Phylogenetic trees are scaled to 1.0 (from the root to the tip of the ultrametric tree) for an easier interpretation of parameter estimates^41^. The two main parameters returned by the model are the phylogenetic half-life (t_1/2_) and the stationary variance (v_y_). Phylogenetic half-life indicates the time it takes for half the ancestral influence on a trait to evolve towards the predicted optimal phenotype^42^. A half-life greater than zero means that adaptation is not instantaneous, while t_1/2_ = 0 means that there is no evolutionary lag. The stationary variance is the stochastic component of the model and can be considered as evolutionary changes in the response trait induced by genetic drift.

Phylogenetic half-life in a model that only includes the intercept is an estimate of the phylogenetic effect in the response trait. In such a model, a half-life = zero means that the response variable is not phylogenetically clustered, while a half-life >0 suggests that there is an influence of phylogeny on the data; a half-life with high values can be attributed to an underlying continuous Brownian motion process.

The intercept-only model is contrasted with a model that also includes a predictor variable. This type of model is regarded as an adaptation model because it tests whether the response traits evolve towards optima influenced by a predictor. By comparing a model with and without the inclusion of predictor variables, it is possible to determine how much of the phylogenetic signal can be attributed to phylogenetic inertia (i.e., resistance to adaptation). No reduction in t_1/2_ suggests that the phylogenetic signal can be entirely attributed to phylogenetic inertia; in contrast, when a trait evolves in response to a variable, a reduction in the half-life for the response trait (and/or of its support interval) should be observed.

The adaptation models, which include continuous predictors (i.e., the climatic variables in our study), are fitted using maximum likelihood for estimation of t_1/2_ and v_y_ and generalized least squares for estimation of the regression parameters in an iterative procedure^41^. In addition, the SLOUCH model assumes that the predictors have a longer phylogenetic half-life than the model residuals, and this is well supported by the variables involved in our study. The model returns parameters of an optimal regression and of a phylogenetic regression. The former is the relationship between the response and predictor variable that is predicted to evolve free of ancestral influence (absence of inertia). Therefore, the slope of this regression must be steeper than that of the phylogenetic regression.

To evaluate the effect of the categorical predictors on the evolution of chromosome number, the ANOVA and ANCOVA extensions implemented in SLOUCH were used. Categorical predictors were mapped onto the phylogeny using parsimony reconstruction.

We used Akaike’s Information Criterion (AIC) and AIC weights (AICw) to compare intercept-only models to the adaptation models. Use of AICw standardizes the weight of evidence in favour of each alternative model between 0 and 1^80^. We also reported AIC weights for each model relative to the set of models evaluated.

Finally, model interpretations were based on comparisons of t_1/2_ and v_y_ of the adaptation models with the intercept-only model and with a pure Brownian motion model, along with the amount of variation in chromosome number that the models explain. All statistical analyses have been carried out in R v3.2.3^76^.

## Electronic supplementary material

Supplementary information

## Data Availability

All data analysed during this study are included in this published article (and its Supplementary Information files).
