# Supplementary material for: Unscrambling phylogenetic effects and ecological determinants of chromosome number in major angiosperm clades
Source: Sci Rep. 2018 Sep 24;8:14258. doi: 10.1038/s41598-018-32515-x (PMC6155329; doi:10.1038/s41598-018-32515-x)
Supplement: Supplementary file 1 — Supplementary information [file 41598_2018_32515_MOESM1_ESM.pdf]

Article title: Unscrambling phylogenetic effects and ecological determinants of chromosome number in major angiosperm clades

Authors: Angelino Carta, Gianni Bedini and Lorenzo Peruzzi

Supplementary information Tables S1, S2, S3, Figure S1 and Phylogenetic tree.

Table S1. References used for resolving lower taxonomic levels.

Table S2. Detailed information for species included in the study.

Table S3. Summary of phylogenetic signal using different comparativemethods for diploid chromosome number (2*n*) and basic chromosome number (*x*).

Figure S1. Phylogenetic tree used in the study with named tips.

Phylogenetic tree. Phylogenetic tree used in the study in Newick format.

Table S1. References used for resolving lower taxonomic levels

Aguilar, J. F., & Feliner, G. N. (2003). Additive polymorphisms and reticulation in an ITS phylogeny of thrifts (*Armeria*, Plumbaginaceae). *Molecular phylogenetics and evolution*, *28*(3), 430-447.

Aquaro, G., Caparelli, K. F., & Peruzzi, L. (2008). The genus *Taraxacum* (Asteraceae) in Italy. I. A systematic study of *Taraxacum* sect. *Palustria*. *Phytologia Balcanica*, *14*, 61-67.

Arias, T., & Pires, J. C. (2012). A fully resolved chloroplast phylogeny of the brassica crops and wild relatives (Brassicaceae: Brassiceae): Novel clades and potential taxonomic implications. *Taxon*, *61*(5), 980-988.

Arrigoni, P. V., & Diana, S. (1999). Karyology, chorology and bioecology of the genus *Limonium* (Plumbaginaceae) in Sardinia. *Plant Biosystem*, *133*(1), 63-71.

Astuti, G., Brullo, S., Domina, G., El Mokni, R., Giordani, T., & Peruzzi, L. (2017). Phylogenetic relationships among tetraploid species of *Bellevalia* (Asparagaceae) endemic to south-central Mediterranean. Plant Biosystems doi: 10.1080/11263504.2017.1320308.

Bacchetta, G., Brullo, S., Velari, T. C., Chiapella, L. F., & Kosovel, V. (2012). Analysis of the *Genista ephedroides* group (Fabaceae) based on karyological, molecular and morphological data. *Caryologia*, *65*(1), 47-61.

Bacchetta, G., Coppi, A., Pontecorvo, C., & Selvi, F. (2008). Systematics, phylogenetic relationships and conservation of the taxa of *Anchusa* (Boraginaceae) endemic to Sardinia (Italy). *Systematics and Biodiversity*, *6*(2), 161-174.

Borzatti von Loewenstern, A., Giordani, T., Astuti, G., Andreucci, A., & Peruzzi, L. (2013). Phylogenetic relationships of Italian *Bellevalia* species (Asparagaceae), inferred from morphology, karyology and molecular systematics. *Plant Biosystems*, *147*(3), 776-787.

Brullo, C., Brullo, S., Downie, S. R., Danderson, C. A., & del Galdo, G. G. (2013). *Siculosciadium*, a new monotypic genus of Apiaceae from Sicily. *Annals of the Missouri Botanical Garden*, *99*(1), 1-18.

Brullo, S., Pavone, P. (1980). Chromosome numbers in the Sicilian species of "*Limonium*" Miller ("Plumbaginaceae"). *Anales del Jardín Botánico de Madrid*, 37(2), 535-555.

Calvo, J., Álvarez, I., Aedo, C., & Pelser, P. B. (2013). A phylogenetic analysis and new delimitation of *Senecio* sect. *Crociseris* (Compositae: Senecioneae), with evidence of intergeneric hybridization. *Taxon*, *62*(1), 127-140.

Carnicero, P., Saez, L., Garcia-Jacas, N., & Galbany-Casals, M. (2016). Different speciation types meet in a Mediterranean genus: the biogeographic history of *Cymbalaria* (Plantaginaceae). *bioRxiv*, 050369.

Cecchi, L., Colzi, I., Coppi, A., Gonnelli, C., & Selvi, F. (2013). Diversity and biogeography of Ni-hyperaccumulators of *Alyssum* section *Odontarrhena* (Brassicaceae) in the central western Mediterranean: evidence from karyology, morphology and DNA sequence data. *Botanical Journal of the Linnean Society*, *173*(2), 269-289.

Cecchi, L., Coppi, A., Hilger, H. H., & Selvi, F. (2014). Non-monophyly of *Buglossoides* (Boraginaceae: Lithospermeae): Phylogenetic and morphological evidence for the expansion of *Glandora* and reappraisal of *Aegonychon*. *Taxon*, *63*(5), 1065-1078.

Chacón, J., Cusimano, N., & Renner, S. S. (2014). The evolution of Colchicaceae, with a focus on chromosome numbers. *Systematic Botany*, *39*(2), 415-427.

Conti, F., Gubellini, L., Lakušić, D., & Santangelo, A. A. (2003). *Silene notarisii* (Caryophyllaceae), a neglected species of the Central Apennines, Italy. *Willdenowia*, *33*(2), 265-272.

Crema, S., Kadereit, J. W., & Cristofolini, G. (2013). Phylogenetic insights into *Primula* sect. *Auricula* in the Apennine peninsula. *Flora Mediterranea*, *23*, 157-172.

Crowl, A. A., Mavrodiev, E., Mansion, G., Haberle, R., Pistarino, A., Kamari, G., ... & Cellinese, N. (2014). Phylogeny of Campanuloideae (Campanulaceae) with emphasis on the utility of nuclear pentatricopeptide repeat (PPR) genes. *PloS one*, *9*(4), e94199.

Cubas, P., Pardo, C., Tahiri, H., & Castroviejo, S. (2010). Phylogeny and evolutionary diversification of *Adenocarpus* DC.(Leguminosae). *Taxon*, *59*(3), 720-732.

Cusma Velari, T., & Feoli Chiapella, L. (1991). Systematic relationships within the *Genista sylvestris* group (Genisteae, Fabaceae) on the basis of karyological and biometrical data. *Flora Mediterranea*, *1*, 21-29.

De Castro, O., Brullo, S., Colombo, P., Jury, S., De Luca, P., & Di Maio, A. (2012). Phylogenetic and biogeographical inferences for *Pancratium* (Amaryllidaceae), with an emphasis on the Mediterranean species based on plastid sequence data. *Botanical Journal of the Linnean Society*, *170*(1), 12-28.

De Castro, O., Cennamo, P., & De Luca, P. (2009). Analysis of the genus *Petagnaea* Caruel (Apiaceae), using new molecular and literature data. *Plant Systematics and Evolution*, *278*(3-4), 239-249.

De Castro, O., Cozzolino, S., Jury, S. L., & Caputo, P. (2002). Molecular relationships in *Genista* L. Sect. *Spartocarpus* Spach (Fabaceae). *Plant Systematics and Evolution*, *231*(1), 91-108.

De Castro, O., Innangi, M., Di Maio, A., Menale, B., Bacchetta, G., Pires, M., ... & Peruzzi, L. (2016). Disentangling phylogenetic relationships in a hotspot of diversity: the butterworts (*Pinguicula* L., Lentibulariaceae) endemic to Italy. *PloS one*, *11*(12), e0167610.

De Castro, O., Véla, E., Vendramin, G. G., Gargiulo, R., & Caputo, P. (2015). Genetic structure in the *Genista ephedroides* complex (Fabaceae) and implications for its present distribution. *Botanical Journal of the Linnean Society*, *177*(4), 607-618.

Degtjareva, G., Valiejo-Roman, C., Samigullin, T., & Sokoloff, D. (2006, June). On generic rank and phylogenetic relationships of *Dorycnopsis* Boiss. (Leguminosae, Loteae). *Anales del Jardín Botánico de Madrid* 63(1), 41-50.

Devey, D. S., Bateman, R. M., Fay, M. F., & Hawkins, J. A. (2008). Friends or relatives? Phylogenetics and species delimitation in the controversial European orchid genus *Ophrys*. *Annals of Botany*, *101*(3), 385-402.

Di Gristina, E., Geraci, A., & Raimondo, F. M. (2006). Biosystematic investigation on *Hieracium symphytifolium* (Asteraceae). *Bocconea*, *19*, 275-286.

Eldenäs, P., Anderberg, A. A., & Källersjö, M. (1998). Molecular phylogenetics of the tribeInuleae s. str. (Asteraceae), based on ITS sequences of nuclear ribosomal DNA. *Plant Systematics and Evolution*, *210*(3), 159-173.

Enke, N., & Gemeinholzer, B. (2008). Babcock revisited: new insights into generic delimitation and character evolution in *Crepis* L. (Compositae: Cichorieae) from ITS and matK sequence data. *Taxon*, *57*(3), 756-768.

Enke, N., Gemeinholzer, B., & Zidorn, C. (2012). Molecular and phytochemical systematics of the subtribe Hypochaeridinae (Asteraceae, Cichorieae). *Organisms Diversity & Evolution*, *12*(1), 1-16.

Fernández-Mazuecos, M., Blanco-Pastor, J. L., & Vargas, P. (2013). A phylogeny of toadflaxes (*Linaria* Mill.) based on nuclear internal transcribed spacer sequences: systematic and evolutionary consequences. *International Journal of Plant Sciences*, *174*(2), 234-249.

Fior, S., & Karis, P. O. (2007). Phylogeny, evolution and systematics of *Moehringia* (Caryophyllaceae) as inferred from molecular and morphological data: a case of homology reassessment. *Cladistics*, *23*(4), 362-372.

Fiz, O., Valcárcel, V., & Vargas, P. (2002). Phylogenetic position of Mediterranean Astereae and character evolution of daisies (*Bellis*, Asteraceae) inferred from nrDNA ITS sequences. *Molecular Phylogenetics and Evolution*, *25*(1), 157-171.

Fiz, O., Vargas, P., Alarcón, M. L., & Aldasoro, J. J. (2006). Phylogenetic relationships and evolution in *Erodium* (Geraniaceae) based on trnL-trnF sequences. *Systematic Botany*, *31*(4), 739-763.

Fiz-Palacios, O., Vargas, P., Vila, R., Papadopulos, A. S., & Aldasoro, J. J. (2010). The uneven phylogeny and biogeography of *Erodium* (Geraniaceae): radiations in the Mediterranean and recent recurrent intercontinental colonization. *Annals of Botany*, *106*(6), 871-884.

Fu, S., Li, L. N., Long, Z. C., Ke, W. D., Ye, A. H., Guo, Y. H., & Chen, J. M. (2016). Evaluation of the taxonomic status of water dropwort (*Oenanthe*, Apiaceae) accessions from East Asia based on nuclear rDNA internal transcribed spacer sequences. *Genetics and Molecular Research: GMR*, *15*(2).

Galbany-Casals, M., Sáez, L., & Benedí, C. (2006). Conspectus of *Helichrysum* Mill. sect. *Stoechadina* (DC.) Gren. & Godr. (Asteraceae, Gnaphalieae). *Orsis: organismes i sistemes*, *21*, 59-81.

Galbany-Casals, M., Unwin, M., Garcia-Jacas, N., Smissen, R. D., Susanna, A., & Bayer, R. J. (2014). Phylogenetic relationships in *Helichrysum* (Compositae: Gnaphalieae) and related genera: Incongruence between nuclear and plastid phylogenies, biogeographic and morphological patterns, and implications for generic delimitation. *Taxon*, *63*(3), 608-624.

Gargiulo, R., Guacchio, E. D., & Caputo, P. (2015). Phylogenetic reconstruction of *Asperula* sect. *Cynanchicae* (Rubiaceae) reveals a mosaic of evolutionary histories. *Taxon*, *64*(4), 754-769.

Gentili, R., Fenu, G., Labra, M., Bruni, I., Mattana, E., & Bacchetta, G. (2015). *Lamyropsis* genus in the Mediterranean area: phylogenetic position of *L. microcephala* (Asteraceae: Cardueae). *Plant Biosystems*, *149*(6), 944-948.

Giacalone, G. (2012). Filogenesi delle specie di *Allium* della sez. *Codonoprasum*. PhD thesis, University of Catania.

Gillespie, L. J., Soreng, R. J., Bull, R. D., Jacobs, S. W., & Refulio-Rodriguez, N. F. (2008). Phylogenetic relationships in subtribe Poinae (Poaceae, Poeae) based on nuclear ITS and plastid trn T–trn L–trn F sequences. *Botany*, *86*(8), 938-967.

Hardion, L., Dumas, P. J., Abdel-Samad, F., Kharrat, M. B. D., Surina, B., Affre, L., ... & Baumel, A. (2016). Geographical isolation caused the diversification of the Mediterranean thorny cushion-like *Astragalus* L. sect. *Tragacantha* DC.(Fabaceae). *Molecular Phylogenetics and Evolution*, *97*, 187-195.

Harpke, D., Carta, A., Tomović, G., Ranđelović, V., Ranđelović, N., Blattner, F. R., & Peruzzi, L. (2015). Phylogeny, karyotype evolution and taxonomy of *Crocus* series *Verni* (Iridaceae). *Plant Systematics and Evolution*, *301*(1), 309-325.

Hilpold, A., Vilatersana, R., Susanna, A., Meseguer, A. S., Boršić, I., Constantinidis, T., ... & Uysal, T. (2014). Phylogeny of the *Centaurea* group (Centaurea, Compositae)–geography is a better predictor than morphology. *Molecular Phylogenetics and Evolution*, *77*, 195-215.

Kirschner, J., Štepánek, J., Mes, T. H. M., Nijs, J. D., Oosterveld, P., Štorchová, H., & Kuperus, P. (2003). Principal features of the cpDNA evolution in *Taraxacum* (Asteraceae, Lactuceae): a conflict with taxonomy. *Plant Systematics and Evolution*, *239*(3), 231-255.

Kolarčik, V., Zozomová-Lihová, J., & Mártonfi, P. (2010). Systematics and evolutionary history of the *Asterotricha* group of the genus *Onosma* (Boraginaceae) in central and southern Europe inferred from AFLP and nrDNA ITS data. *Plant Systematics and Evolution*, *290*(1-4), 21-45.

Konowalik, K., Wagner, F., Tomasello, S., Vogt, R., & Oberprieler, C. (2015). Detecting reticulate relationships among diploid *Leucanthemum* Mill. (Compositae, Anthemideae) taxa using multilocus species tree reconstruction methods and AFLP fingerprinting. *Molecular Phylogenetics and Evolution*, *92*, 308-328.

Krähenbühl, M., Yuan, Y. M., & Küpfer, P. (2002). Chromosome and breeding system evolution of the genus *Mercurialis* (Euphorbiaceae): implications of ITS molecular phylogeny. *Plant Systematics and Evolution*, *234*(1-4), 155-169.

Krak, K., Caklová, P., Chrtek, J., & Fehrer, J. (2013). Reconstruction of phylogenetic relationships in a highly reticulate group with deep coalescence and recent speciation (*Hieracium*, Asteraceae). *Heredity*, *110*(2), 138-151.

Kučera, J., Marhold, K., & Lihová, J. (2010). *Cardamine maritima* group (Brassicaceae) in the amphi-Adriatic area: a hotspot of species diversity revealed by DNA sequences and morphological variation. *Taxon*, *59*(1), 148-164.

Lannér, C. (1998). Relationships of wild *Brassica* species with chromosome number 2*n* = 18, based on comparison of the DNA sequence of the chloroplast intergenic region between trnL (UAA) and trnF (GAA). *Canadian Journal of Botany*, *76*(2), 228-237.

Lega, M., Fior, S., Prosser, F., Bertolli, A., Li, M., & Varotto, C. (2012). Application of the unified species concept reveals distinct lineages for disjunct endemics of the *Brassica repanda* (Brassicaceae) complex. *Biological Journal of the Linnean Society*, *106*(3), 482-497.

Li, Y., Feng, Y., Lv, G., Liu, B., & Qi, A. (2015). The phylogeny of *Alyssum* (Brassicaceae) inferred from molecular data. *Nordic Journal of Botany*, *33*(6), 715-721.

Lledó, M. D., Crespo, M. B., Fay, M. F., & Chase, M. W. (2005). Molecular phylogenetics of *Limonium* and related genera (Plumbaginaceae): biogeographical and systematic implications. *American Journal of Botany*, *92*(7), 1189-1198.

López-Alvarado, J., Sáez, L., Filigheddu, R., Garcia-Jacas, N., & Susanna, A. (2014). The limitations of molecular markers in phylogenetic reconstruction: The case of *Centaurea* sect. *Phrygia* (Compositae). *Taxon*, *63*(5), 1079-1091.

Manning, J. C., Forest, F., Devey, D. S., Fay, M. F., & Goldblatt, P. (2009). A molecular phylogeny and a revised classification of Ornithogaloideae (Hyacinthaceae) based on an analysis of four plastid DNA regions. *Taxon*, *58*(1), 77-107.

Marhold, K., Lihova, J., Perný, M., & Bleeker, W. (2004). Comparative ITS and AFLP analysis of diploid *Cardamine* (Brassicaceae) taxa from closely related polyploid complexes. *Annals of Botany*, *93*(5), 507-520.

Marino, P., Geraci, A., & Schicchi, R. (2012). Notes on the karyology, genetics and ecology of *Genista* sect. *Voglera* in Sicily. *Plant Biosystems*, *146*(sup1), 324-329.

Moazzeni, H., Zarre, S., Pfeil, B. E., Bertrand, Y. J., German, D. A., Al-Shehbaz, I. A., ... & Oxelman, B. (2014). Phylogenetic perspectives on diversification and character evolution in the species-rich genus *Erysimum* (Erysimeae; Brassicaceae) based on a densely sampled ITS approach. *Botanical Journal of the Linnean Society*, *175*(4), 497-522.

Moraldo, B., Ricceri, C., Fiorini, G., & Demaria, G. (2011). *Viola ferrarinii* (Violaceae), a new species from the northern Apennines (Italy). *Webbia*, *66*(1), 45-55.

Nazaire, M., & Hufford, L. (2012). A broad phylogenetic analysis of Boraginaceae: implications for the relationships of *Mertensia*. *Systematic Botany*, *37*(3), 758-783.

Neves, S. S., & Watson, M. F. (2004). Phylogenetic relationships in *Bupleurum* (Apiaceae) based on nuclear ribosomal DNA ITS sequence data. *Annals of Botany*, *93*(4), 379-398.

Nürk, N. M., Madriñán, S., Carine, M. A., Chase, M. W., & Blattner, F. R. (2013). Molecular phylogenetics and morphological evolution of St. John's wort (*Hypericum*; Hypericaceae). *Molecular Phylogenetics and Evolution*, *66*(1), 1-16.

Osaloo, S. K., Maassoumi, A. A., & Murakami, N. (2003). Molecular systematics of the genus *Astragalus* L. (Fabaceae): phylogenetic analyses of nuclear ribosomal DNA internal transcribed spacers and chloroplast gene ndhF sequences. *Plant Systematics and Evolution*, *242*(1), 1-32.

Pardo, C., Cubas, P., & Tahiri, H. (2004). Molecular phylogeny and systematics of *Genista* (Leguminosae) and related genera based on nucleotide sequences of nrDNA (ITS region) and cpDNA (trnL-trnF intergenic spacer). *Plant Systematics and Evolution*, *244*(1), 93-119.

Park, J. M., Kovačić, S., Liber, Z., Eddie, W. M., & Schneeweiss, G. M. (2006). Phylogeny and biogeography of isophyllous species of *Campanula* (Campanulaceae) in the Mediterranean area. *Systematic Botany*, *31*(4), 862-880.

Paun, O., Lehnebach, C., Johansson, J. T., Lockhart, P., & Hörandl, E. (2005). Phylogenetic relationships and biogeography of *Ranunculus* and allied genera (Ranunculaceae) in the Mediterranean region and in the European alpine system. *Taxon*, *54*(4), 911-932.

Peccenini, S., & Polatschek, A. (2016). The genus *Erysimum* (Brassicaceae) in Italy, part III: key to the species and description of new species *E. apenninum*, *E. etruscum*, *E. pignattii*. *Annalen des Naturhistorischen Museums in Wien, Series B*, *118*, 147-166.

Pellicer, J., Vallès, J., Korobkov, A. A., & Garnatje, T. (2011). Phylogenetic relationships of *Artemisia* subg. *Dracunculus* (Asteraceae) based on ribosomal and chloroplast DNA sequences. *Taxon*, *60*(3), 691-704.

Perný, M., Tribsch, A., Stuessy, T. F., & Marhold, K. (2005). Allopolyploid origin of *Cardamine silana* (Brassicaceae) from Calabria (southern Italy): karyological, morphological and molecular evidence. *Botanical Journal of the Linnean Society*, *148*(1), 101-116.

Peruzzi, L. (2012). Chromosome diversity and evolution in the genus *Gagea* (Liliaceae). *Bocconea*, *24*, 147-158.

Pignatti, S. (1982). Flora d'Italia. 3 vols. *Bologna: Edagricole*.

Presti, R. M. L., Oppolzer, S., & Oberprieler, C. (2010). A molecular phylogeny and a revised classification of the Mediterranean genus *Anthemis* s.l. (Compositae, Anthemideae) based on three molecular markers and micromorphological characters. *Taxon*, *59*(5), 1441-1456.

Rešetnik, I., Frajman, B., Bogdanović, S., Ehrendorfer, F., & Schönswetter, P. (2014). Disentangling relationships among the diploid members of the intricate genus *Knautia* (Caprifoliaceae, Dipsacoideae). *Molecular Phylogenetics and Evolution*, *74*, 97-110.

Riahi, M., Zarre, S., Maassoumi, A. A., Osaloo, S. K., & Wojciechowski, M. F. (2011). Towards a phylogeny for *Astragalus* section *Caprini* (Fabaceae) and its allies based on nuclear and plastid DNA sequences. *Plant Systematics and Evolution*, *293*(1-4), 119.

Riina, R., Peirson, J. A., Geltman, D. V., Molero, J., Frajman, B., Pahlevani, A., ... & Kryukov, A. (2013). A worldwide molecular phylogeny and classification of the leafy spurges, *Euphorbia* subgenus *Esula* (Euphorbiaceae). *Taxon*, *62*(2), 316-342.

Rønsted, N., Chase, M. W., Albach, D. C., & Bello, M. A. (2002). Phylogenetic relationships within *Plantago* (Plantaginaceae): evidence from nuclear ribosomal ITS and plastid trnL-F sequence data. *Botanical Journal of the Linnean Society*, *139*(4), 323-338.

Salmaki, Y., Zarre, S., Ryding, O., Lindqvist, C., Bräuchler, C., Heubl, G., ... & Bendiksby, M. (2013). Molecular phylogeny of tribe Stachydeae (Lamiaceae subfamily Lamioideae). *Molecular Phylogenetics and Evolution*, *69*(3), 535-551.

Samuel, R., Gutermann, W., Stuessy, T. F., Ruas, C. F., Lack, H. W., Tremetsberger, K., ... & Ehrendorfer, F. (2006). Molecular phylogenetics reveals *Leontodon* (Asteraceae, Lactuceae) to be diphyletic. *American Journal of Botany*, *93*(8), 1193-1205.

Scheunert, A., & Heubl, G. (2014). Diversification of *Scrophularia* (Scrophulariaceae) in the Western Mediterranean and Macaronesia–Phylogenetic relationships, reticulate evolution and biogeographic patterns. *Molecular Phylogenetics and Evolution*, *70*, 296-313.

Sciandrello, S., Brullo, C., Brullo, S., Giusso Del Galdo, G., Minissale, P., & Salmeri, C. (2013). A new species of *Brassica* sect. *Brassica* (Brassicaceae) from Sicily. *Plant Biosystems*, *147*(3), 812-820.

Selvi, F., Coppi, A., & Bigazzi, M. (2006). Karyotype variation, evolution and phylogeny in *Borago* (Boraginaceae), with emphasis on subgenus *Buglossites* in the Corso-Sardinian system. *Annals of Botany*, *98*(4), 857-868.

Šmarda, P., Bureš, P., Horová, L., Foggi, B., & Rossi, G. (2008). Genome size and GC content evolution of *Festuca*: ancestral expansion and subsequent reduction. *Annals of botany*, *101*(3), 421-433.

Soza, V. L., & Olmstead, R. G. (2010). Molecular systematics of tribe Rubieae (Rubiaceae): Evolution of major clades, development of leaf-like whorls, and biogeography. *Taxon*, *59*(3), 755-771.

Sun, H., McLewin, W., & Fay, M. F. (2001). Molecular phylogeny of *Helleborus* (Ranunculaceae), with an emphasis on the East Asian-Mediterranean disjunction. *Taxon*, 1001-1018.

Tison, J. M., Peterson, A., Harpke, D., & Peruzzi, L. (2013). Reticulate evolution of the critical Mediterranean *Gagea* sect. *Didymobulbos* (Liliaceae) and its taxonomic implications. *Plant Systematics and Evolution*, *299*(2), 413-438.

Tiziana, C. V., Chiapella, L. F., & Kosovel, V. (2009). Karyomorphology and systematics of the eastern taxa of *Genista* sect. *Spartioides* and *G. pulchella* (Genisteae-Fabaceae). *Caryologia*, *62*(2), 102-113.

Torices, R., & Anderberg, A. A. (2009). Phylogenetic analysis of sexual systems in Inuleae (Asteraceae). *American Journal of Botany*, *96*(5), 1011-1019.

Vilatersana, R., Garcia-Jacas, N., Garnatje, T., Molero, J., Sonnante, G., & Susanna, A. (2010). Molecular phylogeny of the genus *Ptilostemon* (Compositae: Cardueae) and its relationships with *Cynara* and *Lamyropsis*. *Systematic Botany*, *35*(4), 907-917.

Vogt, R., & Oberprieler, C. (2006). The genus *Plagius* (Compositae, Anthemideae). *Willdenowia*, *36*(1), 47-68.

Walker, J. B., Sytsma, K. J., Treutlein, J., & Wink, M. (2004). *Salvia* (Lamiaceae) is not monophyletic: implications for the systematics, radiation, and ecological specializations of *Salvia* and tribe Mentheae. *American Journal of Botany*, *91*(7), 1115-1125.

Wanke, S. (2006). Evolution of the genus *Aristolochia*-Systematics, Molecular Evolution and Ecology. PhD thesis, University of Dresden.

Warwick, S. I., & Sauder, C. A. (2005). Phylogeny of tribe Brassiceae (Brassicaceae) based on chloroplast restriction site polymorphisms and nuclear ribosomal internal transcribed spacer and chloroplast trn L intron sequences. *Canadian Journal of Botany*, *83*(5), 467-483.

Weitzel, C., Rønsted, N., Spalik, K., & Simonsen, H. T. (2014). Resurrecting deadly carrots: towards a revision of *Thapsia* (Apiaceae) based on phylogenetic analysis of nrITS sequences and chemical profiles. *Botanical Journal of the Linnean Society*, *174*(4), 620-636.

Yockteng, R., Jr Ballard, H. E., Mansion, G., Dajoz, I., & Nadot, S. (2003). Relationships among pansies (*Viola* section *Melanium*) investigated using ITS and ISSR markers. *Plant Systematics and Evolution*, *241*(3), 153-170.

Table S2. List of species including number of samples, mean chromosome number, categorical predictor variables and climate predictor variables (bio1 = mean annual temperature, bio4 = temperature seasonality, bio7 = temperature continentality, bio12 = mean annual precipitation, bio15 = precipitation seasonality). *the status of this taxon was recently changed and included in *Rhaponticoides centaurium*.

clade	taxon	2*n*	*x*	sample	Life form	Flower type	Inflorescence	bio1	bio4	bio7	bio12	bio15	Habitat light	Habitat moisture	Habitat nutrient
Malvids	*Acer cappadocicum* subsp. *lobelii*	26.0	13.0	1	woody	small	single	15.00	545.20	21.40	892.00	49.00	shade	moist	mesotrophic
Campanulids	*Achillea barrelieri* subsp. *barrelieri*	18.0	9.0	1	perennial	small	inflorescence	7.20	619.80	24.30	855.00	22.00	open	dry	oligotrophic
Campanulids	*Achillea barrelieri* subsp. *mucronulata*	18.0	9.0	1	perennial	small	inflorescence	11.10	577.40	22.10	854.00	41.00	open	dry	oligotrophic
Campanulids	*Achillea rupestris*	18.0	9.0	1	perennial	small	inflorescence	11.10	577.40	22.10	854.00	41.00	open	dry	oligotrophic
Monocots	*Acis rosea*	16.0	8.0	2	geophyte	large	single	15.60	487.45	20.70	650.50	53.00	open	dry	oligotrophic
Fabids	*Adenocarpus complicatus* subsp. *bivonae*	52.0	13.0	1	woody	small	single	11.30	597.80	23.30	672.00	49.00	shade	moist	mesotrophic
Fabids	*Adenocarpus complicatus* subsp. *brutius*	52.0	13.0	1	woody	small	single	11.60	569.00	22.50	829.00	51.00	open	dry	oligotrophic
Fabids	*Adenocarpus complicatus* subsp. *commutatus*	52.0	13.0	1	woody	small	single	15.30	556.50	22.30	748.00	53.00	open	dry	oligotrophic
Fabids	*Adenocarpus complicatus* subsp. *samniticus*	52.0	13.0	2	woody	small	single	11.10	619.60	25.20	835.00	27.00	semi	moist	mesotrophic
Campanulids	*Adoxa moschatellina* subsp. *cescae*	54.0	18.0	1	perennial	small	single	13.70	545.60	21.50	918.00	49.00	shade	moist	eutrophic
Lamiids	*Aegonychon calabrum*	20.0	10.0	1	perennial	large	single	14.10	542.30	21.20	911.00	47.00	semi	moist	oligotrophic
Monocots	*Agrostis canina* subsp. *montelucii*	14.0	7.0	3	perennial	incospicious	inflorescence	14.07	605.73	26.03	752.67	31.67	open	wet	oligotrophic
Lamiids	*Ajuga tenorei*	32.0	8.0	2	perennial	large	single	9.35	613.80	23.80	731.50	35.00	semi	dry	oligotrophic
Monocots	*Allium aetnense*	16.0	8.0	1	geophyte	large	single	10.10	612.00	23.50	654.00	47.00	semi	dry	mesotrophic
Monocots	*Allium agrigentinum*	16.0	8.0	1	geophyte	large	single	16.60	524.30	21.00	471.00	61.00	open	dry	oligotrophic
Monocots	*Allium anzalonei*	32.0	8.0	1	geophyte	large	single	14.80	624.80	27.80	830.00	31.00	semi	moist	mesotrophic
Monocots	*Allium apulum*	16.0	8.0	1	geophyte	large	single	16.70	570.50	24.40	570.00	46.00	open	dry	mesotrophic
Monocots	*Allium calabrum*	16.0	8.0	1	geophyte	large	single	13.90	548.30	21.40	914.00	48.00	open	dry	mesotrophic
Monocots	*Allium castellanense*	24.0	8.0	1	geophyte	large	single	14.30	584.20	22.80	499.00	55.00	open	dry	oligotrophic
Monocots	*Allium cupanii*	32.0	8.0	1	geophyte	large	single	14.80	571.00	22.70	557.00	56.00	semi	dry	mesotrophic
Monocots	*Allium diomedeum*	16.0	8.0	1	geophyte	large	single	16.10	583.10	23.10	561.00	31.00	open	dry	oligotrophic
Monocots	*Allium franciniae*	32.0	8.0	1	geophyte	large	single	17.80	485.20	21.40	463.00	62.00	open	dry	oligotrophic
Monocots	*Allium garbarii*	16.0	8.0	1	geophyte	large	single	17.70	570.70	24.50	666.00	65.00	open	dry	oligotrophic
Monocots	*Allium garganicum*	16.0	8.0	1	geophyte	large	single	15.30	573.70	22.10	492.00	35.00	open	dry	mesotrophic
Monocots	*Allium hemisphaericum*	32.0	8.0	1	geophyte	large	single	18.70	456.50	17.10	302.00	73.00	semi	dry	oligotrophic

clade	taxon	2*n*	*x*	sample	Life form	Flower type	Inflorescence	bio1	bio4	bio7	bio12	bio15	Habitat light	Habitat moisture	Habitat nutrient
Monocots	*Allium insubricum*	14.0	7.0	1	geophyte	large	single	8.40	639.60	26.30	1162.0	29.00	open	dry	oligotrophic
Monocots	*Allium lehmannii*	16.0	8.0	1	geophyte	large	single	18.00	510.10	20.30	593.00	61.00	open	dry	oligotrophic
Monocots	*Allium lopadusanum*	16.0	8.0	1	geophyte	large	single	18.70	456.50	17.10	302.00	73.00	open	dry	oligotrophic
Monocots	*Allium nebrodense*	16.0	8.0	1	geophyte	large	single	13.50	591.00	22.90	536.00	52.00	open	dry	oligotrophic
Monocots	*Allium obtusiflorum*	16.0	8.0	2	geophyte	large	single	17.65	512.45	21.80	534.00	69.00	open	dry	oligotrophic
Monocots	*Allium panormitanum*	32.0	8.0	1	geophyte	large	single	17.60	516.90	20.70	591.00	60.00	semi	dry	mesotrophic
Monocots	*Allium parciflorum*	16.0	8.0	3	geophyte	large	single	14.03	521.43	21.50	692.33	53.67	open	dry	oligotrophic
Monocots	*Allium pelagicum*	14.0	7.0	1	geophyte	large	single	18.70	456.50	17.10	302.00	73.00	open	dry	mesotrophic
Monocots	*Allium pentadactyli*	16.0	8.0	1	geophyte	large	single	16.50	539.80	22.50	789.00	57.00	open	dry	mesotrophic
Monocots	*Allium samniticum*	32.0	8.0	1	geophyte	large	single	10.40	627.50	26.70	871.00	26.00	semi	dry	mesotrophic
Monocots	*Allium vernale*	28.0	7.0	1	geophyte	large	single	17.60	543.90	25.40	540.00	69.00	semi	dry	mesotrophic
Malvids	*Alyssum diffusum* subsp. *calabricum*	32.0	8.0	1	perennial	small	single	13.70	545.60	21.50	918.00	49.00	open	dry	oligotrophic
Malvids	*Alyssum diffusum* subsp. *diffusum*	16.0	8.0	1	perennial	small	single	12.60	602.60	23.10	571.00	23.00	open	dry	oligotrophic
Malvids	*Alyssum diffusum* subsp. *garganicum*	16.0	8.0	1	perennial	small	single	12.60	602.60	23.10	571.00	23.00	open	dry	mesotrophic
Lamiids	*Anarrhinum corsicum*	18.0	9.0	1	perennial	large	single	8.50	531.60	19.90	869.00	35.00	open	dry	oligotrophic
Lamiids	*Anchusa capellii*	16.0	8.0	1	perennial	large	single	13.60	574.60	24.20	652.00	50.00	semi	dry	mesotrophic
Lamiids	*Anchusa crispa* subsp. *crispa*	16.0	8.0	1	perennial	large	single	16.10	487.70	21.40	589.00	63.00	open	dry	oligotrophic
Lamiids	*Anchusa crispa* subsp. *maritima*	16.0	8.0	1	perennial	large	single	15.30	526.50	22.00	633.00	55.00	open	dry	oligotrophic
Lamiids	*Anchusa formosa*	16.0	8.0	1	perennial	large	single	15.00	538.70	23.60	583.00	55.00	semi	dry	mesotrophic
Lamiids	*Anchusa litorea*	16.0	8.0	1	perennial	large	single	16.60	511.70	22.30	553.00	58.00	open	dry	oligotrophic
Lamiids	*Anchusa montelinasana*	16.0	8.0	1	perennial	large	single	14.10	545.60	23.50	737.00	56.00	open	dry	mesotrophic
Lamiids	*Anchusa sardoa*	16.0	8.0	1	perennial	large	single	16.10	487.70	21.40	589.00	63.00	open	dry	mesotrophic
Campanulids	*Andryala tenuifolia*	18.0	9.0	1	annual	small	inflorescence	15.60	546.40	23.10	459.00	63.00	open	dry	oligotrophic
Campanulids	*Anthemis aetnensis*	27.0	9.0	2	perennial	small	inflorescence	11.30	597.80	23.30	672.00	49.00	open	dry	oligotrophic
Campanulids	*Anthemis arvensis* subsp. *sphacelata*	18.0	9.0	1	annual	small	inflorescence	11.50	607.80	23.30	608.00	48.00	open	moist	mesotrophic
Campanulids	*Anthemis cretica* subsp. *calabrica*	36.0	9.0	1	perennial	small	inflorescence	12.70	555.30	22.30	918.00	53.00	open	dry	oligotrophic
Campanulids	*Anthemis cretica* subsp. *messanensis*	36.0	9.0	1	perennial	small	inflorescence	15.30	556.50	22.30	748.00	53.00	open	dry	oligotrophic
Campanulids	*Anthemis cupaniana*	36.0	9.0	2	perennial	small	inflorescence	14.75	573.10	22.70	513.00	55.50	open	moist	oligotrophic
Campanulids	*Anthemis hydruntina* subsp. *hydruntina*	18.0	9.0	1	annual	small	inflorescence	16.40	537.80	21.60	688.00	51.00	open	dry	oligotrophic

clade	taxon	2*n*	*x*	sample	Life form	Flower type	Inflorescence	bio1	bio4	bio7	bio12	bio15	Habitat light	Habitat moisture	Habitat nutrient
Campanulids	*Anthemis ismelia*	18.0	9.0	1	annual	small	inflorescence	18.00	510.10	20.30	593.00	61.00	open	dry	oligotrophic
Campanulids	*Anthemis muricata*	18.0	9.0	1	perennial	small	inflorescence	14.70	575.20	22.70	469.00	55.00	open	dry	oligotrophic
Fabids	*Anthyllis hermanniae* subsp. *brutia*	14.0	7.0	1	woody	small	single	17.20	567.50	24.50	700.00	64.00	open	dry	oligotrophic
Fabids	*Anthyllis hermanniae* subsp. *corsica*	14.0	7.0	1	woody	small	single	10.60	520.20	20.30	810.00	38.00	open	dry	oligotrophic
Fabids	*Anthyllis hermanniae* subsp. *ichnusae*	14.0	7.0	1	woody	small	single	14.90	545.80	22.50	594.00	51.00	open	dry	oligotrophic
Fabids	*Anthyllis hermanniae* subsp. *sicula*	14.0	7.0	1	woody	small	single	17.20	471.30	19.40	396.00	64.00	open	dry	oligotrophic
Malvids	*Arabis collina* subsp. *rosea*	32.0	8.0	1	perennial	large	single	11.60	584.60	22.80	720.00	37.00	semi	moist	oligotrophic
Caryophyllales	*Arenaria bertolonii*	30.0	15.0	1	perennial	small	single	8.60	624.60	23.80	899.00	22.00	open	dry	oligotrophic
Caryophyllales	*Arenaria huteri*	88.0	22.0	1	perennial	small	single	5.20	664.90	29.00	972.00	35.00	semi	moist	oligotrophic
Caryophyllales	*Armeria brutia*	18.0	9.0	1	perennial	small	single	11.10	573.70	22.00	880.00	46.00	open	dry	oligotrophic
Caryophyllales	*Armeria denticulata*	18.0	9.0	1	perennial	small	single	14.10	654.80	28.30	860.00	25.00	open	dry	oligotrophic
Caryophyllales	*Armeria gracilis* subsp. *gracilis*	18.0	9.0	1	perennial	small	single	11.10	577.40	22.10	854.00	41.00	open	dry	oligotrophic
Caryophyllales	*Armeria gracilis* subsp. *majellensis*	18.0	9.0	1	perennial	small	single	7.20	619.80	24.30	855.00	22.00	open	dry	oligotrophic
Caryophyllales	*Armeria gussonei*	18.0	9.0	1	perennial	small	single	14.80	571.00	22.70	557.00	56.00	open	dry	oligotrophic
Caryophyllales	*Armeria morisii*	18.0	9.0	1	perennial	small	single	13.90	560.30	23.10	684.00	51.00	open	dry	oligotrophic
Caryophyllales	*Armeria nebrodensis*	18.0	9.0	1	perennial	small	single	13.50	591.00	22.90	536.00	52.00	open	dry	oligotrophic
Caryophyllales	*Armeria sardoa* subsp. *genargentea*	18.0	9.0	1	perennial	small	single	11.20	592.40	24.00	890.00	50.00	open	dry	oligotrophic
Caryophyllales	*Armeria sardoa* subsp. *sardoa*	18.0	9.0	2	perennial	small	single	14.00	551.70	22.95	636.00	50.50	open	dry	oligotrophic
Caryophyllales	*Armeria saviana*	18.0	9.0	1	perennial	small	single	12.60	606.40	24.80	620.00	28.00	open	dry	oligotrophic
Caryophyllales	*Armeria sulcitana*	18.0	9.0	1	perennial	small	single	15.00	538.70	23.60	583.00	55.00	open	dry	oligotrophic
Campanulids	*Artemisia caerulescens* subsp. *cretacea*	18.0	9.0	1	perennial	small	inflorescence	12.70	609.50	26.40	726.00	25.00	open	moist	mesotrophic
Campanulids	*Artemisia campestris* subsp. *variabilis*	54.0	9.0	2	perennial	small	inflorescence	16.25	541.50	22.60	768.50	56.50	open	dry	mesotrophic
Monocots	*Arum apulum*	56.0	14.0	1	geophyte	small	single	14.40	584.40	24.80	582.00	31.00	shade	moist	mesotrophic
Lamiids	*Asperula apuana*	44.0	11.0	1	perennial	large	single	9.80	610.40	23.60	895.00	26.00	open	dry	oligotrophic
Lamiids	*Asperula aristata* subsp. *calabra*	40.0	10.0	1	perennial	large	single	9.80	584.80	22.20	845.00	40.00	open	dry	oligotrophic
Lamiids	*Asperula deficiens*	20.0	10.0	1	perennial	large	single	16.20	506.00	21.90	518.00	52.00	open	dry	oligotrophic
Lamiids	*Asperula lactea*	40.0	10.0	1	perennial	large	single	11.60	569.00	22.50	829.00	51.00	open	dry	oligotrophic
Lamiids	*Asperula rupestris*	44.0	11.0	1	perennial	large	single	17.70	519.60	21.10	572.00	61.00	open	dry	oligotrophic
Lamiids	*Asperula staliana* subsp. *diomedea*	40.0	10.0	1	perennial	large	single	16.10	583.10	23.10	561.00	31.00	open	dry	oligotrophic

clade	taxon	2*n*	*x*	sample	Life form	Flower type	Inflorescence	bio1	bio4	bio7	bio12	bio15	Habitat light	Habitat moisture	Habitat nutrient
Fabids	*Astragalus aquilanus*	16.0	8.0	1	perennial	small	single	7.60	616.70	24.60	842.00	23.00	open	dry	oligotrophic
Fabids	*Astragalus caprinus* subsp. *huetii*	16.0	8.0	1	perennial	small	single	17.60	493.00	21.40	469.00	61.00	open	dry	oligotrophic
Fabids	*Astragalus genargenteus*	16.0	8.0	1	perennial	small	single	11.20	592.40	24.00	890.00	50.00	open	dry	oligotrophic
Fabids	*Astragalus maritimus*	16.0	8.0	1	perennial	small	single	16.50	499.70	21.80	646.00	63.00	open	dry	oligotrophic
Fabids	*Astragalus muelleri* subsp. *etruscus*	16.0	8.0	1	perennial	small	single	15.00	558.30	25.20	634.00	39.00	open	dry	oligotrophic
Fabids	*Astragalus nebrodensis*	16.0	8.0	1	woody	small	single	13.50	591.00	22.90	536.00	52.00	open	dry	oligotrophic
Fabids	*Astragalus parnassi* subsp. *calabricus*	16.0	8.0	1	woody	small	single	10.20	578.10	22.10	860.00	45.00	open	dry	oligotrophic
Fabids	*Astragalus raphaelis*	16.0	8.0	1	annual	small	single	15.40	544.30	21.60	456.00	58.00	open	dry	oligotrophic
Fabids	*Astragalus siculus*	32.0	8.0	1	woody	small	single	11.30	597.80	23.30	672.00	49.00	open	dry	oligotrophic
Fabids	*Astragalus sirinicus*	16.0	8.0	1	woody	small	single	11.20	592.40	24.00	890.00	50.00	open	dry	oligotrophic
Fabids	*Astragalus verrucosus*	16.0	8.0	1	perennial	small	single	16.50	503.80	21.60	578.00	59.00	open	dry	oligotrophic
Campanulids	*Astrantia pauciflora* subsp. *pauciflora*	16.0	8.0	1	perennial	small	inflorescence	9.80	610.40	23.60	895.00	26.00	open	moist	oligotrophic
Campanulids	*Astrantia pauciflora* subsp. *tenorei*	21.0	7.0	1	perennial	small	inflorescence	7.20	619.80	24.30	855.00	22.00	open	moist	oligotrophic
Campanulids	*Athamanta cortiana*	22.0	11.0	1	perennial	small	inflorescence	9.80	610.40	23.60	895.00	26.00	open	dry	oligotrophic
Malvids	*Aubrieta deltoidea* subsp. *sicula*	16.0	8.0	1	perennial	large	single	13.50	591.00	22.90	536.00	52.00	open	dry	oligotrophic
Malvids	*Barbarea rupicola*	16.0	8.0	2	perennial	small	single	11.25	523.00	20.75	802.00	38.50	open	dry	oligotrophic
Monocots	*Bellardiochloa variegata* subsp. *aetnensis*	14.0	7.0	1	perennial	incospicious	inflorescence	11.30	597.80	23.30	672.00	49.00	open	moist	oligotrophic
Monocots	*Bellevalia dubia*	8.0	4.0	1	geophyte	large	single	17.80	485.20	21.40	463.00	62.00	open	moist	mesotrophic
Monocots	*Bellevalia pelagica*	16.0	4.0	1	geophyte	large	single	18.70	456.50	17.10	302.00	73.00	open	dry	oligotrophic
Monocots	*Bellevalia webbiana*	16.0	4.0	1	geophyte	large	single	14.10	654.80	28.30	860.00	25.00	semi	moist	mesotrophic
Campanulids	*Bellis margaritaefolia*	18.0	9.0	1	perennial	small	inflorescence	11.10	573.70	22.00	880.00	46.00	open	dry	oligotrophic
Campanulids	*Bellium crassifolium*	18.0	9.0	1	perennial	small	inflorescence	16.50	499.70	21.80	646.00	63.00	open	dry	oligotrophic
Lamiids	*Betonica alopecuros* subsp. *divulsa*	16.0	8.0	1	perennial	large	single	7.20	619.80	24.30	855.00	22.00	open	moist	oligotrophic
Malvids	*Biscutella apuana*	18.0	9.0	1	perennial	large	single	9.80	610.40	23.60	895.00	26.00	open	dry	oligotrophic
Malvids	*Biscutella incana*	18.0	9.0	1	perennial	large	single	11.10	577.40	22.10	854.00	41.00	open	dry	oligotrophic
Malvids	*Biscutella laevigata* subsp. *australis*	36.0	9.0	1	perennial	large	single	8.20	619.00	24.70	827.00	23.00	open	dry	oligotrophic
Malvids	*Biscutella laevigata* subsp. *lucia*	36.0	9.0	1	perennial	large	single	10.00	706.80	29.60	824.00	26.00	open	dry	oligotrophic
Malvids	*Biscutella laevigata* subsp. *raffaelliana*	36.0	9.0	1	perennial	large	single	11.50	637.30	24.90	1227.0	17.00	open	dry	oligotrophic
Malvids	*Biscutella maritima*	16.0	8.0	1	perennial	large	single	13.60	578.10	26.20	978.00	43.00	open	dry	oligotrophic

clade	taxon	2*n*	*x*	sample	Life form	Flower type	Inflorescence	bio1	bio4	bio7	bio12	bio15	Habitat light	Habitat moisture	Habitat nutrient
Malvids	*Biscutella mollis*	18.0	9.0	1	perennial	large	single	14.90	557.00	24.40	593.00	40.00	open	dry	oligotrophic
Malvids	*Biscutella pichiana* subsp. *ilvensis*	18.0	9.0	1	perennial	large	single	14.50	526.40	20.30	609.00	40.00	open	dry	oligotrophic
Malvids	*Biscutella pichiana* subsp. *pichiana*	18.0	9.0	2	perennial	large	single	14.50	571.60	25.20	736.50	35.50	open	dry	oligotrophic
Malvids	*Biscutella prealpina*	18.0	9.0	1	perennial	large	single	7.10	661.50	26.90	796.00	31.00	open	dry	oligotrophic
Fabids	*Bituminaria morisiana*	20.0	10.0	1	woody	small	single	15.30	516.90	21.80	581.00	51.00	open	dry	mesotrophic
Lamiids	*Borago morisiana*	18.0	9.0	3	perennial	large	single	15.53	524.93	22.63	678.33	59.67	shade	moist	oligotrophic
Lamiids	*Borago pygmaea*	38.3	10.0	7	perennial	large	single	13.93	528.66	21.84	639.57	45.29	shade	wet	mesotrophic
Monocots	*Brachypodium genuense*	18.0	9.0	2	perennial	incospicious	inflorescence	9.35	635.85	25.75	887.00	23.00	open	moist	oligotrophic
Malvids	*Brassica glabrescens*	20.0	10.0	1	perennial	large	single	12.30	683.20	29.50	1341.0	21.00	open	dry	oligotrophic
Malvids	*Brassica macrocarpa*	18.0	9.0	1	perennial	large	single	17.80	485.20	21.40	463.00	62.00	open	dry	oligotrophic
Malvids	*Brassica raimondoi*	18.0	9.0	1	perennial	large	single	15.30	562.80	22.90	681.00	55.00	open	dry	oligotrophic
Malvids	*Brassica rupestris*	18.0	9.0	1	perennial	large	single	14.80	571.00	22.70	557.00	56.00	open	dry	oligotrophic
Malvids	*Brassica tyrrhena*	18.0	9.0	1	perennial	large	single	14.90	545.80	22.50	594.00	51.00	open	dry	oligotrophic
Malvids	*Brassica villosa* subsp. *bivonana*	18.0	9.0	1	perennial	large	single	16.90	525.50	21.60	504.00	60.00	open	dry	oligotrophic
Malvids	*Brassica villosa* subsp. *drepanensis*	18.0	9.0	1	perennial	large	single	17.40	501.60	21.00	506.00	60.00	open	dry	oligotrophic
Malvids	*Brassica villosa* subsp. *tinei*	18.0	9.0	1	perennial	large	single	16.60	524.30	21.00	471.00	61.00	open	dry	oligotrophic
Malvids	*Brassica villosa* subsp. *villosa*	18.0	9.0	1	perennial	large	single	15.60	551.90	21.80	582.00	57.00	open	dry	oligotrophic
Fabids	*Bryonia marmorata*	40.0	20.0	1	perennial	small	single	14.10	506.60	20.80	697.00	46.00	shade	dry	mesotrophic
Campanulids	*Bunium corydalinum*	22.0	11.0	1	perennial	small	inflorescence	8.50	531.60	19.90	869.00	35.00	open	dry	oligotrophic
Campanulids	*Buphthalmum inuloides*	20.0	10.0	1	woody	small	inflorescence	15.90	489.90	19.90	539.00	52.00	open	dry	oligotrophic
Campanulids	*Buphthalmum salicifolium* subsp. *flexile*	22.0	11.0	1	perennial	small	inflorescence	9.80	610.40	23.60	895.00	26.00	open	moist	mesotrophic
Campanulids	*Bupleurum dianthifolium*	32.0	16.0	2	perennial	small	inflorescence	17.00	509.80	21.90	425.00	58.00	open	dry	oligotrophic
Campanulids	*Bupleurum falcatum* subsp. *corsicum*	16.0	8.0	1	perennial	small	inflorescence	8.50	531.60	19.90	869.00	35.00	open	dry	oligotrophic
Monocots	*Calamagrostis corsica*	28.0	7.0	1	perennial	incospicious	inflorescence	9.10	531.90	20.10	843.00	37.00	semi	moist	oligotrophic
Campanulids	*Calendula maritima*	32.0	8.0	1	woody	small	inflorescence	17.40	501.60	21.00	506.00	60.00	open	dry	oligotrophic
Campanulids	*Campanula bertolae*	102.0	37.0	1	perennial	large	single	8.50	645.70	27.10	936.00	19.00	open	dry	oligotrophic
Campanulids	*Campanula carnica* subsp. *puberula*	34.0	17.0	1	perennial	large	single	10.50	706.10	29.70	909.00	25.00	open	moist	oligotrophic
Campanulids	*Campanula elatines*	34.0	17.0	1	perennial	large	single	3.40	577.20	23.60	1285.0	10.00	open	moist	oligotrophic
Campanulids	*Campanula elatinoides*	34.5	17.0	2	perennial	large	single	10.50	706.10	29.70	909.00	25.00	open	moist	oligotrophic

clade	taxon	2*n*	*x*	sample	Life form	Flower type	Inflorescence	bio1	bio4	bio7	bio12	bio15	Habitat light	Habitat moisture	Habitat nutrient
Campanulids	*Campanula forsythii*	34.0	17.0	1	perennial	large	single	16.20	506.00	21.90	518.00	52.00	open	dry	oligotrophic
Campanulids	*Campanula fragilis* subsp. *cavolinii*	32.0	16.0	1	perennial	large	single	12.40	611.10	25.50	784.00	25.00	open	dry	oligotrophic
Campanulids	*Campanula fragilis* subsp. *fragilis*	32.0	16.0	1	perennial	large	single	14.10	542.30	21.20	911.00	47.00	open	dry	oligotrophic
Campanulids	*Campanula garganica* subsp. *garganica*	34.0	17.0	1	perennial	large	single	12.60	602.60	23.10	571.00	23.00	open	dry	oligotrophic
Campanulids	*Campanula isophylla*	32.0	16.0	1	perennial	large	single	13.50	575.50	24.10	877.00	32.00	open	dry	oligotrophic
Campanulids	*Campanula martinii*	102.0	37.0	1	perennial	large	single	10.30	706.30	29.30	826.00	25.00	open	moist	oligotrophic
Campanulids	*Campanula reatina*	34.0	17.0	1	perennial	large	single	10.80	636.80	27.20	848.00	24.00	open	dry	oligotrophic
Campanulids	*Campanula sabatia*	34.0	17.0	1	perennial	large	single	13.50	575.50	24.10	877.00	32.00	open	dry	oligotrophic
Campanulids	*Campanula scheuchzeri* subsp. *pollinensis*	34.0	17.0	1	perennial	large	single	11.10	577.40	22.10	854.00	41.00	open	moist	oligotrophic
Campanulids	*Campanula scheuchzeri* subsp. *pseudostenocodon*	102.0	37.0	1	perennial	large	single	6.80	613.90	23.60	837.00	20.00	open	moist	oligotrophic
Campanulids	*Campanula tanfanii*	34.0	17.0	1	perennial	large	single	7.90	623.30	23.90	877.00	22.00	open	moist	oligotrophic
Malvids	*Cardamine apennina*	16.0	8.0	2	perennial	large	single	11.85	625.95	26.65	861.50	26.00	semi	wet	mesotrophic
Malvids	*Cardamine battagliae*	160.0	8.0	1	perennial	large	single	13.90	548.30	21.40	914.00	48.00	shade	moist	mesotrophic
Malvids	*Cardamine granulosa*	16.0	8.0	1	perennial	large	single	11.10	689.70	29.00	835.00	25.00	semi	moist	mesotrophic
Malvids	*Cardamine monteluccii*	16.0	8.0	2	annual	large	single	12.35	625.85	25.30	770.00	23.00	semi	moist	eutrophic
Malvids	*Cardamine silana*	48.0	8.0	1	perennial	large	single	10.20	578.10	22.10	860.00	45.00	semi	wet	eutrophic
Campanulids	*Carduus affinis* subsp. *affinis*	22.0	11.0	3	perennial	small	inflorescence	9.27	596.53	22.93	844.67	34.00	open	dry	eutrophic
Campanulids	*Carduus affinis* subsp. *brutius*	22.0	11.0	1	perennial	small	inflorescence	11.70	569.00	21.80	873.00	43.00	open	dry	eutrophic
Campanulids	*Carduus corymbosus*	26.0	13.0	2	annual	small	inflorescence	16.20	551.05	23.45	522.00	63.00	open	dry	eutrophic
Campanulids	*Carduus fasciculiflorus*	22.0	11.0	1	perennial	small	inflorescence	13.90	504.70	20.20	660.00	47.00	open	dry	eutrophic
Campanulids	*Carduus nutans* subsp. *siculus*	16.0	8.0	1	perennial	small	inflorescence	14.80	571.00	22.70	557.00	56.00	open	dry	mesotrophic
Monocots	*Carex ferruginea*	40.0	20.0	1	perennial	incospicious	inflorescence	9.80	610.40	23.60	895.00	26.00	open	moist	mesotrophic
Monocots	*Carex microcarpa*	60.0	30.0	1	geophyte	incospicious	inflorescence	13.90	504.70	20.20	660.00	47.00	shade	wet	mesotrophic
Campanulids	*Carlina nebrodensis*	20.0	10.0	1	perennial	small	inflorescence	13.50	591.00	22.90	536.00	52.00	open	dry	oligotrophic
Campanulids	*Carlina sicula*	18.0	9.0	2	perennial	small	inflorescence	16.60	531.65	21.90	525.00	61.50	open	dry	oligotrophic
Campanulids	*Carum appuanum* subsp. *appuanum*	22.0	11.0	1	perennial	small	inflorescence	9.80	610.40	23.60	895.00	26.00	open	dry	mesotrophic
Campanulids	*Castroviejoa frigida*	28.0	14.0	1	woody	small	inflorescence	9.10	531.90	20.10	843.00	37.00	open	dry	oligotrophic
Campanulids	*Castroviejoa montelinasana*	28.0	14.0	1	woody	small	inflorescence	14.10	545.60	23.50	737.00	56.00	open	dry	oligotrophic
Campanulids	*Centaurea aeolica* subsp. *aeolica*	18.0	9.0	1	perennial	small	inflorescence	17.20	527.70	21.50	649.00	54.00	open	dry	oligotrophic

clade	taxon	2*n*	*x*	sample	Life form	Flower type	Inflorescence	bio1	bio4	bio7	bio12	bio15	Habitat light	Habitat moisture	Habitat nutrient
Campanulids	*Centaurea aetaliae*	18.0	9.0	1	perennial	small	inflorescence	15.50	533.30	21.30	593.00	41.00	open	dry	oligotrophic
Campanulids	*Centaurea ambigua* subsp. *nigra*	18.0	9.0	1	perennial	small	inflorescence	7.20	619.80	24.30	855.00	22.00	open	moist	oligotrophic
Campanulids	*Centaurea aplolepa* subsp. *aplolepa*	18.0	9.0	1	perennial	small	inflorescence	14.40	572.00	24.00	959.00	35.00	open	dry	oligotrophic
Campanulids	*Centaurea aplolepa* subsp. *carueliana*	18.0	9.0	1	perennial	small	inflorescence	13.10	654.50	27.40	888.00	25.00	open	dry	oligotrophic
Campanulids	*Centaurea aplolepa* subsp. *cosana*	18.0	9.0	1	perennial	small	inflorescence	14.90	557.00	24.40	593.00	40.00	open	dry	oligotrophic
Campanulids	*Centaurea aplolepa* subsp. *lunensis*	18.0	9.0	1	perennial	small	inflorescence	14.40	572.00	24.00	959.00	35.00	open	dry	oligotrophic
Campanulids	*Centaurea aplolepa* subsp. *maremmana*	18.0	9.0	1	perennial	small	inflorescence	14.70	567.80	24.60	808.00	35.00	open	dry	oligotrophic
Campanulids	*Centaurea aplolepa* subsp. *subciliata*	18.0	9.0	2	perennial	small	inflorescence	15.10	555.00	23.75	761.50	38.00	open	dry	oligotrophic
Campanulids	*Centaurea arachnoidea* subsp. *arachnoidea*	20.0	10.0	1	perennial	small	inflorescence	12.70	590.30	24.30	934.00	31.00	open	dry	oligotrophic
Campanulids	*Centaurea arachnoidea* subsp. *montis-ferrati*	20.0	10.0	1	perennial	small	inflorescence	13.10	654.50	27.40	888.00	25.00	open	dry	oligotrophic
Campanulids	*Centaurea arrigoni*	18.0	9.0	1	perennial	small	inflorescence	8.50	616.50	23.70	891.00	23.00	open	dry	oligotrophic
Campanulids	*Centaurea busambarensis*	18.0	9.0	1	perennial	small	inflorescence	14.80	571.00	22.70	557.00	56.00	open	dry	oligotrophic
Campanulids	*Centaurea ceratophylla* subsp. *ceratophylla*	21.5	10.8	4	perennial	small	inflorescence	11.70	605.68	25.25	891.25	31.50	open	dry	oligotrophic
Campanulids	*Centaurea cineraria* subsp. *cineraria*	18.0	9.0	1	perennial	small	inflorescence	15.00	544.20	21.80	826.00	46.00	open	dry	oligotrophic
Campanulids	*Centaurea cineraria* subsp. *circae*	18.0	9.0	1	perennial	small	inflorescence	15.50	518.70	23.80	895.00	51.00	open	dry	oligotrophic
Campanulids	*Centaurea corensis*	36.0	9.0	2	perennial	small	inflorescence	15.90	533.90	23.45	734.50	54.00	open	dry	oligotrophic
Campanulids	*Centaurea diomedea*	18.0	9.0	1	perennial	small	inflorescence	16.10	583.10	23.10	561.00	31.00	open	dry	oligotrophic
Campanulids	*Centaurea erycina*	18.0	9.0	1	perennial	small	inflorescence	17.40	501.60	21.00	506.00	60.00	open	dry	oligotrophic
Campanulids	*Centaurea filiformis* subsp. *ferulacea*	18.0	9.0	1	perennial	small	inflorescence	14.40	551.50	22.40	578.00	49.00	open	dry	oligotrophic
Campanulids	*Centaurea filiformis* subsp. *filiformis*	18.0	9.0	1	perennial	small	inflorescence	14.90	545.80	22.50	594.00	51.00	open	dry	oligotrophic
Campanulids	*Centaurea giardinae*	18.0	9.0	1	perennial	small	inflorescence	11.30	597.80	23.30	672.00	49.00	open	dry	oligotrophic
Campanulids	*Centaurea gymnocarpa*	18.0	9.0	1	perennial	small	inflorescence	14.90	516.60	21.40	721.00	40.00	open	dry	oligotrophic
Campanulids	*Centaurea horrida*	18.0	9.0	1	woody	small	inflorescence	16.30	476.10	19.80	554.00	61.00	open	dry	oligotrophic
Campanulids	*Centaurea ilvensis*	18.0	9.0	1	perennial	small	inflorescence	14.50	526.40	20.30	609.00	40.00	open	dry	oligotrophic
Campanulids	*Centaurea japigica*	18.0	9.0	1	woody	small	inflorescence	16.60	544.50	21.70	683.00	54.00	open	dry	oligotrophic
Campanulids	*Centaurea kartschiana* subsp. *kartschiana*	18.0	9.0	1	perennial	small	inflorescence	13.20	641.30	26.20	1147.0	17.00	open	dry	oligotrophic
Campanulids	*Centaurea lacaitae*	36.0	9.0	1	perennial	small	inflorescence	15.10	547.10	22.30	745.00	45.00	open	dry	oligotrophic
Campanulids	*Centaurea leucadea*	18.0	9.0	1	perennial	small	inflorescence	16.90	548.20	21.60	684.00	55.00	open	dry	oligotrophic
Campanulids	*Centaurea macroacantha*	20.0	10.0	1	perennial	small	inflorescence	17.10	529.10	21.20	587.00	60.00	open	dry	mesotrophic

clade	taxon	2*n*	*x*	sample	Life form	Flower type	Inflorescence	bio1	bio4	bio7	bio12	bio15	Habitat light	Habitat moisture	Habitat nutrient
Campanulids	*Centaurea montaltensis*	36.0	9.0	1	perennial	small	inflorescence	14.80	548.30	22.90	837.00	45.00	open	dry	oligotrophic
Campanulids	*Centaurea montis-borlae*	88.0	11.0	1	perennial	small	inflorescence	12.70	590.30	24.30	934.00	31.00	open	dry	oligotrophic
Campanulids	*Centaurea nobilis*	18.0	9.0	1	perennial	small	inflorescence	16.40	537.80	21.60	688.00	51.00	open	dry	oligotrophic
Campanulids	*Centaurea panormitana* subsp. *panormitana*	18.0	9.0	1	perennial	small	inflorescence	17.60	516.90	20.70	591.00	60.00	open	dry	oligotrophic
Campanulids	*Centaurea panormitana* subsp. *seguenzae*	18.0	9.0	1	perennial	small	inflorescence	15.90	557.20	22.60	661.00	54.00	open	dry	oligotrophic
Campanulids	*Centaurea panormitana* subsp. *todaroi*	18.0	9.0	1	perennial	small	inflorescence	17.70	519.60	21.10	572.00	61.00	open	dry	oligotrophic
Campanulids	*Centaurea panormitana* subsp. *umbrosa*	18.0	9.0	1	perennial	small	inflorescence	18.00	510.10	20.30	593.00	61.00	open	dry	oligotrophic
Campanulids	*Centaurea parlatoris* subsp. *parlatoris*	18.0	9.0	1	perennial	small	inflorescence	13.50	591.00	22.90	536.00	52.00	open	dry	oligotrophic
Campanulids	*Centaurea poeltiana*	36.0	9.0	1	perennial	small	inflorescence	11.60	569.00	22.50	829.00	51.00	open	moist	oligotrophic
Campanulids	*Centaurea sicana*	18.0	9.0	1	perennial	small	inflorescence	14.70	571.40	22.60	505.00	56.00	open	dry	oligotrophic
Campanulids	*Centaurea subtilis*	22.0	11.0	1	perennial	small	inflorescence	12.60	602.60	23.10	571.00	23.00	open	dry	oligotrophic
Campanulids	*Centaurea tauromenitana*	20.0	10.0	1	woody	small	inflorescence	15.30	562.80	22.90	681.00	55.00	open	dry	oligotrophic
Campanulids	*Centaurea tenacissima*	18.0	9.0	1	perennial	small	inflorescence	16.40	537.80	21.60	688.00	51.00	open	dry	oligotrophic
Campanulids	*Centaurea tenoreana*	18.0	9.0	1	perennial	small	inflorescence	6.80	613.90	23.60	837.00	20.00	open	dry	oligotrophic
Campanulids	*Centaurea tenorei*	18.0	9.0	1	perennial	small	inflorescence	13.30	566.40	22.60	739.00	42.00	open	dry	oligotrophic
Campanulids	*Centaurea veneris*	18.0	9.0	1	perennial	small	inflorescence	14.40	572.00	24.00	959.00	35.00	open	dry	oligotrophic
Caryophyllales	*Cerastium apuanum*	36.0	9.0	1	perennial	small	single	12.70	606.70	25.30	908.00	30.00	open	moist	mesotrophic
Caryophyllales	*Cerastium lacaitae*	72.0	9.0	1	perennial	small	single	14.80	571.00	22.70	557.00	56.00	open	moist	mesotrophic
Caryophyllales	*Cerastium palustre*	34.0	17.0	1	perennial	small	single	14.30	561.10	23.70	741.00	54.00	open	moist	oligotrophic
Caryophyllales	*Cerastium scaranii*	72.0	9.0	4	perennial	small	single	12.53	588.33	23.75	711.75	38.50	semi	moist	mesotrophic
Caryophyllales	*Cerastium thomasii*	36.0	9.0	1	woody	small	single	7.20	619.80	24.30	855.00	22.00	open	dry	oligotrophic
Caryophyllales	*Cerastium tomentosum*	77.1	9.0	7	woody	small	single	10.69	621.13	25.76	811.14	27.86	open	dry	oligotrophic
Caryophyllales	*Cerastium utriense*	36.0	9.0	1	perennial	small	single	10.70	603.80	25.40	1037.0	29.00	open	moist	mesotrophic
Monocots	*Charybdis glaucophylla*	20.0	10.0	1	geophyte	large	single	16.50	499.70	21.80	646.00	63.00	open	dry	oligotrophic
Campanulids	*Chiliadenus lopadusanus*	18.0	9.0	1	woody	small	inflorescence	18.70	456.50	17.10	302.00	73.00	open	dry	oligotrophic
Campanulids	*Cirsium alpis-lunae*	34.0	17.0	1	perennial	small	inflorescence	12.70	609.20	26.90	791.00	25.00	open	moist	eutrophic
Campanulids	*Cirsium bertolonii*	34.0	17.0	1	perennial	small	inflorescence	8.60	624.60	23.80	899.00	22.00	open	moist	eutrophic
Campanulids	*Cirsium vallis-demonis*	34.0	17.0	1	perennial	small	inflorescence	11.50	607.80	23.30	608.00	48.00	open	moist	eutrophic
Monocots	*Colchicum arenasii*	162.0	18.0	1	geophyte	large	single	16.30	476.10	19.80	554.00	61.00	semi	moist	eutrophic

clade	taxon	2*n*	*x*	sample	Life form	Flower type	Inflorescence	bio1	bio4	bio7	bio12	bio15	Habitat light	Habitat moisture	Habitat nutrient
Monocots	*Colchicum gonarei*	181.0	18.0	2	geophyte	large	single	13.40	574.80	23.80	766.00	52.00	shade	moist	eutrophic
Monocots	*Colchicum gracile*	81.0	16.0	2	geophyte	large	single	10.80	585.00	22.30	834.00	40.00	shade	moist	eutrophic
Monocots	*Colchicum neapolitanum*	98.0	9.0	7	geophyte	large	single	13.34	573.04	23.63	768.29	39.00	semi	moist	eutrophic
Campanulids	*Coristospermum cuneifolium*	22.0	11.0	1	perennial	small	inflorescence	11.40	623.70	26.50	853.00	25.00	open	dry	oligotrophic
Campanulids	*Crepis lacera* subsp. *titani*	8.0	4.0	1	perennial	small	inflorescence	12.80	647.20	26.30	921.00	17.00	open	dry	oligotrophic
Campanulids	*Crepis magellensis*	10.0	5.0	1	perennial	small	inflorescence	6.80	613.90	23.60	837.00	20.00	open	dry	oligotrophic
Campanulids	*Crepis vesicaria* subsp. *hyemalis*	8.0	4.0	1	annual	small	inflorescence	16.60	547.30	24.80	495.00	68.00	open	dry	oligotrophic
Monocots	*Crocus biflorus*	8.0	4.0	2	geophyte	large	single	14.45	549.45	22.30	724.00	46.50	open	moist	mesotrophic
Monocots	*Crocus etruscus*	8.0	4.0	1	geophyte	large	single	13.50	595.10	24.80	707.00	31.00	semi	moist	mesotrophic
Monocots	*Crocus ilvensis*	8.0	4.0	1	geophyte	large	single	14.50	526.40	20.30	609.00	40.00	semi	dry	oligotrophic
Monocots	*Crocus imperati*	26.0	13.0	1	geophyte	large	single	15.10	547.10	22.30	745.00	45.00	semi	moist	mesotrophic
Monocots	*Crocus minimus*	26.3	13.0	9	geophyte	large	single	13.79	515.92	20.87	688.11	48.78	open	dry	mesotrophic
Monocots	*Crocus siculus*	8.0	4.0	1	geophyte	large	single	11.50	607.80	23.30	608.00	48.00	open	moist	mesotrophic
Monocots	*Crocus suaveolens*	26.0	13.0	1	geophyte	large	single	14.90	549.30	25.30	965.00	48.00	semi	moist	mesotrophic
Campanulids	*Cryptotaenia thomasii*	12.0	6.0	1	perennial	small	single	13.80	552.50	22.10	850.00	52.00	semi	moist	mesotrophic
Lamiids	*Cymbalaria glutinosa* subsp. *brevicalcarata*	14.0	7.0	1	perennial	large	single	14.60	558.30	23.20	815.00	43.00	open	dry	oligotrophic
Lamiids	*Cymbalaria glutinosa* subsp. *glutinosa*	14.0	7.0	1	perennial	large	single	14.70	562.60	25.80	926.00	45.00	open	dry	oligotrophic
Lamiids	*Cymbalaria pallida*	14.0	7.0	1	perennial	large	single	6.80	613.90	23.60	837.00	20.00	open	dry	oligotrophic
Lamiids	*Cymbalaria pubescens*	28.0	7.0	1	perennial	large	single	14.80	571.00	22.70	557.00	56.00	open	dry	oligotrophic
Lamiids	*Cynoglossum apenninum*	24.0	12.0	1	perennial	large	single	7.40	620.10	24.30	858.00	23.00	semi	moist	oligotrophic
Lamiids	*Cynoglossum barbaricinum*	24.0	12.0	1	perennial	large	single	11.20	592.40	24.00	890.00	50.00	semi	moist	mesotrophic
Lamiids	*Cynoglossum magellense*	24.0	12.0	1	perennial	large	single	7.20	619.80	24.30	855.00	22.00	open	dry	oligotrophic
Lamiids	*Cynoglossum nebrodense* subsp. *lucanum*	24.0	12.0	1	perennial	large	single	11.10	577.40	22.10	854.00	41.00	open	dry	oligotrophic
Fabids	*Cytisus aeolicus*	52.0	13.0	1	woody	small	single	17.20	527.70	21.50	649.00	54.00	open	dry	oligotrophic
Malvids	*Daphne reichsteinii*	36.0	9.0	1	woody	large	single	10.30	706.30	29.30	826.00	25.00	open	moist	oligotrophic
Caryophyllales	*Dianthus cyatophorus*	30.0	15.0	1	woody	small	single	14.90	545.80	22.50	594.00	51.00	open	dry	mesotrophic
Caryophyllales	*Dianthus genargenteus*	30.0	15.0	1	woody	small	single	14.90	545.80	22.50	594.00	51.00	open	dry	mesotrophic
Caryophyllales	*Dianthus guliae*	30.0	15.0	1	perennial	small	single	12.80	584.30	22.80	784.00	44.00	semi	dry	oligotrophic
Caryophyllales	*Dianthus japigicum*	30.0	15.0	1	woody	small	single	16.60	544.50	21.70	683.00	54.00	semi	dry	oligotrophic

clade	taxon	2*n*	*x*	sample	Life form	Flower type	Inflorescence	bio1	bio4	bio7	bio12	bio15	Habitat light	Habitat moisture	Habitat nutrient
Caryophyllales	*Dianthus morisianus*	30.0	15.0	1	woody	small	single	15.30	522.50	22.70	671.00	59.00	semi	dry	oligotrophic
Caryophyllales	*Dianthus mossanus*	30.0	15.0	1	woody	small	single	15.00	538.70	23.60	583.00	55.00	semi	dry	oligotrophic
Caryophyllales	*Dianthus rupicola* subsp. *aeolicus*	30.0	15.0	1	woody	small	single	16.60	514.60	20.90	710.00	52.00	open	dry	oligotrophic
Lamiids	*Digitalis micrantha*	56.0	28.0	2	perennial	large	single	10.90	596.90	23.70	800.50	32.50	shade	moist	mesotrophic
Malvids	*Diplotaxis scaposa*	18.0	9.0	1	annual	large	single	18.70	456.50	17.10	302.00	73.00	open	dry	mesotrophic
Campanulids	*Dipsacus ferox*	18.0	9.0	1	perennial	small	inflorescence	13.80	569.00	24.20	866.00	57.00	open	moist	mesotrophic
Campanulids	*Dipsacus valsecchiae*	18.0	9.0	1	perennial	small	inflorescence	13.40	575.60	24.00	628.00	49.00	open	moist	mesotrophic
Fabids	*Drymocallis corsica*	28.0	7.0	1	perennial	small	single	9.10	527.30	20.00	868.00	37.00	open	dry	oligotrophic
Monocots	*Drymochloa drymeia* subsp. *exaltata*	14.0	7.0	1	geophyte	incospicious	inflorescence	11.60	569.00	22.50	829.00	51.00	open	dry	oligotrophic
Campanulids	*Echinops siculus*	30.0	15.0	1	perennial	small	inflorescence	15.30	560.80	22.20	526.00	56.00	semi	dry	mesotrophic
Campanulids	*Edraianthus graminifolius* subsp. *siculus*	32.0	16.0	1	perennial	large	single	13.50	591.00	22.90	536.00	52.00	open	dry	oligotrophic
Fabids	*Elatine gussonei*	54.0	9.0	1	perennial	large	single	18.40	501.60	21.20	520.00	80.00	open	wet	mesotrophic
Monocots	*Elytrigia corsica*	28.0	7.0	1	perennial	incospicious	inflorescence	12.20	517.00	20.70	749.00	40.00	open	moist	oligotrophic
Monocots	*Epipactis cupaniana*	38.0	19.0	1	geophyte	large	single	13.50	591.00	22.90	536.00	52.00	shade	moist	mesotrophic
Monocots	*Epipactis helleborine* subsp. *aspromontana*	38.0	19.0	1	geophyte	large	single	13.80	552.50	22.10	850.00	52.00	shade	moist	mesotrophic
Monocots	*Epipactis helleborine* subsp. *schubertiorum*	38.0	19.0	1	geophyte	large	single	13.50	591.00	22.90	536.00	52.00	shade	moist	mesotrophic
Malvids	*Erodium alpinum*	18.0	9.0	1	perennial	large	single	7.20	619.80	24.30	855.00	22.00	open	dry	oligotrophic
Malvids	*Erodium corsicum*	19.3	9.7	3	perennial	large	single	15.67	484.17	20.13	620.33	54.67	open	dry	oligotrophic
Malvids	*Erodium nervulosum*	20.0	10.0	1	perennial	large	single	16.40	556.80	23.00	641.00	50.00	open	dry	oligotrophic
Malvids	*Erucastrum nasturtiifolium* subsp. *benacense*	32.0	16.0	1	perennial	large	single	10.30	706.30	29.30	826.00	25.00	open	moist	mesotrophic
Malvids	*Erucastrum palustre*	32.0	16.0	1	perennial	large	single	13.10	654.00	27.60	1264.0	19.00	open	wet	eutrophic
Malvids	*Erucastrum virgatum* subsp. *virgatum*	14.0	7.0	1	perennial	large	single	15.30	562.80	22.90	681.00	55.00	open	dry	oligotrophic
Malvids	*Erysimum apenninum*	18.0	9.0	3	perennial	large	single	10.37	602.77	24.33	824.33	32.33	open	dry	oligotrophic
Malvids	*Erysimum aurantiacum*	14.0	7.0	1	perennial	large	single	6.70	651.50	27.10	823.00	31.00	open	dry	oligotrophic
Malvids	*Erysimum bonannianum*	24.0	12.0	2	perennial	large	single	14.70	580.65	23.45	561.00	57.00	open	dry	oligotrophic
Malvids	*Erysimum crassistylum* subsp. *crassistylum*	14.0	7.0	2	perennial	large	single	12.85	565.80	22.50	877.50	53.00	open	dry	oligotrophic
Malvids	*Erysimum crassistylum* subsp. *garganicum*	42.0	7.0	2	perennial	large	single	13.25	604.25	23.90	557.00	27.00	open	dry	oligotrophic
Malvids	*Erysimum crassistylum* subsp. *verresianum*	70.0	7.0	3	perennial	large	single	4.63	605.90	24.83	1290.0	11.33	open	dry	oligotrophic
Malvids	*Erysimum etnense*	24.0	12.0	1	perennial	large	single	15.90	570.30	24.00	586.00	62.00	open	dry	oligotrophic

clade	taxon	2*n*	*x*	sample	Life form	Flower type	Inflorescence	bio1	bio4	bio7	bio12	bio15	Habitat light	Habitat moisture	Habitat nutrient
Malvids	*Erysimum etruscum*	14.0	7.0	3	perennial	large	single	11.80	641.93	26.70	870.00	22.67	open	dry	oligotrophic
Malvids	*Erysimum insubricum*	42.0	7.0	3	perennial	large	single	9.50	650.00	27.10	1193.0	24.00	open	dry	oligotrophic
Malvids	*Erysimum ligusticum*	42.0	7.0	2	perennial	large	single	12.80	594.95	24.80	817.50	30.00	open	dry	oligotrophic
Malvids	*Erysimum majellense*	28.0	7.0	2	perennial	large	single	8.80	614.65	24.25	795.00	25.00	open	dry	oligotrophic
Malvids	*Erysimum maremmanum*	28.0	7.0	2	perennial	large	single	14.15	578.25	25.45	654.50	34.00	open	dry	oligotrophic
Malvids	*Erysimum metlesicsii*	14.0	7.0	1	perennial	large	single	17.30	501.90	20.20	446.00	63.00	open	dry	oligotrophic
Malvids	*Erysimum montis-argentarii*	28.0	7.0	1	perennial	large	single	14.80	547.80	23.00	527.00	42.00	open	dry	oligotrophic
Malvids	*Erysimum pignattii*	28.0	7.0	2	perennial	large	single	11.85	600.35	26.35	926.00	34.50	open	dry	oligotrophic
Malvids	*Erysimum pseudorhaeticum*	14.0	7.0	3	perennial	large	single	9.63	618.57	24.90	856.67	22.67	open	dry	oligotrophic
Fabids	*Euphorbia ceratocarpa*	26.0	13.0	2	perennial	incospicious	inflorescence	16.60	543.75	22.10	600.50	57.00	shade	moist	mesotrophic
Fabids	*Euphorbia corallioides*	26.0	13.0	1	perennial	incospicious	inflorescence	13.90	548.30	21.40	914.00	48.00	shade	moist	mesotrophic
Fabids	*Euphorbia gasparrinii* subsp. *samnitica*	16.0	8.0	1	perennial	incospicious	inflorescence	9.90	640.20	25.30	879.00	20.00	open	moist	oligotrophic
Fabids	*Euphorbia hyberna* subsp. *insularis*	36.0	9.0	1	perennial	incospicious	inflorescence	9.10	531.90	20.10	843.00	37.00	shade	moist	oligotrophic
Fabids	*Euphorbia meuseli*	20.0	10.0	1	perennial	incospicious	inflorescence	14.80	571.00	22.70	557.00	56.00	shade	moist	mesotrophic
Fabids	*Euphorbia papillaris*	14.0	7.0	1	perennial	incospicious	inflorescence	17.60	516.90	20.70	591.00	60.00	open	dry	oligotrophic
Fabids	*Euphorbia pithyusa* subsp. *cupanii*	36.0	9.0	1	woody	incospicious	inflorescence	15.80	538.20	23.10	637.00	59.00	open	dry	oligotrophic
Fabids	*Euphorbia semiperfoliata*	20.0	10.0	1	perennial	incospicious	inflorescence	11.20	592.40	24.00	890.00	50.00	shade	moist	mesotrophic
Fabids	*Euphorbia variabilis*	20.0	10.0	1	perennial	incospicious	inflorescence	8.40	639.60	26.30	1162.0	29.00	open	moist	oligotrophic
Campanulids	*Ferula arrigonii*	22.0	11.0	1	perennial	small	inflorescence	17.30	502.70	20.90	292.00	53.00	open	dry	oligotrophic
Campanulids	*Ferulago nodosa* subsp. *geniculata*	22.0	11.0	1	perennial	small	inflorescence	17.60	505.30	22.50	496.00	77.00	open	dry	oligotrophic
Monocots	*Festuca alfrediana* subsp. *alfrediana*	14.0	7.0	1	perennial	incospicious	inflorescence	13.40	514.10	21.40	761.00	40.00	open	dry	oligotrophic
Monocots	*Festuca alfrediana* subsp. *ferrariniana*	14.0	7.0	1	perennial	incospicious	inflorescence	9.80	610.40	23.60	895.00	26.00	open	dry	oligotrophic
Monocots	*Festuca apuanica*	70.0	7.0	1	perennial	incospicious	inflorescence	9.80	610.40	23.60	895.00	26.00	open	dry	oligotrophic
Monocots	*Festuca gamisansii* subsp. *aethaliae*	70.0	7.0	1	perennial	incospicious	inflorescence	14.50	526.40	20.30	609.00	40.00	open	dry	oligotrophic
Monocots	*Festuca humifusa*	14.0	7.0	1	perennial	incospicious	inflorescence	15.90	557.20	22.60	661.00	54.00	open	dry	oligotrophic
Monocots	*Festuca riccerii*	28.0	7.0	1	perennial	incospicious	inflorescence	7.20	611.00	23.10	876.00	21.00	open	dry	oligotrophic
Monocots	*Festuca robustifolia*	70.0	7.0	1	perennial	incospicious	inflorescence	14.10	654.80	28.30	860.00	25.00	open	dry	oligotrophic
Monocots	*Festuca sardoa*	14.0	7.0	1	perennial	incospicious	inflorescence	8.20	525.90	19.90	903.00	34.00	open	dry	oligotrophic
Monocots	*Festuca veneris*	14.0	7.0	1	perennial	incospicious	inflorescence	13.30	570.50	22.90	907.00	33.00	open	dry	oligotrophic

clade	taxon	2*n*	*x*	sample	Life form	Flower type	Inflorescence	bio1	bio4	bio7	bio12	bio15	Habitat light	Habitat moisture	Habitat nutrient
Monocots	*Festuca violacea* subsp. *italica*	14.0	7.0	2	perennial	incospicious	inflorescence	10.60	600.00	24.30	864.00	33.50	open	moist	oligotrophic
Monocots	*Festuca violacea* subsp. *puccinellii*	14.0	7.0	2	perennial	incospicious	inflorescence	9.60	614.95	23.90	894.00	24.50	open	moist	oligotrophic
Monocots	*Gagea chrysantha*	36.0	12.0	2	geophyte	large	single	14.25	553.95	22.25	698.50	55.50	semi	moist	oligotrophic
Monocots	*Gagea peruzzii*	48.0	12.0	1	geophyte	large	single	11.10	577.40	22.10	854.00	41.00	open	dry	oligotrophic
Monocots	*Gagea sicula*	24.0	12.0	1	geophyte	large	single	14.80	571.00	22.70	557.00	56.00	open	dry	oligotrophic
Monocots	*Gagea tisoniana*	24.0	12.0	2	geophyte	large	single	13.45	610.00	26.55	779.00	29.00	open	dry	oligotrophic
Lamiids	*Galium aetnicum*	44.0	11.0	2	perennial	large	single	16.00	540.60	22.00	605.50	57.00	open	dry	oligotrophic
Lamiids	*Galium caprarium*	44.0	11.0	1	perennial	large	single	14.90	516.60	21.40	721.00	40.00	open	dry	oligotrophic
Lamiids	*Galium palaeoitalicum*	20.0	10.0	2	perennial	large	single	11.90	583.85	23.20	894.00	36.00	open	dry	oligotrophic
Lamiids	*Galium pallidum*	44.0	11.0	1	perennial	large	single	14.90	593.70	28.40	862.00	37.00	open	dry	oligotrophic
Lamiids	*Galium schmidii*	44.0	11.0	1	perennial	large	single	11.20	592.40	24.00	890.00	50.00	open	dry	oligotrophic
Fabids	*Genista aquilana*	18.0	9.0	1	perennial	small	single	10.10	641.70	27.10	850.00	22.00	open	dry	oligotrophic
Fabids	*Genista arbusensis*	22.5	9.0	2	woody	small	single	16.10	512.50	22.20	603.00	59.00	open	dry	oligotrophic
Fabids	*Genista aristata*	48.0	12.0	1	woody	small	single	13.50	591.00	22.90	536.00	52.00	open	moist	oligotrophic
Fabids	*Genista bocchierii*	48.0	12.0	1	woody	small	single	15.90	522.50	23.00	538.00	57.00	open	dry	oligotrophic
Fabids	*Genista cadasonensis*	48.0	12.0	1	woody	small	single	17.30	510.30	21.30	414.00	56.00	open	dry	oligotrophic
Fabids	*Genista cilentina*	48.0	12.0	1	woody	small	single	15.50	534.60	21.60	787.00	47.00	open	dry	oligotrophic
Fabids	*Genista corsica*	48.0	12.0	2	woody	small	single	12.45	532.75	21.55	752.50	48.00	open	dry	oligotrophic
Fabids	*Genista cupanii*	48.0	12.0	1	woody	small	single	13.70	595.90	23.10	495.00	52.00	open	dry	oligotrophic
Fabids	*Genista demarcoi*	48.0	12.0	1	woody	small	single	13.50	591.00	22.90	536.00	52.00	open	dry	oligotrophic
Fabids	*Genista desoleana*	18.0	9.0	3	woody	small	single	12.20	575.73	23.90	854.00	45.67	open	dry	oligotrophic
Fabids	*Genista ephedroides*	48.0	12.0	2	woody	small	single	16.95	500.00	20.10	566.00	56.50	open	dry	oligotrophic
Fabids	*Genista etnensis*	52.0	13.0	2	woody	small	single	12.55	575.35	23.55	618.50	51.00	semi	moist	mesotrophic
Fabids	*Genista gasparrinii*	48.0	12.0	1	woody	small	single	18.00	510.10	20.30	593.00	61.00	open	dry	oligotrophic
Fabids	*Genista insularis* subsp. *fodinae*	48.0	12.0	1	woody	small	single	14.10	545.60	23.50	737.00	56.00	open	dry	oligotrophic
Fabids	*Genista insularis* subsp. *insularis*	48.0	12.0	1	woody	small	single	15.90	522.50	23.00	538.00	57.00	open	dry	oligotrophic
Fabids	*Genista madoniensis*	48.0	12.0	1	woody	small	single	13.50	591.00	22.90	536.00	52.00	open	dry	oligotrophic
Fabids	*Genista michelii*	132.0	12.0	2	woody	small	single	12.45	629.65	24.65	722.00	19.50	semi	moist	mesotrophic
Fabids	*Genista morisii*	48.0	12.0	1	woody	small	single	15.40	541.00	23.60	631.00	56.00	open	dry	oligotrophic

clade	taxon	2*n*	*x*	sample	Life form	Flower type	Inflorescence	bio1	bio4	bio7	bio12	bio15	Habitat light	Habitat moisture	Habitat nutrient
Fabids	*Genista ovina*	46.0	11.5	2	woody	small	single	15.30	522.50	22.70	671.00	59.00	open	dry	oligotrophic
Fabids	*Genista pichisemolliana*	18.0	9.0	1	woody	small	single	11.20	592.40	24.00	890.00	50.00	open	dry	oligotrophic
Fabids	*Genista salzmannii*	24.0	9.0	3	woody	small	single	14.10	539.13	22.67	737.00	49.67	open	dry	oligotrophic
Fabids	*Genista sardoa*	52.0	13.0	1	woody	small	single	16.10	487.70	21.40	589.00	63.00	open	dry	oligotrophic
Fabids	*Genista sericea* subsp. *pollinensis*	48.0	12.0	1	woody	small	single	11.10	577.40	22.10	854.00	41.00	open	dry	oligotrophic
Fabids	*Genista sulcitana*	22.5	9.0	2	woody	small	single	14.10	545.60	23.50	737.00	56.00	open	dry	oligotrophic
Fabids	*Genista toluensis*	18.0	9.0	1	woody	small	single	14.90	545.80	22.50	594.00	51.00	open	dry	oligotrophic
Fabids	*Genista tyrrhena* subsp. *pontiana*	72.0	12.0	3	woody	small	single	16.60	498.40	20.00	676.00	58.00	open	dry	oligotrophic
Fabids	*Genista tyrrhena* subsp. *tyrrhena*	48.0	12.0	1	woody	small	single	17.20	527.70	21.50	649.00	54.00	open	dry	oligotrophic
Fabids	*Genista valsecchiae*	48.0	12.0	1	woody	small	single	16.60	503.40	22.30	611.00	62.00	open	dry	oligotrophic
Lamiids	*Gentianella columnae*	36.0	18.0	1	perennial	large	single	12.70	609.60	24.90	697.00	28.00	open	moist	mesotrophic
Lamiids	*Globularia incanescens*	16.0	8.0	2	perennial	small	inflorescence	9.60	614.95	23.90	894.00	24.50	open	dry	oligotrophic
Lamiids	*Globularia neapolitana*	24.0	8.0	2	perennial	small	inflorescence	16.70	522.80	21.80	722.00	49.00	open	dry	oligotrophic
Caryophyllales	*Goniolimon italicum*	32.0	9.0	1	perennial	small	single	7.60	616.70	24.60	842.00	23.00	open	dry	oligotrophic
Caryophyllales	*Gypsophila papillosa*	34.0	17.0	1	woody	small	single	8.20	677.60	27.80	802.00	29.00	open	dry	oligotrophic
Malvids	*Helianthemum morisianum*	20.0	10.0	1	woody	large	single	14.50	564.70	24.30	629.00	53.00	open	moist	oligotrophic
Campanulids	*Helichrysum errerae*	28.0	14.0	1	woody	small	inflorescence	17.40	497.10	20.30	339.00	69.00	open	dry	oligotrophic
Campanulids	*Helichrysum litoreum*	28.0	14.0	1	woody	small	inflorescence	13.30	566.40	22.60	739.00	42.00	open	moist	oligotrophic
Campanulids	*Helichrysum nebrodense*	28.0	14.0	1	woody	small	inflorescence	13.50	591.00	22.90	536.00	52.00	open	dry	oligotrophic
Campanulids	*Helichrysum pendulum*	28.0	14.0	1	woody	small	inflorescence	17.00	509.80	21.90	425.00	58.00	open	dry	oligotrophic
Campanulids	*Helichrysum saxatile*	28.0	14.0	1	woody	small	inflorescence	13.90	560.30	23.10	684.00	51.00	open	dry	oligotrophic
Campanulids	*Hieracium cophanense*	18.0	9.0	1	perennial	small	inflorescence	16.70	518.40	21.10	530.00	59.00	open	dry	oligotrophic
Campanulids	*Hieracium grovesianum*	27.0	9.0	1	perennial	small	inflorescence	11.70	606.10	24.60	663.00	25.00	shade	moist	mesotrophic
Campanulids	*Hieracium lucidum*	18.0	9.0	1	perennial	small	inflorescence	18.00	510.10	20.30	593.00	61.00	open	dry	oligotrophic
Campanulids	*Hieracium symphytifolium*	36.0	9.0	1	perennial	small	inflorescence	13.50	591.00	22.90	536.00	52.00	open	moist	mesotrophic
Fabids	*Hypericum barbatum* subsp. *calabricum*	16.0	8.0	1	perennial	small	single	10.10	570.70	21.70	883.00	46.00	open	moist	oligotrophic
Fabids	*Hypericum hircinum* subsp. *hircinum*	20.0	10.0	1	woody	small	single	11.30	521.80	20.10	741.00	41.00	semi	wet	mesotrophic
Fabids	*Hypericum scruglii*	16.0	8.0	1	perennial	small	single	11.90	587.70	24.00	776.00	49.00	open	dry	oligotrophic
Campanulids	*Hypochaeris sardoa*	6.0	3.0	1	perennial	small	inflorescence	15.00	538.70	23.60	583.00	55.00	open	dry	oligotrophic

clade	taxon	2*n*	*x*	sample	Life form	Flower type	Inflorescence	bio1	bio4	bio7	bio12	bio15	Habitat light	Habitat moisture	Habitat nutrient
Monocots	*Iris bicapitata*	40.0	8.0	1	geophyte	large	single	13.50	615.50	24.40	547.00	22.00	open	dry	oligotrophic
Monocots	*Iris calabra*	40.0	8.0	1	geophyte	large	single	11.10	577.40	22.10	854.00	41.00	semi	moist	mesotrophic
Monocots	*Iris cengialti* subsp. *cengialti*	24.0	12.0	1	geophyte	large	single	9.20	698.80	29.10	822.00	28.00	semi	moist	oligotrophic
Monocots	*Iris marsica*	40.0	8.0	1	geophyte	large	single	7.10	613.10	23.60	808.00	22.00	semi	moist	oligotrophic
Monocots	*Iris pseudopumila*	16.0	8.0	5	geophyte	large	single	14.66	573.28	23.82	610.40	43.00	open	dry	oligotrophic
Monocots	*Iris relicta*	40.0	8.0	2	geophyte	large	single	13.45	574.85	24.35	883.50	44.50	semi	moist	mesotrophic
Monocots	*Iris revoluta*	40.0	8.0	1	geophyte	large	single	16.70	570.50	24.40	570.00	46.00	open	dry	oligotrophic
Monocots	*Iris setina*	40.0	8.0	1	geophyte	large	single	15.30	534.20	25.30	958.00	49.00	open	dry	oligotrophic
Campanulids	*Jacobaea ambigua* subsp. *ambigua*	40.0	10.0	1	woody	small	inflorescence	11.30	597.80	23.30	672.00	49.00	open	dry	oligotrophic
Campanulids	*Jacobaea ambigua* subsp. *nebrodensis*	40.0	10.0	1	woody	small	inflorescence	13.50	591.00	22.90	536.00	52.00	open	dry	oligotrophic
Campanulids	*Jacobaea lycopifolia*	60.0	10.0	2	woody	small	inflorescence	16.35	531.45	21.50	485.50	60.00	open	dry	oligotrophic
Campanulids	*Jacobaea maritima* subsp. *bicolor*	40.0	10.0	1	woody	small	inflorescence	16.50	527.40	21.70	889.00	55.00	open	dry	oligotrophic
Campanulids	*Jacobaea maritima* subsp. *gibbosa*	40.0	10.0	1	woody	small	inflorescence	14.90	547.70	22.00	832.00	53.00	open	dry	oligotrophic
Campanulids	*Jasione sphaerocephala*	14.0	7.0	1	perennial	small	inflorescence	17.40	533.60	22.70	839.00	63.00	open	dry	oligotrophic
Campanulids	*Jurinea bocconei*	34.0	17.0	1	perennial	small	inflorescence	13.50	591.00	22.90	536.00	52.00	open	dry	oligotrophic
Caryophyllales	*Kali basalticum*	54.0	9.0	1	annual	large	single	10.10	612.00	23.50	654.00	47.00	open	moist	oligotrophic
Campanulids	*Klasea flavescens* subsp. *cichoracea*	30.0	15.0	1	perennial	small	inflorescence	14.90	557.00	24.40	593.00	40.00	semi	moist	mesotrophic
Campanulids	*Knautia baldensis*	40.0	10.0	1	perennial	small	inflorescence	8.20	677.60	27.80	802.00	29.00	semi	moist	oligotrophic
Campanulids	*Knautia calycina*	20.0	10.0	1	perennial	small	inflorescence	8.80	625.80	25.20	846.00	20.00	semi	dry	oligotrophic
Campanulids	*Knautia dinarica* subsp. *silana*	30.0	10.0	2	perennial	small	inflorescence	9.20	599.00	23.20	828.50	33.50	semi	moist	mesotrophic
Campanulids	*Knautia persicina*	40.0	10.0	1	perennial	small	inflorescence	0.60	549.40	21.10	1004.0	28.00	semi	moist	oligotrophic
Campanulids	*Lactuca longidentata*	18.0	9.0	1	perennial	small	inflorescence	14.80	535.50	22.20	602.00	51.00	open	dry	oligotrophic
Lamiids	*Lamium garganicum* subsp. *corsicum*	18.0	9.0	1	perennial	large	single	13.90	560.30	23.10	684.00	51.00	semi	moist	oligotrophic
Campanulids	*Lamyropsis microcephala*	26.0	13.0	1	perennial	small	inflorescence	11.20	592.40	24.00	890.00	50.00	open	moist	oligotrophic
Campanulids	*Laserpitium siler* subsp. *siculum*	22.0	11.0	1	perennial	small	inflorescence	13.50	591.00	22.90	536.00	52.00	open	dry	oligotrophic
Fabids	*Lathyrus odoratus*	14.0	7.0	1	annual	small	single	14.70	571.40	22.60	505.00	56.00	open	moist	mesotrophic
Campanulids	*Leontodon anomalus*	8.0	4.0	1	perennial	small	inflorescence	9.80	610.40	23.60	895.00	26.00	open	dry	oligotrophic
Campanulids	*Leontodon intermedius*	8.0	4.0	1	perennial	small	inflorescence	15.40	539.60	22.40	880.00	58.00	open	dry	oligotrophic
Campanulids	*Leontodon siculus*	14.0	7.0	1	perennial	small	inflorescence	11.50	607.80	23.30	608.00	48.00	open	dry	oligotrophic

clade	taxon	2*n*	*x*	sample	Life form	Flower type	Inflorescence	bio1	bio4	bio7	bio12	bio15	Habitat light	Habitat moisture	Habitat nutrient
Campanulids	*Leucanthemum coronopifolium* subsp. *tenuifolium*	54.0	9.0	1	perennial	small	inflorescence	10.20	621.60	25.00	773.00	23.00	open	moist	oligotrophic
Campanulids	*Leucanthemum laciniatum*	18.0	9.0	1	perennial	small	inflorescence	11.10	577.40	22.10	854.00	41.00	open	dry	oligotrophic
Campanulids	*Leucanthemum ligusticum*	18.0	9.0	1	perennial	small	inflorescence	12.10	596.30	23.90	911.00	31.00	open	moist	oligotrophic
Campanulids	*Leucanthemum trydactylites*	18.0	9.0	1	perennial	small	inflorescence	9.10	618.10	25.30	833.00	26.00	open	dry	oligotrophic
Monocots	*Limodorum brulloi*	56.0	28.0	1	geophyte	large	single	13.80	552.50	22.10	850.00	52.00	shade	moist	mesotrophic
Caryophyllales	*Limonium acutifolium* subsp. *acutifolium*	18.0	9.0	1	woody	small	single	16.60	507.30	22.00	595.00	62.00	open	dry	oligotrophic
Caryophyllales	*Limonium acutifolium* subsp. *bosanum*	18.0	9.0	1	woody	small	single	16.10	519.50	22.70	642.00	61.00	open	dry	oligotrophic
Caryophyllales	*Limonium acutifolium* subsp. *cornusianum*	27.0	9.0	1	woody	small	single	16.10	519.50	22.70	642.00	61.00	open	dry	oligotrophic
Caryophyllales	*Limonium acutifolium* subsp. *nymphaeum*	18.0	9.0	1	woody	small	single	16.10	487.70	21.40	589.00	63.00	open	dry	oligotrophic
Caryophyllales	*Limonium acutifolium* subsp. *tenuifolium*	19.0	9.0	1	woody	small	single	16.60	507.30	22.00	595.00	62.00	open	dry	oligotrophic
Caryophyllales	*Limonium acutifolium* subsp. *tharrosianum*	18.0	9.0	1	woody	small	single	16.60	503.90	21.80	585.00	62.00	open	dry	oligotrophic
Caryophyllales	*Limonium aegusae*	27.0	9.0	1	woody	small	single	17.80	485.20	21.40	463.00	62.00	open	dry	oligotrophic
Caryophyllales	*Limonium albidum*	18.0	9.0	1	woody	small	single	18.70	456.50	17.10	302.00	73.00	open	dry	oligotrophic
Caryophyllales	*Limonium algusae*	27.0	9.0	1	woody	small	single	18.70	459.40	17.70	335.00	76.00	open	dry	oligotrophic
Caryophyllales	*Limonium ampuriense*	18.0	9.0	1	woody	small	single	15.50	534.40	22.30	640.00	56.00	open	dry	oligotrophic
Caryophyllales	*Limonium apulum*	18.0	9.0	1	woody	small	single	15.90	558.50	23.70	543.00	31.00	open	dry	oligotrophic
Caryophyllales	*Limonium bocconei*	18.0	9.0	1	woody	small	single	18.00	510.10	20.30	593.00	61.00	open	dry	oligotrophic
Caryophyllales	*Limonium calabrum*	26.0	9.0	1	perennial	small	single	16.90	541.60	22.70	879.00	60.00	open	dry	oligotrophic
Caryophyllales	*Limonium calcarae*	18.0	9.0	1	perennial	small	single	16.60	524.30	21.00	471.00	61.00	open	dry	oligotrophic
Caryophyllales	*Limonium capitiseliae*	27.0	9.0	1	woody	small	single	16.40	525.10	23.70	428.00	53.00	open	dry	oligotrophic
Caryophyllales	*Limonium capitismarci*	27.0	9.0	1	woody	small	single	16.60	503.90	21.80	585.00	62.00	open	dry	oligotrophic
Caryophyllales	*Limonium caprariae*	27.0	9.0	1	woody	small	single	14.90	516.60	21.40	721.00	40.00	open	dry	oligotrophic
Caryophyllales	*Limonium carisae*	27.0	9.0	1	woody	small	single	16.80	510.80	22.70	448.00	55.00	open	dry	mesotrophic
Caryophyllales	*Limonium catanzaroi*	27.0	9.0	1	perennial	small	single	17.90	504.80	20.50	472.00	64.00	open	dry	oligotrophic
Caryophyllales	*Limonium coralliforme*	27.0	9.0	1	perennial	small	single	16.10	511.70	22.00	524.00	52.00	open	dry	oligotrophic
Caryophyllales	*Limonium cosyrense*	27.0	9.0	1	woody	small	single	17.40	497.10	20.30	339.00	69.00	open	dry	oligotrophic
Caryophyllales	*Limonium cumanum*	18.0	9.0	1	perennial	small	single	15.60	540.20	24.70	869.00	47.00	open	dry	oligotrophic
Caryophyllales	*Limonium cunicularium*	27.0	9.0	1	woody	small	single	16.10	486.00	19.90	516.00	50.00	open	dry	oligotrophic
Caryophyllales	*Limonium doriae*	18.0	9.0	1	perennial	small	single	14.90	557.00	24.40	593.00	40.00	open	dry	oligotrophic

clade	taxon	2*n*	*x*	sample	Life form	Flower type	Inflorescence	bio1	bio4	bio7	bio12	bio15	Habitat light	Habitat moisture	Habitat nutrient
Caryophyllales	*Limonium dubium*	27.0	9.0	3	perennial	small	single	16.43	503.47	22.10	600.67	55.33	open	dry	oligotrophic
Caryophyllales	*Limonium etruscum*	36.0	9.0	1	perennial	small	single	14.90	557.00	24.40	593.00	40.00	open	dry	oligotrophic
Caryophyllales	*Limonium flagellare*	27.0	9.0	1	woody	small	single	17.00	522.80	20.80	579.00	59.00	open	moist	mesotrophic
Caryophyllales	*Limonium furnarii*	27.0	9.0	1	perennial	small	single	17.60	493.00	21.40	469.00	61.00	open	dry	oligotrophic
Caryophyllales	*Limonium gallurense*	27.0	9.0	1	woody	small	single	15.90	489.90	19.90	539.00	52.00	open	dry	oligotrophic
Caryophyllales	*Limonium glomeratum*	27.0	9.0	2	perennial	small	single	17.20	494.55	21.60	524.00	62.00	open	dry	oligotrophic
Caryophyllales	*Limonium gorgonae*	18.0	9.0	1	perennial	small	single	15.30	521.70	22.50	815.00	39.00	open	dry	oligotrophic
Caryophyllales	*Limonium halophilum*	27.0	9.0	1	perennial	small	single	17.60	493.00	21.40	469.00	61.00	open	dry	oligotrophic
Caryophyllales	*Limonium hermaeum*	27.0	9.0	1	woody	small	single	14.90	545.80	22.50	594.00	51.00	open	dry	oligotrophic
Caryophyllales	*Limonium hyblaeum*	36.0	9.0	1	woody	small	single	16.80	508.10	22.00	446.00	73.00	open	dry	oligotrophic
Caryophyllales	*Limonium ilvae*	18.0	9.0	1	perennial	small	single	15.40	525.00	20.60	587.00	41.00	open	dry	oligotrophic
Caryophyllales	*Limonium inarimense*	27.0	9.0	1	woody	small	single	15.50	536.40	22.60	752.00	49.00	open	dry	oligotrophic
Caryophyllales	*Limonium insulare*	27.0	9.0	1	woody	small	single	16.60	503.40	22.30	611.00	62.00	open	dry	oligotrophic
Caryophyllales	*Limonium intermedium*	32.0	9.0	1	woody	small	single	18.70	456.50	17.10	302.00	73.00	open	dry	oligotrophic
Caryophyllales	*Limonium ionicum*	35.0	9.0	1	perennial	small	single	15.30	562.80	22.90	681.00	55.00	open	dry	oligotrophic
Caryophyllales	*Limonium japygicum*	18.0	9.0	1	woody	small	single	16.70	567.10	23.90	599.00	49.00	open	dry	oligotrophic
Caryophyllales	*Limonium lacinium*	18.0	9.0	1	perennial	small	single	17.70	570.70	24.50	666.00	65.00	open	dry	oligotrophic
Caryophyllales	*Limonium laetum*	27.0	9.0	1	woody	small	single	16.30	504.10	21.70	581.00	61.00	open	dry	oligotrophic
Caryophyllales	*Limonium lausianum*	25.5	9.0	2	woody	small	single	16.60	503.90	21.80	585.00	62.00	open	dry	oligotrophic
Caryophyllales	*Limonium lilybaeum*	27.0	9.0	1	perennial	small	single	17.80	485.20	21.40	463.00	62.00	open	dry	oligotrophic
Caryophyllales	*Limonium lojaconoi*	32.0	9.0	1	perennial	small	single	17.80	485.20	21.40	463.00	62.00	open	dry	oligotrophic
Caryophyllales	*Limonium lopadusanum*	18.0	9.0	1	perennial	small	single	18.70	456.50	17.10	302.00	73.00	open	dry	oligotrophic
Caryophyllales	*Limonium malfatanicum*	27.0	9.0	1	woody	small	single	16.00	519.10	22.90	570.00	58.00	open	dry	oligotrophic
Caryophyllales	*Limonium mazarae*	36.0	9.0	1	woody	small	single	17.60	493.00	21.40	469.00	61.00	open	dry	oligotrophic
Caryophyllales	*Limonium merxmuelleri* subsp. *merxmuelleri*	18.0	9.0	1	woody	small	single	16.10	512.40	22.60	621.00	60.00	open	dry	oligotrophic
Caryophyllales	*Limonium merxmuelleri* subsp. *oristanum*	18.0	9.0	1	woody	small	single	16.60	511.70	22.30	553.00	58.00	open	dry	oligotrophic
Caryophyllales	*Limonium merxmuelleri* subsp. *sulcitanum*	18.0	9.0	1	woody	small	single	16.10	512.50	22.20	603.00	59.00	open	dry	oligotrophic
Caryophyllales	*Limonium merxmuelleri* subsp. *tigulianum*	18.0	9.0	1	woody	small	single	16.00	519.10	22.90	570.00	58.00	open	dry	oligotrophic
Caryophyllales	*Limonium minutiflorum*	25.7	8.7	3	perennial	small	single	17.33	504.17	21.37	512.67	58.67	open	dry	oligotrophic

clade	taxon	2*n*	*x*	sample	Life form	Flower type	Inflorescence	bio1	bio4	bio7	bio12	bio15	Habitat light	Habitat moisture	Habitat nutrient
Caryophyllales	*Limonium morisianum*	27.0	9.0	1	woody	small	single	15.60	539.90	22.40	476.00	50.00	open	dry	oligotrophic
Caryophyllales	*Limonium multiforme*	18.0	9.0	2	perennial	small	single	14.75	557.80	23.80	667.50	38.50	open	dry	oligotrophic
Caryophyllales	*Limonium multifurcatum*	18.0	9.0	1	woody	small	single	15.70	500.60	20.80	530.00	50.00	open	dry	oligotrophic
Caryophyllales	*Limonium optimae*	26.0	13.0	1	perennial	small	single	15.10	562.70	22.30	468.00	56.00	open	dry	oligotrophic
Caryophyllales	*Limonium opulentum*	27.0	9.0	1	perennial	small	single	17.30	501.90	20.20	446.00	63.00	open	moist	mesotrophic
Caryophyllales	*Limonium pachynense*	36.0	9.0	1	woody	small	single	17.80	499.20	22.10	447.00	79.00	open	dry	oligotrophic
Caryophyllales	*Limonium panormitanum*	18.0	9.0	1	woody	small	single	18.00	510.10	20.30	593.00	61.00	open	moist	mesotrophic
Caryophyllales	*Limonium planesiae*	18.0	9.0	1	perennial	small	single	16.50	496.10	18.80	490.00	45.00	open	dry	oligotrophic
Caryophyllales	*Limonium ponzoi*	36.0	9.0	1	perennial	small	single	17.40	501.60	21.00	506.00	60.00	open	dry	oligotrophic
Caryophyllales	*Limonium protohermaeum*	18.0	9.0	1	woody	small	single	17.30	510.30	21.30	414.00	56.00	open	dry	oligotrophic
Caryophyllales	*Limonium pseudolaetum*	27.0	9.0	1	woody	small	single	16.60	507.30	22.00	595.00	62.00	open	dry	oligotrophic
Caryophyllales	*Limonium pulviniforme*	18.0	9.0	1	woody	small	single	15.90	489.90	19.90	539.00	52.00	open	dry	oligotrophic
Caryophyllales	*Limonium racemosum*	18.0	9.0	1	woody	small	single	15.80	538.20	23.10	637.00	59.00	open	dry	oligotrophic
Caryophyllales	*Limonium remotispiculum*	18.0	9.0	1	woody	small	single	13.30	566.40	22.60	739.00	42.00	open	dry	oligotrophic
Caryophyllales	*Limonium retirameum* subsp. *retirameum*	18.0	9.0	1	perennial	small	single	16.40	525.10	23.70	428.00	53.00	open	dry	oligotrophic
Caryophyllales	*Limonium secundirameum*	36.0	9.0	1	woody	small	single	17.40	497.10	20.30	339.00	69.00	open	dry	oligotrophic
Caryophyllales	*Limonium selinuntinum*	27.0	9.0	1	woody	small	single	17.90	496.00	21.10	470.00	62.00	open	dry	oligotrophic
Caryophyllales	*Limonium sibthorpianum*	27.0	9.0	1	perennial	small	single	15.30	556.50	22.30	748.00	53.00	open	dry	oligotrophic
Caryophyllales	*Limonium sommierianum*	18.0	9.0	1	perennial	small	single	15.10	533.70	22.00	510.00	43.00	open	dry	oligotrophic
Caryophyllales	*Limonium strictissimum*	27.0	9.0	1	woody	small	single	14.00	513.80	22.20	735.00	42.00	open	dry	oligotrophic
Caryophyllales	*Limonium syracusanum*	18.0	9.0	1	woody	small	single	17.80	499.20	22.10	447.00	79.00	open	dry	oligotrophic
Caryophyllales	*Limonium tauromenitanum*	36.0	9.0	1	woody	small	single	15.30	562.80	22.90	681.00	55.00	open	dry	oligotrophic
Caryophyllales	*Limonium tenoreanum*	27.0	9.0	1	woody	small	single	15.20	544.80	23.30	830.00	45.00	open	dry	oligotrophic
Caryophyllales	*Limonium tenuiculum*	18.0	9.0	1	woody	small	single	17.00	509.80	21.90	425.00	58.00	open	dry	oligotrophic
Caryophyllales	*Limonium tibulatium*	27.0	9.0	1	woody	small	single	15.90	489.90	19.90	539.00	52.00	open	dry	oligotrophic
Caryophyllales	*Limonium todaroanum*	27.0	9.0	1	woody	small	single	16.70	518.40	21.10	530.00	59.00	open	dry	oligotrophic
Caryophyllales	*Limonium tyrrhenicum*	27.0	9.0	1	woody	small	single	17.30	510.50	21.30	390.00	55.00	open	dry	oligotrophic
Caryophyllales	*Limonium ursanum*	27.0	9.0	1	woody	small	single	15.70	500.60	20.80	530.00	50.00	open	dry	oligotrophic
Caryophyllales	*Limonium viniolae*	27.0	9.0	1	woody	small	single	15.00	516.20	20.90	620.00	52.00	open	dry	oligotrophic

clade	taxon	2*n*	*x*	sample	Life form	Flower type	Inflorescence	bio1	bio4	bio7	bio12	bio15	Habitat light	Habitat moisture	Habitat nutrient
Lamiids	*Linaria arcusangeli*	12.0	6.0	1	perennial	large	single	15.00	547.40	23.00	461.00	48.00	open	dry	oligotrophic
Lamiids	*Linaria capraria*	12.0	6.0	2	perennial	large	single	15.15	520.80	21.00	654.00	40.50	open	dry	oligotrophic
Lamiids	*Linaria pseudolaxiflora*	12.0	6.0	1	annual	large	single	18.70	459.40	17.70	335.00	76.00	open	dry	oligotrophic
Lamiids	*Linaria purpurea*	12.0	6.0	3	perennial	large	single	11.03	598.60	23.27	750.67	38.67	open	dry	oligotrophic
Fabids	*Linum punctatum* subsp. *punctatum*	18.0	9.0	1	perennial	small	single	13.50	591.00	22.90	536.00	52.00	open	dry	oligotrophic
Monocots	*Luzula calabra*	24.0	12.0	1	perennial	incospicious	inflorescence	10.20	578.10	22.10	860.00	45.00	open	moist	mesotrophic
Monocots	*Luzula spicata* subsp. *spicata*	36.0	18.0	1	perennial	incospicious	inflorescence	9.10	531.90	20.10	843.00	37.00	semi	moist	mesotrophic
Malvids	*Malva agrigentina*	66.0	22.0	2	woody	large	single	16.20	532.30	21.25	457.00	59.50	open	moist	mesotrophic
Malvids	*Matthiola incana* subsp. *pulchella*	14.0	7.0	1	perennial	large	single	17.40	497.10	20.30	339.00	69.00	open	dry	oligotrophic
Caryophyllales	*Mcneillia graminifolia* subsp. *rosanoi*	32.0	16.0	1	woody	large	single	11.40	623.70	26.50	853.00	25.00	open	dry	oligotrophic
Caryophyllales	*Mcneillia moraldoi*	32.0	16.0	1	woodyl	large	single	11.00	577.50	22.30	761.00	38.00	open	dry	oligotrophic
Lamiids	*Mentha requienii* subsp. *bistaminata*	18.0	9.0	1	perennial	large	single	14.90	516.60	21.40	721.00	40.00	open	wet	oligotrophic
Lamiids	*Mentha requienii* subsp. *requienii*	18.0	9.0	2	perennial	large	single	10.15	559.85	22.00	879.00	43.50	open	wet	oligotrophic
Fabids	*Mercurialis corsica*	64.0	8.0	1	perennial	incospicious	single	13.10	512.20	20.40	707.00	45.00	shade	moist	mesotrophic
Lamiids	*Micromeria cordata*	30.0	15.0	1	woody	large	single	11.20	590.10	23.80	849.00	49.00	open	dry	oligotrophic
Lamiids	*Micromeria graeca*	60.0	12.0	1	woody	small	single	13.30	636.40	26.60	859.00	23.00	open	dry	oligotrophic
Caryophyllales	*Minuartia glomerata* subsp. *trichocalycina*	30.0	15.0	1	woody	large	single	11.40	623.70	26.50	853.00	25.00	open	moist	oligotrophic
Caryophyllales	*Minuartia laricifolia* subsp. *ophiolitica*	26.0	13.0	1	woody	small	single	10.70	603.80	25.40	1037.0	29.00	open	dry	oligotrophic
Caryophyllales	*Moehringia dielsiana*	24.0	12.0	1	perennial	large	single	7.20	650.30	27.00	905.00	31.00	semi	moist	oligotrophic
Caryophyllales	*Moehringia glaucovirens*	24.0	12.0	1	perennial	large	single	7.00	660.20	27.20	777.00	33.00	semi	moist	oligotrophic
Caryophyllales	*Moehringia papulosa*	24.0	12.0	1	perennial	large	single	7.20	557.90	22.20	997.00	18.00	semi	moist	oligotrophic
Lamiids	*Moltkia suffruticosa* subsp. *bigazziana*	16.0	8.0	1	perennial	large	single	12.70	590.30	24.30	934.00	31.00	open	dry	oligotrophic
Lamiids	*Moltkia suffruticosa* subsp. *suffruticosa*	16.0	8.0	1	perennial	large	single	10.90	721.60	29.50	900.00	20.00	open	dry	oligotrophic
Malvids	*Morisia monantha*	14.0	7.0	1	perennial	large	single	14.20	566.80	24.10	822.00	57.00	open	wet	oligotrophic
Monocots	*Muscari botryoides* subsp. *longifolium*	36.0	9.0	1	geophyte	large	single	12.40	735.40	30.60	843.00	21.00	semi	moist	mesotrophic
Monocots	*Muscari gussonei*	18.0	9.0	1	geophyte	large	single	17.20	471.30	19.40	396.00	64.00	open	dry	oligotrophic
Lamiids	*Myosotis decumbens* subsp. *florentina*	28.0	7.0	1	perennial	large	single	12.50	620.60	27.20	819.00	24.00	shade	moist	mesotrophic
Lamiids	*Myosotis graui*	24.0	12.0	1	perennial	large	single	6.80	613.90	23.60	837.00	20.00	open	moist	oligotrophic
Lamiids	*Myosotis soleirolii*	18.0	9.0	1	perennial	large	single	8.50	531.60	19.90	869.00	35.00	open	moist	oligotrophic

clade	taxon	2*n*	*x*	sample	Life form	Flower type	Inflorescence	bio1	bio4	bio7	bio12	bio15	Habitat light	Habitat moisture	Habitat nutrient
Campanulids	*Nananthea perpusilla*	18.0	9.0	1	perennial	small	inflorescence	16.50	499.70	21.80	646.00	63.00	open	moist	oligotrophic
Monocots	*Narcissus supramontanus* subsp. *cunicularium*	20.0	10.0	1	geophyte	large	single	16.10	486.00	19.90	516.00	50.00	semi	moist	mesotrophic
Monocots	*Narcissus supramontanus* subsp. *supramontanus*	20.0	10.0	1	geophyte	large	single	13.90	560.30	23.10	684.00	51.00	semi	moist	mesotrophic
Lamiids	*Nepeta foliosa*	36.0	8.0	1	perennial	large	single	14.50	551.90	22.90	659.00	52.00	open	dry	oligotrophic
Monocots	*Nigritella buschmanniae*	100.0	20.0	1	geophyte	large	single	3.50	593.50	24.10	793.00	38.00	semi	moist	oligotrophic
Malvids	*Noccaea stilosa*	14.0	7.0	1	perennial	small	single	6.80	613.90	23.60	837.00	20.00	open	moist	oligotrophic
Malvids	*Odontarrhena argentea*	32.0	8.0	1	perennial	small	single	11.10	689.70	29.00	835.00	25.00	open	dry	oligotrophic
Malvids	*Odontarrhena bertolonii* subsp. *bertolonii*	26.7	8.0	3	perennial	small	single	12.00	651.20	27.30	852.67	22.67	open	dry	oligotrophic
Malvids	*Odontarrhena nebrodensis* subsp. *nebrodensis*	16.0	8.0	1	perennial	small	single	13.50	591.00	22.90	536.00	52.00	open	dry	oligotrophic
Malvids	*Odontarrhena tavolarae*	32.0	8.0	1	perennial	small	single	16.20	506.00	21.90	518.00	52.00	open	dry	oligotrophic
Lamiids	*Odontites bocconei*	24.0	12.0	1	woody	large	single	16.70	518.40	21.10	530.00	59.00	open	moist	oligotrophic
Campanulids	*Oenanthe lisae*	22.0	11.0	1	perennial	small	inflorescence	13.80	569.00	24.20	866.00	57.00	open	wet	mesotrophic
Monocots	*Oncostema dimartinoi*	28.0	7.0	1	geophyte	large	single	18.70	456.50	17.10	302.00	73.00	open	dry	oligotrophic
Monocots	*Oncostema sicula*	28.5	7.0	2	geophyte	large	single	15.40	556.80	23.80	485.00	63.00	semi	dry	oligotrophic
Monocots	*Oncostema ughii*	16.0	8.0	1	geophyte	large	single	17.00	509.80	21.90	425.00	58.00	open	dry	oligotrophic
Fabids	*Ononis masquilleri*	30.0	15.0	1	woody	small	single	12.90	733.10	29.70	825.00	21.00	open	moist	oligotrophic
Lamiids	*Onosma echioides* subsp. *angustifolia*	14.0	7.0	1	perennial	large	single	12.50	611.00	23.50	563.00	22.00	open	dry	oligotrophic
Lamiids	*Onosma echioides* subsp. *canescens*	14.0	7.0	1	perennial	large	single	13.50	591.00	22.90	536.00	52.00	open	dry	oligotrophic
Lamiids	*Onosma echioides* subsp. *echioides*	14.0	7.0	2	perennial	large	single	12.15	587.65	24.65	696.00	32.50	open	dry	oligotrophic
Lamiids	*Onosma helvetica* subsp. *lucana*	26.0	13.0	1	perennial	large	single	15.50	577.10	22.70	760.00	53.00	open	dry	oligotrophic
Lamiids	*Onosma helvetica* subsp. *tridentina*	28.0	13.0	1	perennial	large	single	10.00	706.80	29.60	824.00	26.00	open	dry	oligotrophic
Monocots	*Ophrys apulica*	72.0	18.0	1	geophyte	large	single	14.00	576.10	25.00	618.00	27.00	open	dry	oligotrophic
Monocots	*Ophrys bertolonii* subsp. *bertoloniiformis*	40.5	18.0	2	geophyte	large	single	13.00	613.25	23.95	555.00	22.00	semi	dry	oligotrophic
Monocots	*Ophrys biscutella*	36.0	18.0	1	geophyte	large	single	12.60	602.60	23.10	571.00	23.00	semi	moist	oligotrophic
Monocots	*Ophrys chestermanii*	36.5	18.0	2	geophyte	large	single	14.10	545.60	23.50	737.00	56.00	open	dry	oligotrophic
Monocots	*Ophrys classica*	36.0	18.0	1	geophyte	large	single	14.40	581.70	25.80	888.00	34.00	semi	moist	oligotrophic
Monocots	*Ophrys crabonifera*	37.5	18.0	2	geophyte	large	single	14.80	547.80	23.00	527.00	42.00	semi	moist	oligotrophic
Monocots	*Ophrys exaltata* subsp. *morisii*	36.0	18.0	1	geophyte	large	single	13.90	560.30	23.10	684.00	51.00	semi	dry	oligotrophic
Monocots	*Ophrys lunulata*	36.0	18.0	1	geophyte	large	single	16.10	525.80	22.30	440.00	67.00	semi	dry	oligotrophic

clade	taxon	2*n*	*x*	sample	Life form	Flower type	Inflorescence	bio1	bio4	bio7	bio12	bio15	Habitat light	Habitat moisture	Habitat nutrient
Monocots	*Ophrys oxyrrhynchos* subsp. *celiensis*	36.0	18.0	1	geophyte	large	single	15.60	578.50	25.00	576.00	38.00	semi	dry	oligotrophic
Monocots	*Ophrys oxyrrhynchos* subsp. *oxyrrhynchos*	36.0	18.0	1	geophyte	large	single	14.80	571.00	22.70	557.00	56.00	semi	dry	oligotrophic
Monocots	*Ophrys parvimaculata*	38.0	18.0	1	geophyte	large	single	13.50	615.50	24.40	547.00	22.00	semi	dry	oligotrophic
Monocots	*Ophrys peucetiae*	36.0	18.0	1	geophyte	large	single	14.20	580.50	24.50	569.00	27.00	semi	dry	oligotrophic
Monocots	*Ophrys pollinensis*	37.0	18.0	2	geophyte	large	single	13.50	584.30	23.60	681.00	38.00	semi	moist	oligotrophic
Monocots	*Ophrys promontorii*	37.3	18.0	6	geophyte	large	single	9.85	607.85	23.35	689.50	22.50	semi	moist	oligotrophic
Monocots	*Ophrys pseudomelena*	36.0	18.0	2	geophyte	large	single	15.30	563.90	24.20	626.50	37.00	semi	dry	oligotrophic
Monocots	*Ophrys sipontensis*	37.0	18.0	2	geophyte	large	single	12.60	602.60	23.10	571.00	23.00	semi	moist	oligotrophic
Monocots	*Ophrys tardands*	36.0	18.0	1	geophyte	large	single	16.60	551.70	23.40	635.00	47.00	semi	dry	oligotrophic
Monocots	*Ophrys tarentina*	36.0	18.0	1	geophyte	large	single	14.80	577.10	25.20	609.00	35.00	semi	moist	oligotrophic
Monocots	*Orchis brancifortii*	42.0	21.0	1	geophyte	large	single	15.60	551.90	21.80	582.00	57.00	semi	moist	mesotrophic
Monocots	*Orchis mascula* subsp. *ichnusae*	42.0	21.0	1	geophyte	large	single	13.90	560.30	23.10	684.00	51.00	semi	moist	mesotrophic
Monocots	*Ornithogalum corsicum*	18.0	9.0	3	geophyte	large	single	13.67	547.57	22.50	757.67	52.67	open	dry	oligotrophic
Monocots	*Ornithogalum etruscum* subsp. *etruscum*	90.0	9.0	3	geophyte	large	single	12.67	631.40	26.40	782.67	23.33	semi	moist	mesotrophic
Monocots	*Ornithogalum etruscum* subsp. *umbratile*	63.0	9.0	3	geophyte	large	single	12.80	615.63	25.10	659.00	23.67	semi	moist	mesotrophic
Monocots	*Ornithogalum exscapum*	18.0	9.0	2	geophyte	large	single	14.05	563.65	23.65	903.00	41.50	open	dry	mesotrophic
Monocots	*Ornithogalum orthophyllum* subsp. *orthophyllum*	18.0	9.0	1	geophyte	large	single	12.70	555.30	22.30	918.00	53.00	open	dry	oligotrophic
Fabids	*Oxytropis pilosa* subsp. *caputoi*	16.0	8.0	1	perennial	small	single	9.50	627.50	26.30	865.00	23.00	open	dry	oligotrophic
Monocots	*Pancratium illyricum*	22.0	11.0	2	geophyte	large	single	15.95	521.35	21.70	457.00	51.00	open	moist	oligotrophic
Campanulids	*Pastinaca kochii*	22.0	11.0	1	perennial	small	inflorescence	13.40	514.10	21.40	761.00	40.00	open	wet	mesotrophic
Campanulids	*Petagnaea gussonei*	42.0	21.0	1	perennial	small	inflorescence	15.60	562.50	22.80	594.00	54.00	shade	wet	eutrophic
Monocots	*Phalaroides arundinacea* subsp. *rotgesii*	14.0	7.0	1	perennial	incospicious	inflorescence	15.10	497.60	20.80	607.00	46.00	semi	moist	oligotrophic
Monocots	*Phleum sardoum*	28.0	7.0	1	annual	incospicious	inflorescence	16.10	512.50	22.20	603.00	59.00	open	dry	oligotrophic
Malvids	*Phyllolepidum rupestre*	16.0	8.0	1	perennial	large	single	11.40	623.70	26.50	853.00	25.00	open	dry	oligotrophic
Campanulids	*Pimpinella anisoides*	20.0	10.0	1	perennial	small	inflorescence	16.70	518.40	21.10	530.00	59.00	open	dry	mesotrophic
Lamiids	*Pinguicula apuana*	96.0	8.0	2	perennial	large	single	9.80	610.40	23.60	895.00	26.00	semi	wet	oligotrophic
Lamiids	*Pinguicula christinae*	96.0	8.0	2	perennial	large	single	9.00	622.05	24.00	896.00	22.50	open	wet	oligotrophic
Lamiids	*Pinguicula fiorii*	64.0	8.0	1	perennial	large	single	13.70	602.50	25.60	770.00	25.00	semi	wet	oligotrophic
Lamiids	*Pinguicula mariae*	32.0	8.0	1	perennial	large	single	9.80	610.40	23.60	895.00	26.00	open	wet	oligotrophic

clade	taxon	2*n*	*x*	sample	Life form	Flower type	Inflorescence	bio1	bio4	bio7	bio12	bio15	Habitat light	Habitat moisture	Habitat nutrient
Lamiids	*Pinguicula poldinii*	32.0	8.0	1	perennial	large	single	7.60	679.20	29.60	1164.0	26.00	semi	wet	oligotrophic
Lamiids	*Pinguicula sehuensis*	16.0	8.0	1	perennial	large	single	11.90	587.70	24.00	776.00	49.00	open	wet	oligotrophic
Lamiids	*Pinguicula vulgaris* subsp. *ernica*	64.0	8.0	1	perennial	large	single	10.10	622.60	26.50	874.00	26.00	open	wet	oligotrophic
Lamiids	*Pinguicula vulgaris* subsp. *vestina*	64.0	8.0	1	perennial	large	single	10.40	640.60	27.30	860.00	22.00	open	wet	oligotrophic
Campanulids	*Plagius flosculosus*	18.0	9.0	1	perennial	small	inflorescence	15.80	518.60	22.60	640.00	61.00	open	dry	oligotrophic
Lamiids	*Plantago media* subsp. *brutia*	12.0	6.0	1	perennial	small	inflorescence	11.10	577.40	22.10	854.00	41.00	open	moist	oligotrophic
Lamiids	*Plantago peloritana*	10.0	5.0	1	perennial	small	inflorescence	15.30	556.50	22.30	748.00	53.00	open	dry	oligotrophic
Lamiids	*Plantago sarda*	24.0	6.0	1	perennial	small	inflorescence	11.20	592.40	24.00	890.00	50.00	open	dry	oligotrophic
Fabids	*Polygala apiculata*	24.0	12.0	1	perennial	small	single	17.00	531.70	22.30	846.00	59.00	open	dry	oligotrophic
Fabids	*Polygala carueliana*	16.0	8.0	1	perennial	small	single	9.80	610.40	23.60	895.00	26.00	open	dry	oligotrophic
Caryophyllales	*Polygonum scoparium*	20.0	10.0	1	perennial	large	single	12.10	514.10	20.40	784.00	42.00	open	moist	oligotrophic
Caryophyllales	*Polygonum tenorei*	20.0	10.0	1	perennial	large	single	13.30	580.50	23.10	763.00	42.00	open	dry	oligotrophic
Caryophyllales	*Portulaca sardoa*	48.0	12.0	1	annual	small	single	15.20	552.50	23.70	697.00	56.00	open	moist	eutrophic
Caryophyllales	*Portulaca sicula*	36.0	12.0	1	annual	small	single	17.70	526.20	21.90	522.00	59.00	open	moist	eutrophic
Fabids	*Potentilla caulescens* subsp. *nebrodensis*	14.0	7.0	1	perennial	small	single	15.60	539.90	22.40	476.00	50.00	open	dry	oligotrophic
Fabids	*Potentilla crassinervia*	14.0	7.0	1	perennial	small	single	10.70	525.40	20.10	757.00	40.00	open	dry	oligotrophic
Fabids	*Potentilla rigoana*	14.0	7.0	1	perennial	small	single	3.60	642.10	27.70	1088.0	31.00	open	moist	oligotrophic
Monocots	*Prospero corsicum*	14.0	7.0	1	geophyte	large	single	15.40	482.80	19.20	560.00	50.00	open	dry	oligotrophic
Monocots	*Prospero hierae*	14.0	7.0	1	geophyte	large	single	17.00	509.80	21.90	425.00	58.00	open	dry	oligotrophic
Campanulids	*Pseudoscabiosa limonifolia*	18.0	9.0	1	woody	small	inflorescence	18.00	510.10	20.30	593.00	61.00	open	dry	oligotrophic
Campanulids	*Ptilostemon greuteri*	24.0	12.0	1	woody	small	inflorescence	17.40	501.60	21.00	506.00	60.00	open	dry	mesotrophic
Campanulids	*Ptilostemon niveus*	32.0	16.0	1	perennial	small	inflorescence	13.50	591.00	22.90	536.00	52.00	open	dry	mesotrophic
Campanulids	*Ptychotis sardoa*	22.0	11.0	1	perennial	small	inflorescence	16.20	506.00	21.90	518.00	52.00	open	dry	oligotrophic
Lamiids	*Pulmonaria vallarsae* subsp. *apennina*	22.0	11.0	1	perennial	large	single	9.40	632.80	24.50	909.00	21.00	shade	moist	mesotrophic
Lamiids	*Pulmonaria officinalis* subsp. *marzolae*	16.0	8.0	1	perennial	large	single	8.30	696.40	29.20	802.00	32.00	shade	moist	mesotrophic
Lamiids	*Pulmonaria vallarsae* subsp. *vallarsae*	22.0	11.0	3	perennial	large	single	11.43	611.93	24.70	768.00	26.00	shade	moist	mesotrophic
Fabids	*Quercus ichnusae*	24.0	12.0	1	woody	incospicious	single	14.90	558.80	23.90	732.00	57.00	semi	dry	mesotrophic
Fabids	*Retama raetam* subsp. *gussonei*	48.0	12.0	1	woody	small	single	17.20	471.30	19.40	396.00	64.00	open	dry	oligotrophic
Fabids	*Rhamnus glaucophylla*	24.0	12.0	1	woody	large	single	9.80	610.40	23.60	895.00	26.00	open	moist	oligotrophic

clade	taxon	2*n*	*x*	sample	Life form	Flower type	Inflorescence	bio1	bio4	bio7	bio12	bio15	Habitat light	Habitat moisture	Habitat nutrient
Fabids	*Rhamnus persicifolia*	36.0	12.0	1	woody	large	single	11.20	590.10	23.80	849.00	49.00	semi	moist	oligotrophic
Campanulids	**Rhaponticoides calabrica*	30.0	15.0	1	perennial	small	inflorescence	11.70	569.00	21.80	873.00	43.00	semi	moist	mesotrophic
Campanulids	*Rhaponticoides centaurium*	30.0	15.0	1	perennial	small	inflorescence	14.70	581.10	25.30	579.00	32.00	shade	moist	mesotrophic
Lamiids	*Rhinanthus wettsteinii*	14.0	7.0	1	annual	large	single	11.40	623.70	26.50	853.00	25.00	open	dry	oligotrophic
Malvids	*Rhizobotrya alpina*	14.0	7.0	1	perennial	small	single	1.90	587.90	24.10	1001.0	36.00	open	moist	oligotrophic
Campanulids	*Robertia taraxacoides*	8.0	4.0	2	perennial	small	inflorescence	10.20	564.85	21.70	757.50	43.00	open	dry	oligotrophic
Monocots	*Romulea bocchierii*	45.0	9.0	1	geophyte	large	single	15.10	540.70	22.70	447.00	48.00	open	moist	oligotrophic
Monocots	*Romulea insularis*	45.0	9.0	1	geophyte	large	single	14.90	516.60	21.40	721.00	40.00	open	moist	oligotrophic
Monocots	*Romulea linaresii* subsp. *linaresii*	45.0	9.0	2	geophyte	large	single	16.70	518.40	21.10	530.00	59.00	open	moist	mesotrophic
Monocots	*Romulea requienii*	36.0	9.0	1	geophyte	large	single	17.30	502.70	20.90	292.00	53.00	open	wet	oligotrophic
Monocots	*Romulea revelierei*	36.0	9.0	1	geophyte	large	single	16.10	486.00	19.90	516.00	50.00	open	moist	oligotrophic
Monocots	*Romulea variicolor*	36.0	9.0	1	geophyte	large	single	16.80	508.10	22.00	446.00	73.00	open	dry	mesotrophic
Caryophyllales	*Rumex aetnensis*	20.0	10.0	1	perennial	large	single	11.30	597.80	23.30	672.00	49.00	open	moist	oligotrophic
Caryophyllales	*Rumex scutatus* subsp. *glaucescens*	20.0	10.0	1	perennial	large	single	11.90	601.40	23.20	629.00	49.00	open	moist	oligotrophic
Malvids	*Ruta lamarmorae*	36.0	9.0	1	woody	large	single	15.10	540.70	22.70	447.00	48.00	open	dry	oligotrophic
Caryophyllales	*Sagina pilifera*	22.0	11.0	1	perennial	large	single	9.10	531.90	20.10	843.00	37.00	open	moist	oligotrophic
Fabids	*Salix crataegifolia*	38.0	19.0	1	woody	incospicious	single	9.80	610.40	23.60	895.00	26.00	open	moist	mesotrophic
Lamiids	*Salvia desoleana*	44.0	11.0	2	perennial	large	single	14.85	549.25	23.10	660.50	55.00	semi	dry	oligotrophic
Lamiids	*Salvia haematodes*	18.0	9.0	1	perennial	large	single	15.80	547.80	21.80	865.00	52.00	open	dry	oligotrophic
Lamiids	*Salvia pratensis* subsp. *saccardiana*	20.0	10.0	1	perennial	large	single	12.90	726.60	29.90	796.00	22.00	open	moist	mesotrophic
Campanulids	*Santolina corsica*	36.0	9.0	1	woody	small	inflorescence	14.80	535.50	22.20	602.00	51.00	open	dry	oligotrophic
Campanulids	*Santolina etrusca*	18.0	9.0	1	woody	small	inflorescence	13.90	634.20	27.30	780.00	28.00	open	moist	oligotrophic
Campanulids	*Santolina insularis*	54.0	9.0	1	woody	small	inflorescence	14.10	545.60	23.50	737.00	56.00	open	dry	oligotrophic
Campanulids	*Santolina leucantha*	18.0	9.0	1	woody	small	inflorescence	9.80	610.40	23.60	895.00	26.00	open	dry	oligotrophic
Campanulids	*Santolina ligustica*	18.0	9.0	1	woody	small	inflorescence	12.30	590.00	23.90	928.00	32.00	open	dry	oligotrophic
Campanulids	*Santolina neapolitana*	18.0	9.0	1	woody	small	inflorescence	15.20	544.80	23.30	830.00	45.00	open	dry	oligotrophic
Campanulids	*Scabiosa holosericea*	16.0	8.0	2	annual	small	inflorescence	11.25	587.55	23.25	889.50	35.50	open	dry	oligotrophic
Campanulids	*Scabiosa parviflora*	24.0	8.0	1	annual	small	inflorescence	15.10	562.70	22.30	468.00	56.00	open	dry	oligotrophic
Campanulids	*Scabiosa vestina*	16.0	8.0	1	annual	small	inflorescence	8.60	687.20	28.50	818.00	28.00	open	moist	oligotrophic

clade	taxon	2*n*	*x*	sample	Life form	Flower type	Inflorescence	bio1	bio4	bio7	bio12	bio15	Habitat light	Habitat moisture	Habitat nutrient
Caryophyllales	*Scleranthus aetnensis*	44.0	11.0	1	perennial	large	single	10.10	612.00	23.50	654.00	47.00	semi	moist	mesotrophic
Campanulids	*Scorzonera hispanica* subsp. *neapolitana*	14.0	7.0	1	perennial	small	inflorescence	14.60	581.70	24.60	597.00	34.00	open	dry	oligotrophic
Campanulids	*Scorzonera villosa* subsp. *columnae*	12.0	6.0	1	perennial	small	inflorescence	13.50	591.00	22.90	536.00	52.00	open	dry	oligotrophic
Lamiids	*Scrophularia morisii*	36.0	18.0	1	perennial	large	single	15.80	538.20	23.10	637.00	59.00	shade	moist	mesotrophic
Lamiids	*Scrophularia trifoliata*	70.0	24.8	4	perennial	large	single	14.43	539.05	23.03	771.75	48.00	shade	moist	eutrophic
Lamiids	*Scutellaria rubicunda*	32.0	8.0	1	perennial	large	single	14.10	585.50	23.30	538.00	56.00	shade	moist	mesotrophic
Campanulids	*Senecio nemorensis* subsp. *apuanus*	40.0	10.0	1	perennial	small	inflorescence	9.80	610.40	23.60	895.00	26.00	shade	moist	eutrophic
Campanulids	*Senecio ovatus* subsp. *stabianus*	40.0	10.0	1	perennial	small	inflorescence	9.40	619.50	24.20	893.00	23.00	shade	moist	eutrophic
Campanulids	*Senecio scopolii* subsp. *floccosus*	40.0	10.0	2	perennial	small	inflorescence	9.15	598.60	23.20	854.50	31.50	semi	moist	mesotrophic
Campanulids	*Senecio squalidus* subsp. *aethnensis*	20.0	10.0	1	perennial	small	inflorescence	11.30	597.80	23.30	672.00	49.00	semi	dry	oligotrophic
Campanulids	*Senecio squalidus* subsp. *calabrus*	20.0	10.0	1	perennial	small	inflorescence	13.80	552.50	22.10	850.00	52.00	semi	dry	oligotrophic
Campanulids	*Senecio squalidus* subsp. *chrysanthemifolius*	20.0	10.0	1	perennial	small	inflorescence	11.30	597.80	23.30	672.00	49.00	semi	dry	oligotrophic
Campanulids	*Senecio squalidus* subsp. *squalidus*	20.0	10.0	1	perennial	small	inflorescence	10.10	612.00	23.50	654.00	47.00	semi	dry	oligotrophic
Monocots	*Serapias intermedia* subsp. *hyblaea*	54.0	18.0	1	geophyte	large	single	14.60	559.60	23.10	483.00	63.00	open	dry	oligotrophic
Monocots	*Serapias orientalis* subsp. *apulica*	36.0	18.0	1	geophyte	large	single	15.80	600.50	25.60	474.00	29.00	open	dry	oligotrophic
Monocots	*Serapias orientalis* subsp. *siciliensis*	36.0	18.0	1	geophyte	large	single	16.70	497.60	20.90	417.00	66.00	open	dry	oligotrophic
Campanulids	*Seseli bocconei*	22.0	11.0	1	perennial	small	inflorescence	17.60	516.90	20.70	591.00	60.00	open	dry	oligotrophic
Campanulids	*Seseli tortuosum* subsp. *maritimum*	44.0	11.0	1	perennial	small	inflorescence	17.80	499.20	22.10	447.00	79.00	open	dry	oligotrophic
Monocots	*Sesleria barbaricina*	28.0	7.0	1	perennial	incospicious	inflorescence	13.90	560.30	23.10	684.00	51.00	semi	moist	oligotrophic
Monocots	*Sesleria calabrica*	84.0	7.0	1	perennial	incospicious	inflorescence	13.20	554.60	21.50	881.00	45.00	open	dry	mesotrophic
Monocots	*Sesleria italica*	28.0	7.0	1	perennial	incospicious	inflorescence	12.40	662.40	26.90	773.00	17.00	semi	moist	mesotrophic
Monocots	*Sesleria nitida*	42.0	7.0	1	perennial	incospicious	inflorescence	12.30	593.40	26.10	893.00	37.00	open	dry	oligotrophic
Monocots	*Sesleria pichiana*	56.0	7.0	2	perennial	incospicious	inflorescence	11.65	596.20	24.20	853.50	28.50	semi	moist	oligotrophic
Campanulids	*Siculosciadium nebrodense*	22.0	11.0	1	perennial	small	inflorescence	13.50	591.00	22.90	536.00	52.00	open	wet	mesotrophic
Caryophyllales	*Silene cattariniana*	48.0	12.0	1	perennial	small	single	8.50	627.40	24.80	862.00	23.00	semi	moist	oligotrophic
Caryophyllales	*Silene elisabethae*	24.0	12.0	1	perennial	small	single	8.60	687.20	28.50	818.00	28.00	open	dry	oligotrophic
Caryophyllales	*Silene italica* subsp. *sicula*	24.0	12.0	1	perennial	small	single	14.70	571.40	22.60	505.00	56.00	open	moist	mesotrophic
Caryophyllales	*Silene lanuginosa*	24.0	12.0	1	perennial	small	single	9.80	610.40	23.60	895.00	26.00	open	dry	oligotrophic
Caryophyllales	*Silene nodulosa*	24.0	12.0	2	perennial	small	single	15.60	522.35	22.75	550.50	53.50	open	dry	oligotrophic

clade	taxon	2*n*	*x*	sample	Life form	Flower type	Inflorescence	bio1	bio4	bio7	bio12	bio15	Habitat light	Habitat moisture	Habitat nutrient
Caryophyllales	*Silene notarisii*	24.0	12.0	1	perennial	small	single	7.20	619.80	24.30	855.00	22.00	open	dry	oligotrophic
Caryophyllales	*Silene oenotriae*	24.0	12.0	1	perennial	small	single	16.10	557.40	22.30	787.00	50.00	open	moist	mesotrophic
Caryophyllales	*Silene pichiana*	24.0	12.0	1	perennial	small	single	12.70	590.30	24.30	934.00	31.00	open	dry	oligotrophic
Caryophyllales	*Silene roemeri* subsp. *staminea*	24.0	12.0	1	perennial	large	single	7.20	619.80	24.30	855.00	22.00	open	dry	oligotrophic
Caryophyllales	*Silene rosulata* subsp. *sanctae-theresiae*	24.0	12.0	1	perennial	small	single	15.90	489.90	19.90	539.00	52.00	open	moist	mesotrophic
Caryophyllales	*Silene succulenta* subsp. *corsica*	24.0	12.0	1	perennial	small	single	12.10	514.10	20.40	784.00	42.00	open	dry	oligotrophic
Caryophyllales	*Silene valsecchiae*	24.0	12.0	1	perennial	small	single	17.30	502.70	20.90	292.00	53.00	open	dry	oligotrophic
Campanulids	*Solidago litoralis*	18.0	9.0	1	perennial	small	inflorescence	14.70	570.40	25.30	896.00	36.00	open	dry	mesotrophic
Caryophyllales	*Spergularia macrorhiza*	45.0	9.0	2	perennial	large	single	14.95	492.05	19.75	603.50	49.50	open	moist	eutrophic
Lamiids	*Stachys corsica*	18.0	9.0	1	perennial	large	single	11.20	592.40	24.00	890.00	50.00	semi	moist	oligotrophic
Lamiids	*Stachys germanica* subsp. *dasyanthes*	30.0	15.0	1	perennial	large	single	15.30	560.80	22.20	526.00	56.00	open	moist	eutrophic
Lamiids	*Stachys glutinosa*	33.0	16.5	2	woody	large	single	11.25	520.70	20.70	814.50	38.50	open	moist	oligotrophic
Lamiids	*Stachys italica*	28.0	8.0	2	perennial	small	single	13.10	607.50	25.00	686.00	38.00	open	moist	eutrophic
Lamiids	*Stachys recta* subsp. *tenoreana*	34.0	17.0	1	perennial	large	single	16.70	522.80	21.80	722.00	49.00	open	moist	oligotrophic
Lamiids	*Stachys salisii*	39.0	13.0	2	annual	large	single	14.90	516.60	21.40	721.00	40.00	semi	moist	oligotrophic
Monocots	*Stipa dasyvaginata* subsp. *apenninicola*	44.0	11.0	1	perennial	incospicious	inflorescence	11.40	612.30	26.80	935.00	32.00	open	dry	oligotrophic
Lamiids	*Symphytum gussonei*	96.0	12.0	1	perennial	large	single	14.80	571.00	22.70	557.00	56.00	shade	moist	mesotrophic
Campanulids	*Tanacetum vulgare* subsp. *siculum*	18.0	9.0	1	perennial	small	inflorescence	11.50	607.80	23.30	608.00	48.00	open	moist	mesotrophic
Campanulids	*Taraxacum aemilianum*	32.0	8.0	1	perennial	small	inflorescence	8.90	642.90	24.80	902.00	22.00	open	moist	oligotrophic
Campanulids	*Taraxacum annalisae*	32.0	8.0	1	perennial	small	inflorescence	10.40	609.50	24.20	735.00	28.00	open	moist	oligotrophic
Campanulids	*Taraxacum apenninum*	24.0	8.0	1	perennial	small	inflorescence	7.20	619.80	24.30	855.00	22.00	open	moist	oligotrophic
Campanulids	*Taraxacum caramanicae*	32.0	8.0	1	perennial	small	inflorescence	17.70	516.20	23.20	520.00	74.00	open	moist	oligotrophic
Campanulids	*Taraxacum carthusianorum*	24.0	8.0	1	perennial	small	inflorescence	12.70	555.30	22.30	918.00	53.00	open	moist	oligotrophic
Campanulids	*Taraxacum cescae*	32.0	8.0	1	perennial	small	inflorescence	13.70	545.60	21.50	918.00	49.00	open	moist	mesotrophic
Campanulids	*Taraxacum garbarianum*	32.0	8.0	1	perennial	small	inflorescence	13.50	591.00	22.90	536.00	52.00	open	moist	mesotrophic
Campanulids	*Taraxacum glaciale*	16.0	8.0	1	perennial	small	inflorescence	7.10	613.10	23.60	808.00	22.00	open	moist	oligotrophic
Campanulids	*Taraxacum kirschneri*	24.0	8.0	1	perennial	small	inflorescence	10.10	570.70	21.70	883.00	46.00	open	moist	mesotrophic
Campanulids	*Taraxacum lilianae*	24.0	8.0	1	perennial	small	inflorescence	11.10	577.40	22.10	854.00	41.00	open	moist	oligotrophic
Campanulids	*Taraxacum mannoccii*	24.0	8.0	1	perennial	small	inflorescence	14.60	569.50	25.20	852.00	35.00	open	moist	oligotrophic

clade	taxon	2*n*	*x*	sample	Life form	Flower type	Inflorescence	bio1	bio4	bio7	bio12	bio15	Habitat light	Habitat moisture	Habitat nutrient
Campanulids	*Taraxacum pollinense*	32.0	8.0	1	perennial	small	inflorescence	9.80	584.80	22.20	845.00	40.00	open	moist	mesotrophic
Campanulids	*Taraxacum vallisnibulae*	24.0	8.0	1	perennial	small	inflorescence	12.70	635.90	26.50	898.00	27.00	open	moist	eutrophic
Lamiids	*Teucrium siculum* subsp. *euganeum*	32.0	8.0	1	perennial	large	single	13.40	737.60	28.60	816.00	14.00	shade	moist	mesotrophic
Campanulids	*Thapsia garganica* subsp. *messanensis*	22.0	11.0	1	perennial	small	inflorescence	15.30	556.50	22.30	748.00	53.00	open	dry	oligotrophic
Campanulids	*Thapsia pelagica*	22.0	11.0	1	perennial	small	inflorescence	18.70	456.50	17.10	302.00	73.00	open	dry	oligotrophic
Lamiids	*Thymus paronychioides*	56.0	14.0	2	woody	large	single	14.80	571.00	22.70	557.00	56.00	open	dry	oligotrophic
Lamiids	*Thymus spinulosus*	26.0	13.0	1	woody	large	single	13.50	615.50	24.40	547.00	22.00	open	dry	oligotrophic
Campanulids	*Trachelium caeruleum* subsp. *lanceolatum*	34.0	17.0	1	perennial	small	inflorescence	14.60	559.60	23.10	483.00	63.00	semi	dry	oligotrophic
Campanulids	*Tragopogon crocifolius* subsp. *nebrodensis*	12.0	6.0	1	perennial	small	inflorescence	11.30	597.80	23.30	672.00	49.00	open	dry	oligotrophic
Campanulids	*Tragopogon porrifolius* subsp. *cupanii*	12.0	6.0	1	perennial	small	inflorescence	17.60	543.90	25.40	540.00	69.00	open	dry	oligotrophic
Fabids	*Trifolium bivonae*	32.0	8.0	1	perennial	small	inflorescence	11.50	607.80	23.30	608.00	48.00	shade	moist	mesotrophic
Fabids	*Trifolium pratense* subsp. *semipurpureum*	14.0	7.0	1	perennial	small	inflorescence	10.10	612.00	23.50	654.00	47.00	open	dry	oligotrophic
Campanulids	*Tripolium sorrentininoi*	18.0	9.0	1	perennial	small	inflorescence	13.50	591.00	22.90	536.00	52.00	open	moist	oligotrophic
Monocots	*Trisetaria gracilis*	14.0	7.0	1	perennial	incospicious	inflorescence	10.70	525.40	20.10	757.00	40.00	open	dry	oligotrophic
Monocots	*Trisetaria villosa*	14.0	7.0	1	perennial	incospicious	inflorescence	7.90	623.30	23.90	877.00	22.00	open	dry	oligotrophic
Fabids	*Urtica rupestris*	26.0	13.0	1	perennial	incospicious	single	15.40	556.80	23.80	485.00	63.00	open	dry	oligotrophic
Lamiids	*Valantia calva*	18.0	9.0	1	annual	small	single	18.70	459.40	17.70	335.00	76.00	open	dry	oligotrophic
Lamiids	*Valantia deltoidea*	36.0	9.0	1	annual	small	single	14.80	571.00	22.70	557.00	56.00	open	dry	oligotrophic
Lamiids	*Verbascum niveum* subsp. *garganicum*	32.0	8.0	1	perennial	large	single	14.30	599.30	26.10	770.00	25.00	open	moist	eutrophic
Lamiids	*Veronica aphylla* subsp. *longistyla*	18.0	9.0	1	perennial	large	single	12.70	606.70	25.30	908.00	30.00	open	moist	oligotrophic
Lamiids	*Veronica verna* subsp. *brevistyla*	16.0	8.0	1	annual	large	single	11.20	592.40	24.00	890.00	50.00	open	dry	oligotrophic
Fabids	*Vicia giacominiana*	14.0	7.0	1	annual	small	single	16.40	537.80	21.60	688.00	51.00	open	dry	oligotrophic
Fabids	*Vicia ochroleuca* subsp. *ochroleuca*	12.0	6.0	1	perennial	small	single	15.60	551.90	21.80	582.00	57.00	semi	moist	oligotrophic
Fabids	*Vicia serinica*	10.0	5.0	1	annual	small	single	10.80	585.00	22.30	834.00	40.00	open	dry	oligotrophic
Fabids	*Vicia tenuifolia* subsp. *elegans*	12.0	6.0	1	perennial	small	single	15.60	553.40	22.10	562.00	58.00	open	dry	oligotrophic
Lamiids	*Vinca difformis* subsp. *sardoa*	46.0	9.0	1	perennial	large	single	13.80	569.00	24.20	866.00	57.00	semi	moist	mesotrophic
Fabids	*Viola aetnensis* subsp. *aetnensis*	40.0	10.0	1	perennial	small	single	11.30	597.80	23.30	672.00	49.00	open	dry	oligotrophic
Fabids	*Viola aetnensis* subsp. *messanensis*	40.0	10.0	1	perennial	small	single	13.80	552.50	22.10	850.00	52.00	open	dry	oligotrophic
Fabids	*Viola aetnensis* subsp. *splendida*	40.0	10.0	1	perennial	small	single	13.00	585.60	23.60	734.00	37.00	open	dry	oligotrophic

clade	taxon	2*n*	*x*	sample	Life form	Flower type	Inflorescence	bio1	bio4	bio7	bio12	bio15	Habitat light	Habitat moisture	Habitat nutrient
Fabids	*Viola bertolonii*	20.0	10.0	1	perennial	small	single	8.90	642.90	24.80	902.00	22.00	open	dry	oligotrophic
Fabids	*Viola comollia*	22.0	11.0	1	perennial	small	single	4.10	595.60	24.10	969.00	31.00	open	dry	oligotrophic
Fabids	*Viola corsica* subsp. *ilvensis*	47.0	13.0	2	perennial	small	single	14.50	526.40	20.30	609.00	40.00	open	dry	oligotrophic
Fabids	*Viola culminis*	40.0	10.0	1	perennial	small	single	10.50	706.10	29.70	909.00	25.00	open	moist	oligotrophic
Fabids	*Viola etrusca*	40.0	10.0	1	perennial	small	single	11.70	606.10	24.60	663.00	25.00	open	moist	oligotrophic
Fabids	*Viola eugeniae* subsp. *eugeniae*	34.0	17.0	1	perennial	small	single	7.70	618.40	24.10	813.00	22.00	open	dry	oligotrophic
Fabids	*Viola eugeniae* subsp. *levieri*	34.0	17.0	1	perennial	small	single	10.90	631.10	26.90	858.00	24.00	open	dry	oligotrophic
Fabids	*Viola ferrarinii*	40.0	10.0	1	perennial	small	single	7.80	628.90	24.00	894.00	23.00	open	moist	oligotrophic
Fabids	*Viola limbarae*	52.0	13.0	1	perennial	small	single	13.80	543.20	22.10	721.00	51.00	open	dry	oligotrophic
Fabids	*Viola magellensis*	22.0	11.0	1	perennial	small	single	7.20	619.80	24.30	855.00	22.00	open	dry	oligotrophic
Fabids	*Viola merxmuelleri*	20.0	10.0	1	perennial	small	single	12.60	602.60	23.10	571.00	23.00	open	moist	oligotrophic
Fabids	*Viola nebrodensis*	20.0	10.0	1	perennial	small	single	13.50	591.00	22.90	536.00	52.00	open	dry	oligotrophic
Fabids	*Viola pseudogracilis* subsp. *cassinensis*	34.0	17.0	1	perennial	small	single	14.20	575.50	26.40	950.00	42.00	open	dry	oligotrophic
Fabids	*Viola pseudogracilis* subsp. *pseudogracilis*	34.0	17.0	1	perennial	small	single	15.20	544.80	23.30	830.00	45.00	open	dry	oligotrophic
Fabids	*Viola tineorum*	52.0	13.0	1	perennial	small	single	13.50	591.00	22.90	536.00	52.00	open	moist	oligotrophic
Fabids	*Viola ucriana*	40.0	10.0	1	perennial	small	single	13.50	591.00	22.90	536.00	52.00	open	moist	oligotrophic
Campanulids	*Xerolekia speciosissima*	20.0	10.0	1	perennial	small	inflorescence	10.50	706.10	29.70	909.00	25.00	open	moist	mesotrophic
Fabids	*Zelkova sicula*	42.0	14.0	1	woody	incospicious	single	14.60	559.60	23.10	483.00	63.00	shade	wet	eutrophic

Table S3. Summary of phylogenetic signal using different comparative methods for diploid chromosome number (2*n*) and basic chromosome number (*x*). Pagel's λ and the *K*-statistic range from no phylogenetic signal with λ and *K* = 0 to high phylogenetic signal with λ = 1, K ≥ 1. Phylogenetic signal based on the SLOUCH $t_{1/2}$ can be interpreted as follows: a $t_{1/2} > 0$ reflects an increasing association between the phylogeny and the trait ($t_{1/2}$ has the same units as the phylogeny). Pagel's λ and the *K*-statistic were calculated using the phytools package (Revell, L. J. 2012. phytools: an R package for phylogenetic comparative biology. Methods in Ecology and Evolution, 3, 217-223).

Clade	$t_{1/2}$	*K*	λ	$t_{1/2}$	*K*	λ
		2*n*			*x*	
Monocots	0.14	0.22	0.70	1.48	1.01	0.99
Fabids	0.26	0.28	0.97	0.76	0.37	0.99
Malvids	0.01	0.09	0.00	0.38	0.26	0.88
Caryophyllales	∞	0.15	0.81	∞	0.58	0.99
Lamiids	0.28	0.35	0.82	0.88	0.49	0.99
Campanulids	0.14	0.10	0.88	1.96	0.34	0.97

Figure S1. Phylogenetic tree used in the study with named tips.

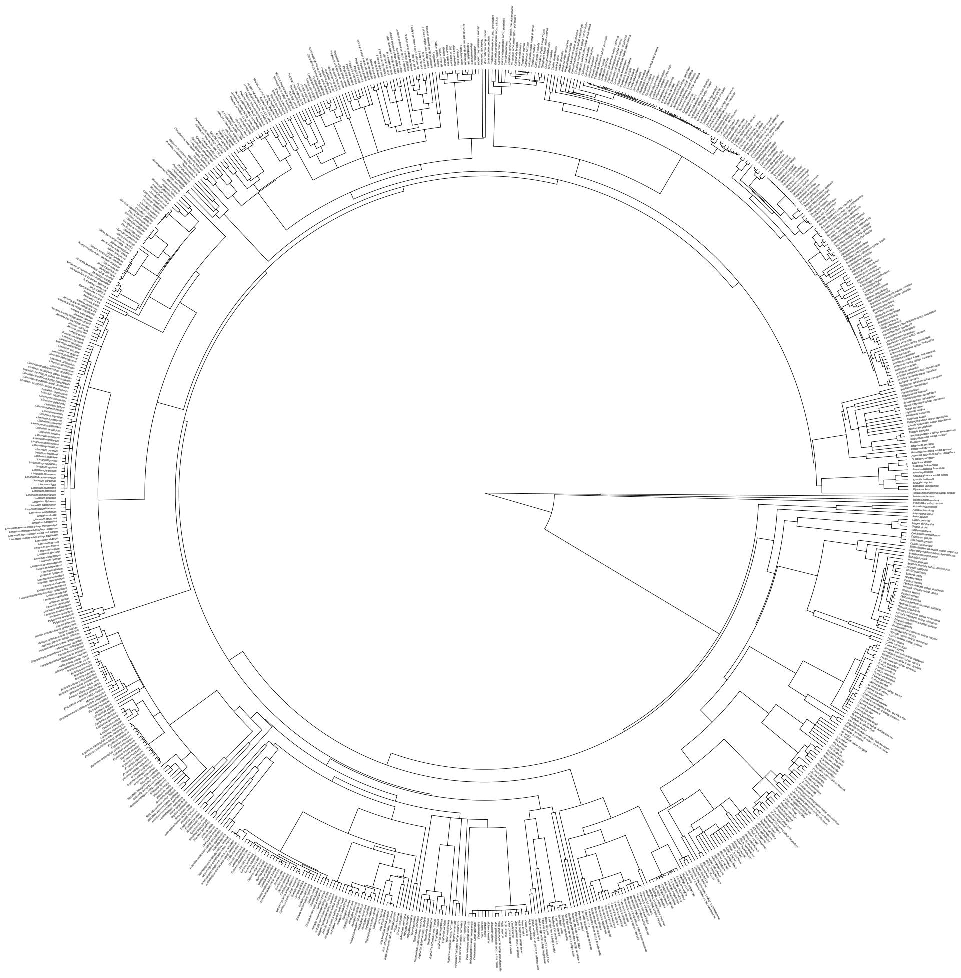

Phylogenetic tree. Phylogenetic tree used in the study in Newick format.

(((((((((((((Adoxa_moschatellina&cescae:86.699997,(((Dipsacus_ferox:9.736664,Dipsacus_valsecchiae:9.736664)N16:14.604999,(Knautia_calycina:12.170837,Knautia_baldensis:12.170837,(Knautia_dinarica&silana:6.085419,Knautia_dinarica&silana2:6.085419,Knautia_persicina:6.085419)N18:6.085416)N17:12.170833)N15:4.868333,(Pseudoscabiosa_limonifolia:19.10498,((Scabiosa_holosericea:0.399994,Scabiosa_holosericea2:0.399994,Scabiosa_vestina:0.399994)N21:8.6,Scabiosa_parviflora:9)N20:10.105)N19:10.105)N14:57.489998)N13:5.200006,(((Astrantia_pauciflora&pauciflora:10,Astrantia_pauciflora&tenorei:10)N24:30.450001,Petagnaea_gussonei:40.450012)N23:17.095833,((((Athamanta_cortiana:33.699986,Ferula_arrigonii:33.700001,(Laserpitium_siler&siculum:23.000019,Thapsia_garganica&messanensis:23,Thapsia_pelagica:23)N29:10.700001)N28:10.700001,((Bunium_corydalinum:17.975006,Cryptotaenia_thomasii:17.975006)N31:17.975,Carum_appuanum&appuanum:35.949982,Ferulago_nodosa&geniculata:35.950001,Pastinaca_kochii:35.950001,Pimpinella_anisoides:35.949993,((Seseli_bocconei:4.944458,Seseli_tortuosum&maritimum:4.944458)N33:15.555555,Siculosciadium_nebrodense:20.5)N32:15.450001)N30:8.450001,Coristospermum_cuneifolium:44.400013)N27:7.599998,(Oenanthe_lisae:26.099976,Ptychotis_sardoa:26.099976)N34:25.9)N26:3.529168,(Bupleurum_dianthifolium:39.663696,Bupleurum_dianthifolium2:39.663696,Bupleurum_falcatum&corsicum:39.663692)N35:15.865474)N25:2.016667)N22:34.354164)N12:1.799994,((((((((((((Achillea_rupestris:5.5,(Achillea_barrelieri&barrelieri:3.666687,Achillea_barrelieri&mucronulata:3.666687)N48:1.833333)N47:8.999999,((((((Anthemis_aetnensis:2,Anthemis_aetnensis2:2)N54:2,(Anthemis_cretica&calabrica:2,Anthemis_cretica&messanensis:2)N55:2,(Anthemis_cupaniana:2,Anthemis_cupaniana2:2)N56:2,Anthemis_ismelia:4)N53:2,Anthemis_arvensis&sphacelata:6,Anthemis_muricata:6)N52:4,Nananthea_perpusilla:10)N51:2,Anthemis_hydruntina&hydruntina:12)N50:1,Tanacetum_vulgare&siculum:12.999994)N49:1.5)N46:1.5,(((Leucanthemum_laciniatum:5.333313,Leucanthemum_trydactylites:5.333313,Leucanthemum_coronopifolium&tenuifolium:5.333313,Leucanthemum_ligusticum:5.333323)N59:2.666667,Plagius_flosculosus:8)N58:7,(Santolina_etrusca:11,Santolina_insularis:11,Santolina_ligustica:11,Santolina_neapolitana:11,Santolina_leucantha:11,(Santolina_corsica:5.5,Santolina_corsica2:5.5)N61:5.5)N60:4)N57:1)N45:4.5,((Artemisia_campestris&variabilis:1.5,Artemisia_campestris&variabilis2:1.5)N63:18.5,Artemisia_caerulescens&cretacea:20)N62:0.5)N44:0.5,(((Bellis_margaritaefolia:7.299988,Bellium_crassifolium:7.299988)N66:3.150001,Tripolium_sorrentininoi:10.449976)N65:3.15,Solidago_litoralis:13.599987)N64:7.4)N43:1.0625,((Castroviejoa_frigida:14.708313,Castroviejoa_montelinasana:14.708313)N68:2.451388,(Helichrysum_litoreum:11.439819,(Helichrysum_pendulum:5.71991,Helichrysum_saxatile:5.71991,Helichrysum_nebrodense:5.71991,Helichrysum_errerae:5.71991)N70:5.719907)N69:5.719907)N67:4.902778)N42:1.0625,Calendula_maritima:23.125)N41:1.0625,((Buphthalmum_inuloides:9.691956,Buphthalmum_salicifolium&flexile:9.691956,Xerolekia_speciosissima:9.69196)N72:9.691965,Chiliadenus_lopadusanus:19.383926)N71:4.803572)N40:1.0625,((Jacobaea_lycopifolia:5.833313,Jacobaea_lycopifolia2:5.833313,(Jacobaea_maritima&bicolor:4.666656,Jacobaea_maritima&gibbosa:4.666656,(Jacobaea_ambigua&ambigua:1.166656,Jacobaea_ambigua&nebrodensis:1.166656)N76:3.500001)N75:1.166667)N74:4.666667,((Senecio_scopoliifloccosus:3.929993,Senecio_scopoliifloccosus2:3.929993,(Senecio_nemorensis&apuanus:1.964996,Senecio_ovatus&stabianus:1.964996)N79:1.965)N78:3.93,(Senecio_squalidus&aethnensis:1.083313,Senecio_squalidus&calabrus:1.083313,Senecio_squalidus&chrysanthemifolius:1.083313,Senecio_squalidus&squalidus:1.083313)N80:6.776667)N77:2.64)N73:14.75)N39:2.55,(((Andryala_tenuifolia:13.375,((Hieracium_cophanense:1.625,Hieracium_symphytifolium:1.625,Hieracium_lucidum:1.625)N85:1.625,Hieracium_grovesianum:3.25)N84:10.125)N83:6.875,((((Crepis_lacera&titani:9,(Crepis_vesicaria&hyemalis:1.178589,Crepis_magellensis:1.178589)N90:7.821427)N89:3.5,(Taraxacum_aemilianum:0.5,Taraxacum_annalisae:0.5,Taraxacum_carthusianorum:0.5,Taraxacum_lilianae:0.5,Taraxacum_mannoccii:0.5,Taraxacum_caramanicae:0.5,Taraxacum_vallisnibulae:0.5,(Taraxacum_apenninum:0.25,Taraxacum_glaciale:0.25)N92:0.25,(Taraxacum_cescae:0.25,Taraxacum_garbarianum:0.25,Taraxacum_kirschneri:0.25,Taraxacum_pollinense:0.25)N93:0.25)N91:12)N88:5.6875,((Hypochaeris_sardoa:6.599994,(Leontodon_siculus:3.299988,(Leontodon_anomalus:1.649994,Leontodon_intermedius:1.649994)N97:1.65)N96:3.3)N95:7.4,Robertia_taraxacoides:14,Robertia_taraxacoides2:14)N94:4.1875)N87:1.375,Lactuca_longidentata:19.562521)N86:0.6875)N82:2.666666,((Scorzonera_hispanica&neapolitana:2.5,Scorzonera_villosa&columnae:2.5)N99:9,(Tragopogon_crocifolius&nebrodensis:2.875,Tragopogon_porrifolius&cupanii:2.875)N100:8.625)N98:11.416666)N81:4.883332)N38:5.700002,(((((((((Carduus_affinis&affinis:0.79

9988,Carduus_affinis&affinis2:0.799988,Carduus_affinis&affinis3:0.799988,Carduus_affinis&brutius:0.799988)N109:0.8,Carduus_corymbosus:1.599976,Carduus_corymbosus2:1.599976)N108:0.8,Carduus_nutans&siculus:2.399994)N107:0.8,Carduus_fasciculiflorus:3.200012)N106:0.8,(Cirsium_vallis-demonis:3,(Cirsium_alpis-lunae:1,Cirsium_bertolonii:1)N111:2)N110:1)N105:7.333334,Lamyropsis_microcephala:11.333313,Ptilostemon_greuteri:11.333313,Ptilostemon_niveus:11.333313)N104:3.666667,((((((((Centaurea_aplolepa&subciliata:0.593323,Centaurea_aplolepa&subciliata2:0.593323)N119:0.593333,Centaurea_aplolepa&maremmana:1.186646,Centaurea_veneris:1.186646)N118:0.593333,((Centaurea_cineraria&cineraria:0.593323,Centaurea_cineraria&circae:0.593323)N121:0.593333,Centaurea_aeolica&aeolica:1.186646,Centaurea_aetaliae:1.186646,Centaurea_aplolepa&cosana:1.186646,Centaurea_aplolepa&lunensis:1.186646,Centaurea_arrigoni:1.186646,Centaurea_ilvensis:1.186646)N120:0.593333,((Centaurea_filiformis&ferulacea:0.593323,Centaurea_filiformis&filiformis:0.593323)N123:0.593333,Centaurea_aplolepa&aplolepa:1.186646,Centaurea_aplolepa&carueliana:1.186646,Centaurea_gymnocarpa:1.186646)N122:0.593333)N117:0.593333,(Centaurea_lacaitae:1.186646,Centaurea_montaltensis:1.186646)N124:1.186667)N116:0.593333,(((Centaurea_corensis:0.741669,Centaurea_corensis2:0.741669)N127:0.741667,Centaurea_ambigua&nigra:1.483337,Centaurea_busambarensis:1.483337,Centaurea_diomedea:1.483337,Centaurea_erycina:1.483337,Centaurea_japigica:1.483337,Centaurea_kartschiana&kartschiana:1.483337,Centaurea_leucadea:1.483337,Centaurea_nobilis:1.483337,Centaurea_poeltiana:1.483337,Centaurea_sicana:1.483337,Centaurea_subtilis:1.483337,Centaurea_tauromenitana:1.483337,Centaurea_tenacissima:1.483337)N126:0.741667,((Centaurea_panormitana&panormitana:0.741669,Centaurea_panormitana&seguenzae:0.741669,Centaurea_panormitana&todaroi:0.741669,Centaurea_panormitana&umbrosa:0.741669)N129:0.741667,Centaurea_giardinae:1.483337,Centaurea_horrida:1.483337,Centaurea_parlatoris&parlatoris:1.483337,(Centaurea_tenoreana:0.741669,Centaurea_tenorei:0.741669)N130:0.741667)N128:0.741667)N125:0.741667)N115:0.593333,((Centaurea_arachnoidea&arachnoidea:1.186646,Centaurea_arachnoidea&montis-ferrati:1.186646)N132:1.186667,(Centaurea_ceratophylla&ceratophylla:1.186646,Centaurea_ceratophylla&ceratophylla2:1.186646,Centaurea_ceratophylla&ceratophylla3:1.186646,Centaurea_ceratophylla&ceratophylla4:1.186646)N133:1.186667,Centaurea_montis-borlae:2.373322)N131:1.186667)N114:5.936667,Centaurea_macroacantha:9.496655)N113:5.115334,Klasea_flavescens&cichoracea:14.611992,Rhaponticoides_calabrica:14.612,Rhaponticoides_centaurium:14.612)N112:0.388)N103:1.808334,Jurinea_bocconei:16.808311)N102:9.041665,(Carlina_sicula:17.233337,Carlina_sicula2:17.233337,Carlina_nebrodensis:17.233335)N134:8.616667,Echinops_siculus:25.850004)N101:7.649999)N37:43.5,((((Campanula_elatinoides:6.125,Campanula_elatinoides2:6.125)N138:6.125,(Campanula_fragilis&cavolinii:6.125,Campanula_fragilis&fragilis:6.125,Campanula_isophylla:6.125)N139:6.125,Trachelium_caeruleum&lanceolatum:12.25)N137:4.6,(((Campanula_elatines:4.5,Campanula_garganica&garganica:4.5,Campanula_reatina:4.5)N142:4.5,(Campanula_tanfanii:8,(Campanula_scheuchzeri&pollinensis:5.75,Campanula_scheuchzeri&pseudostenocodon:5.75,(Campanula_carnica&puberula:3.5,Campanula_forsythii:3.5,(Campanula_bertolae:1,Campanula_martinii:1,Campanula_sabatia:1)N146:2.5)N145:2.25)N144:2.25)N143:1)N141:5.75,Edraianthus_graminifolius&siculus:14.75)N140:2.1)N136:7.65,Jasione_sphaerocephala:24.5)N135:52.5)N36:16.699997)N11:8.400002,((((Vinca_difformis&sardoa:68,Gentianella_columnae:68.000008)N150:1,(((Asperula_aristata&calabra:4.5,Asperula_lactea:4.5,Asperula_rupestris:4.5,(Asperula_deficiens:2.25,Asperula_staliana&diomedea:2.25)N154:2.25,Asperula_apuana:4.5)N153:6,(Valantia_calva:3.5,Valantia_deltoidea:3.5)N155:7)N152:1.5,((Galium_aetnicum:2.666687,Galium_aetnicum2:2.666687,Galium_caprarium:2.666687,Galium_pallidum:2.666687,Galium_schmidii:2.666687)N157:2.666667,(Galium_palaeoitalicum:1.333313,Galium_palaeoitalicum2:1.333313)N158:3.999999)N156:6.666666)N151:57)N149:20.050003,((((Ajuga_tenorei:27.642853,Ajuga_tenorei2:27.642853)N162:13.821428,Teucrium_siculum&euganeum:41.464283)N161:13.821426,((((Betonica_alopecuros&divulsa:29.918365,(((Stachys_salisii:6.648529,Stachys_salisii2:6.648529)N169:6.648526,Stachys_corsica:13.297055)N168:6.648526,((Stachys_germanica&dasyanthes:9.972778,(Stachys_italica:4.986389,Stachys_italica2:4.986389)N172:4.986394)N171:4.986395,(Stachys_recta&tenoreana:9.972778,(Stachys_glutinosa:4.986389,Stachys_glutinosa2:4.986389)N174:4.986394)N173:4.986395)N170:4.986394)N167:9.97279)N166:4.986395,Lamium_garganicum&corsicum:34.90477)N165:13.961908,Scutellaria_rubicunda:48.866684)N164:3.490475,(((Mentha_requienii&bistaminata:8.5,Mentha_requienii&requienii:8.5,Mentha_requienii&requienii2:8.5)N177:14,(Micromeria_cordata:11.25,Micromeria_graeca:11.25)N178:11.25,((Thymus_paronychioides:2.5,Thymus_paronychioides2:2.5)N180:2.7

5,Thymus_spinulosus:5.25)N179:17.25)N176:24,(Nepeta_foliosa:42.499981,((Salvia_desoleana:3.166656,Salvia_desoleana2:3.166656)N183:3.166667,(Salvia_haematodes:3.166656,Salvia_pratensis&saccardiana:3.166656)N184:3.166667)N182:36.166668)N181:4)N175:5.857144)N163:2.92857)N160:11.714285,(Pinguicula_poldinii:38.285706,(Pinguicula_sehuensis:28.714294,(Pinguicula_fiorii:19.142853,Pinguicula_mariae:19.142853,Pinguicula_vulgaris&ernica:19.142853,Pinguicula_vulgaris&vestina:19.142853,(Pinguicula_christinae:9.571411,Pinguicula_christinae2:9.571411)N188:9.571428,(Pinguicula_apuana:9.571411,Pinguicula_apuana2:9.571411)N189:9.571428)N187:9.571428)N186:9.571428)N185:28.714285,(Odontites_bocconei:24.999998,Rhinanthus_wettsteinii:25)N190:42,(((Anarrhinum_corsicum:20.187481,(Cymbalaria_pubescens:10.583323,Cymbalaria_glutinosa&brevicalcarata:10.583313,Cymbalaria_glutinosa&glutinosa:10.583313,Cymbalaria_pallida:10.583313)N194:9.604167)N193:4.3125,((Linaria_pseudolaxiflora:8,(Linaria_arcusangeli:2,(Linaria_capraria:1,Linaria_capraria2:1)N198:1,(Linaria_purpurea2:1,Linaria_purpurea3:1)N199:1)N197:6)N196:3.5,Linaria_purpurea:11.5)N195:13)N192:31.833332,(((Digitalis_micrantha:24.200012,Digitalis_micrantha2:24.200012)N202:16.133333,((Plantago_media&brutia:15,(Plantago_peloritana:10,Plantago_sarda:10)N205:5)N204:20,(Veronica_aphylla&longistyla2:26.633333,Veronica_verna&brevistyla:26.633305)N206:8.366665)N203:5.333332)N201:5.333336,((Globularia_incanescens:1.224976,Globularia_incanescens2:1.224976)N208:1.225,(Globularia_neapolitana:1.224976,Globularia_neapolitana2:1.224976)N209:1.225)N207:43.216667)N200:10.666664)N191:10.666669,((Scrophularia_morisii:19.039978,(Scrophularia_trifoliata2:9.519989,Scrophularia_trifoliata3:9.519989,Scrophularia_trifoliata4:9.519989,Scrophularia_trifoliata5:9.519989)N212:9.52)N211:13.205,Verbascum_niveum&garganicum:32.24498)N210:34.755001)N159:22.050003)N148:5.450001,((((Aegonychon_calabrum:23,Moltkia_suffruticosa&bigazziana:23,Moltkia_suffruticosa&suffruticosa:23)N216:11,((Onosma_helvetica&lucana:2.75,Onosma_helvetica&tridentina:2.75)N218:2.75,Onosma_echioides&angustifolia:5.5,Onosma_echioides&canescens2:5.5,Onosma_echioides&echioides:5.5,Onosma_echioides&echioides2:5.5)N217:28.5)N215:12.5,((((Anchusa_capellii:2.299988,Anchusa_formosa:2.299988,Anchusa_montelinasana:2.299988)N222:2.3,(Anchusa_crispa&crispa:2.299988,Anchusa_crispa&maritima:2.299988,Anchusa_litorea:2.299988,Anchusa_sardoa:2.299988)N223:2.3)N221:28.9,(((Borago_morisiana:10.5,Borago_morisiana2:10.5,Borago_morisiana3:10.5)N226:10.5,(Borago_pygmaea:10.5,Borago_pygmaea2:10.5,Borago_pygmaea3:10.5,Borago_pygmaea4:10.5,Borago_pygmaea5:10.5,Borago_pygmaea6:10.5,Borago_pygmaea7:10.5)N227:10.5)N225:10.5,Symphytum_gussonei:31.5)N224:2)N220:4.333332,(Pulmonaria_officinalis&marzolae:17.699995,Pulmonaria_hirta&apennina:17.700006,Pulmonaria_vallarsae:17.700012,Pulmonaria_vallarsae2:17.700012,Pulmonaria_vallarsae3:17.700012)N228:20.133331)N219:8.666669)N214:1,((Cynoglossum_apenninum:11.166656,Cynoglossum_barbaricinum:11.166656,Cynoglossum_magellense:11.166656,Cynoglossum_nebrodense&lucanum:11.166656)N230:22.333334,(Myosotis_graui:13.5,Myosotis_soleirolii:13.5,Myosotis_decumbens&florentina:13.5)N231:20)N229:14)N213:47)N147:7.599998)N10:4.75,(Androsace_vitaliana&praetutiana:41.749992,((((Primula_palinuri:8.25,Primula_palinuri2:8.25)N236:8.25,Primula_glaucescens:16.5,Primula_glaucescens2:16.5,Primula_polliniana:16.5)N235:8.25,Primula_apennina:24.75)N234:11.75,(Soldanella_calabrella:0.413332,Soldanella_minima&samnitica:0.413332)N237:36.086666)N233:5.25)N232:65.099998)N9:4.650001,(((Kali_basalticum:59.324989,((((((Arenaria_bertolonii:13,Arenaria_huteri:13)N246:3.360001,(Moehringia_glaucovirens:16,(Moehringia_dielsiana:8,Moehringia_papulosa:8)N248:8)N247:0.360001)N245:13.08,(Cerastium_palustre:2.552002,(Cerastium_tomentosum:0.96,Cerastium_tomentosum2:0.959991,Cerastium_tomentosum3:0.959991,Cerastium_tomentosum4:0.959991,Cerastium_tomentosum5:0.959991,Cerastium_tomentosum6:0.959991,Cerastium_tomentosum7:0.959991,(Cerastium_apuanum:0.640015,Cerastium_lacaitae:0.640015,Cerastium_scaranii:0.640015,Cerastium_scaranii2:0.640015,Cerastium_scaranii3:0.640015,Cerastium_scaranii4:0.640015,Cerastium_thomasii:0.640015,Cerastium_utriense:0.640015)N251:0.32)N250:1.592)N249:26.888)N244:2.129999,(((Dianthus_rupicola&aeolicus:4.529999,(Dianthus_cyatophorus:2.019989,Dianthus_genargenteus:2.019989,Dianthus_japigicum:2.019989,Dianthus_morisianus:2.019989,Dianthus_mossanus:2.019989,Dianthus_guliae:2.02001)N255:2.51)N254:16.709999,Gypsophila_papillosa:21.239973)N253:9.49,(Silene_elisabethae:11.745,(((Silene_nodulosa:1.333313,Silene_nodulosa2:1.333313)N259:1.333333,(Silene_cattariniana:1.333313,Silene_pichiana:1.333313)N260:1.333333)N258:1.333333,Silene_italica&sicula:4,Silene_lanuginosa:4,Silene_oenotriae:4,Silene_roemeri&staminea:4,Silene_rosulata&sanctae-theresiae:4,Silene_notarisii:4)N257:7.745,Silene_succulenta&corsica:11.744995,Silene_valsecchiae:11.744995)N256:18.985001)N252:0.84)N243:0.68,(((Mcneillia_graminifolia&rosanoi:5.013306,Mcneillia_moraldoi:5.013306,(Minuartia_glomerata&trichocalycina:2.506653,Minuartia_laricifolia&ophiolitica:

2.506653)N264:2.506667)N263:2.506667,Scleranthus_aetnensis:7.52)N262:22.52,Sagina_pilifera:30.040009)N261:2.209999)N242:2.689999,(Spergularia_macrorhiza:8.529999,Spergularia_macrorhiza2:8.529999)N265:26.409998)N241:24.385002)N240:4.935001,(Portulaca_sardoa:23.599976,Portulaca_sicula:23.599976)N266:40.66)N239:19.740002,((((Armeria_brutia:4.5,Armeria_denticulata:4.5,Armeria_gracilis&gracilis:4.5,Armeria_gracilis&majellensis:4.5,Armeria_nebrodensis:4.5,Armeria_saviana:4.5)N270:4.5,(Armeria_sardoa&genargentea:4.5,Armeria_sardoa&sardoa:4.5,Armeria_sardoa&sardoa2:4.5,Armeria_sulcitana:4.5,Armeria_gussonei:4.5,Armeria_morisii:4.5)N271:4.5)N269:4.5,Goniolimon_italicum:13.5,((Limonium_lacinium:6,Limonium_tibulatium:6,Limonium_ampuriense:6,Limonium_calcarae:6,(Limonium_dubium:3,Limonium_dubium2:3,Limonium_dubium3:3)N274:3,Limonium_pulviniforme:6,Limonium_sibthorpianum:6,Limonium_todaroanum:6,(Limonium_glomeratum:3,Limonium_glomeratum2:3)N275:3,Limonium_cumanum:6)N273:3,((Limonium_acutifolium&acutifolium:3,Limonium_acutifolium&bosanum:3,Limonium_acutifolium&cornusianum:3,Limonium_acutifolium&nymphaeum:3,Limonium_acutifolium&tenuifolium:3,Limonium_acutifolium&tharrosianum:3)N277:3,Limonium_racemosum:6,Limonium_capitismarci:6,Limonium_cunicularium:6,Limonium_gallurense:6,Limonium_laetum:6,Limonium_pseudolaetum:6,Limonium_viniolae:6)N276:3,(Limonium_caprariae:4.5,Limonium_coralliforme:4.5)N278:4.5,(Limonium_cosyrense:6,Limonium_remotispiculum:6,Limonium_tenuiculum:6,Limonium_insulare:6,Limonium_lausianum:6,Limonium_lausianum2:6,Limonium_morisianum:6,Limonium_strictissimum:6,Limonium_tyrrhenicum:6,Limonium_ursanum:6,(Limonium_bocconei:3,Limonium_flagellare:3,Limonium_ponzoi:3,Limonium_syracusanum:3,Limonium_apulum:3,Limonium_japygicum:3)N280:3,(Limonium_hermaeum:3,Limonium_protohermaeum:3)N281:3,(Limonium_gorgonae:3,Limonium_ilvae:3,Limonium_multiforme:3,Limonium_multiforme2:3,Limonium_planesiae:3,Limonium_sommierianum:3)N282:3)N279:3,(Limonium_aegusae:4.5,Limonium_lilybaeum:4.5,Limonium_pachynense:4.5,Limonium_secundirameum:4.5,Limonium_selinuntinum:4.5,Limonium_doriae:4.5,Limonium_etruscum:4.5,Limonium_halophilum:4.5)N283:4.5,(Limonium_merxmuelleri&merxmuelleri:4.5,Limonium_merxmuelleri&oristanum:4.5,Limonium_merxmuelleri&sulcitanum:4.5,Limonium_merxmuelleri&tigulianum:4.5)N284:4.5,(Limonium_calabrum:6,Limonium_furnarii:6,Limonium_inarimense:6,Limonium_ionicum:6,Limonium_lojaconoi:6,(Limonium_minutiflorum:3,Limonium_minutiflorum2:3,Limonium_minutiflorum3:3)N286:3,Limonium_optimae:6,Limonium_tauromenitanum:6,Limonium_tenoreanum:6)N285:3,(Limonium_albidum:4.5,Limonium_hyblaeum:4.5,Limonium_intermedium:4.5,Limonium_lopadusanum:4.5,Limonium_mazarae:4.5,Limonium_panormitanum:4.5)N287:4.5,(Limonium_retirameum&retirameum:4.5,Limonium_algusae:4.5,Limonium_capitiseliae:4.5,Limonium_carisae:4.5,Limonium_catanzaroi:4.5,Limonium_malfatanicum:4.5,Limonium_multifurcatum:4.5,Limonium_opulentum:4.5)N288:4.5)N272:4.5)N268:23.5,((Polygonum_scoparium:15,Polygonum_tenorei:15)N290:10,(Rumex_aetnensis:19.25,Rumex_scutatus&glaucescens:19.25)N291:5.75)N289:12)N267:47)N238:27.5)N8:3.100002,Thesium_italicum:114.599991)N7:3.599998,(((((((((((Alyssum_diffusum&calabricum:5.361115,Alyssum_diffusum&diffusum:5.361115,Alyssum_diffusum&garganicum:5.361115)N302:10.722222,(((Odontarrhena_bertolonii&bertolonii2:3.216675,Odontarrhena_bertolonii&bertolonii3:3.216675)N305:3.216666,Odontarrhena_tavolarae:6.43335)N304:3.216667,Odontarrhena_nebrodensis&nebrodensis:9.649994,(Odontarrhena_argentea:4.825012,Odontarrhena_bertolonii&bertolonii:4.825012)N306:4.825)N303:6.433332)N301:3.216666,Phyllolepidum_rupestre:19.299994)N300:12.325,(Arabis_collina&rosea:6.01999,Aubrieta_deltoidea&sicula:6.02002)N307:25.605,((((((Brassica_macrocarpa:2.265015,Brassica_raimondoi:2.265015,Brassica_tyrrhena:2.265015)N313:2.265,Brassica_rupestris:4.529999,Brassica_rupestris2:4.529999,(Brassica_villosa&bivonana:2.265015,Brassica_villosa&drepanensis:2.265015,Brassica_villosa&tinei:2.265015,Brassica_villosa&villosa:2.265015)N314:2.265)N312:2.265,Brassica_glabrescens:6.794983)N311:2.265,(Erucastrum_virgatum&virgatum:4.529999,Morisia_monantha:4.529999)N315:4.53,Erucastrum_nasturtiifolium&benacense:9.059999)N310:4.529999,Diplotaxis_scaposa:13.590005)N309:2.265,Erucastrum_palustre:15.85499)N308:15.769999,(Noccaea_stilosa:26.354145,Rhizobotrya_alpina:26.354145)N316:5.270834)N299:0.675,(((Barbarea_rupicola:6.765015,Barbarea_rupicola2:6.765015)N319:8.945,((((Cardamine_apennina:1.570984,Cardamine_apennina2:1.570984)N323:1.571,Cardamine_granulosa:3.141998,Cardamine_silana:3.141998)N322:4.713,Cardamine_battagliae:7.85499)N321:4.713,(Cardamine_monteluccii:6.283997,Cardamine_monteluccii2:6.283997)N324:6.284)N320:3.142)N318:8.136666,(((Erysimum_crassistylum&crassistylum:1.27002,Erysimum_crassistylum&crassistylum2:1.27002)N327:1.27,Erysimum_metlesicsii:2.539978,(Erysimum_etruscum:1.27002,Erysimum_etruscum2:1.27002,Erysimum_etruscum3:1.27002)N328:1.27,(Erysimum_crassistylum&verresianum:1.27002,Erysimum_crassistylum&verresianum2:1.27002,Erysimum_crassistylum&verresianum3:1.27002)N

329:1.27)N326:1.27,((Erysimum_apenninum:1.27002,Erysimum_apenninum2:1.27002,Erysimum_apenninum3:1.27002)N331:1.27,(Erysimum_bonannianum:1.27002,Erysimum_bonannianum2:1.27002)N332:1.27,Erysimum_etnense:2.539978,(Erysimum_pignattii:1.27002,Erysimum_pignattii2:1.27002)N333:1.27)N330:1.27,((Erysimum_insubricum:1.27002,Erysimum_insubricum2:1.27002,Erysimum_insubricum3:1.27002)N335:1.27,(Erysimum_ligusticum:1.27002,Erysimum_ligusticum2:1.27002)N336:1.27,(Erysimum_maremmanum:1.27002,Erysimum_maremmanum2:1.27002)N337:1.27,Erysimum_montis-argentarii:2.539978)N334:1.27,((Erysimum_majellense:1.27002,Erysimum_majellense2:1.27002)N339:1.27,(Erysimum_pseudorhaeticum:1.27002,Erysimum_pseudorhaeticum2:1.27002,Erysimum_pseudorhaeticum3:1.27002)N340:1.27,Erysimum_aurantiacum:2.539978)N338:1.27)N325:20.036667)N317:8.453333,(Biscutella_maritima:19.380005,((Biscutella_laevigata&australis:6.459991,Biscutella_laevigata&lucia:6.459991,Biscutella_laevigata&raffaelliana:6.459991)N343:6.46,Biscutella_apuana:12.919983,Biscutella_incana:12.919983,Biscutella_mollis:12.919983,Biscutella_prealpina:12.919983,(Biscutella_pichiana&ilvensis:6.459991,Biscutella_pichiana&pichiana:6.459991,Biscutella_pichiana&pichiana2:6.459991)N344:6.46)N342:6.459999)N341:12.920001,Matthiola_incana&pulchella:32.299992)N298:5.200001,(Erysimum_crassistylum&garganicum:18.75,Erysimum_crassistylum&garganicum2:18.75)N345:18.75)N297:53.900002,((Helianthemum_morisianum:37.999989,(Malva_agrigentina:6,Malva_agrigentina2:6)N348:32)N347:15,Daphne_reichsteinii:52.999992)N346:38.400002)N296:7.700004,(Ruta_lamarmorae:80.5,Acer_cappadocicum&lobelii:80.5)N349:18.600008)N295:9.049995,(Erodium_alpinum:18.5,(Erodium_nervulosum:6.809998,(Erodium_corsicum:2.869995,Erodium_corsicum2:2.869995,Erodium_corsicum3:2.869995)N352:3.94)N351:11.690001)N350:89.650002)N294:0.849998,((((Bryonia_marmorata:89.999992,Quercus_ichnusae:90)N356:5,(((Rhamnus_glaucophylla:6.400012,Rhamnus_persicifolia:6.400012)N359:60.599998,(Zelkova_sicula:55,Urtica_rupestris:55)N360:12)N358:14.449997,(Drymocallis_corsica:28.850006,(Potentilla_caulescens&nebrodensis:10.325012,Potentilla_crassinervia:10.325012,Potentilla_rigoana:10.325012)N362:18.525002)N361:52.599995)N357:13.550003)N355:2.699997,(((((Adenocarpus_complicatus&bivonae:3.333313,Adenocarpus_complicatus&brutius:3.333313,Adenocarpus_complicatus&commutatus:3.333313,(Adenocarpus_complicatus&samniticus:1.666656,Adenocarpus_complicatus&samniticus2:1.666656)N368:1.666667)N367:4.666667,Cytisus_aeolicus:8)N366:3,(((((Genista_arbusensis:2.019989,Genista_arbusensis2:2.019989)N373:2.02,(Genista_desoleana:2.019989,Genista_desoleana2:2.019989,Genista_desoleana3:2.019989)N374:2.02,Genista_pichisemolliana:4.039978,Genista_toluensis:4.039978,(Genista_salzmannii:2.019989,Genista_salzmannii2:2.019989,Genista_salzmannii3:2.019989)N375:2.02,(Genista_sulcitana:2.019989,Genista_sulcitana2:2.019989)N376:2.02)N372:2.02,((Genista_corsica:2.019989,Genista_corsica2:2.019989)N378:2.02,Genista_cadasonensis:4.039978,Genista_morisii:4.039978)N377:2.02,(Genista_bocchierii:4.039978,Genista_valsecchiae:4.039978,(Genista_ephedroides:2.019989,Genista_ephedroides2:2.019989)N380:2.02,(Genista_insularis&fodinae:2.019989,Genista_insularis&insularis:2.019989)N381:2.02,(Genista_ovina:2.019989,Genista_ovina2:2.019989)N382:2.02)N379:2.02,Genista_cilentina:6.059998,(Genista_demarcoi:4.039978,Genista_gasparrinii:4.039978,(Genista_tyrrhena&pontiana:2.019989,Genista_tyrrhena&pontiana2:2.019989,Genista_tyrrhena&pontiana3:2.019989,Genista_tyrrhena&tyrrhena:2.019989)N384:2.02)N383:2.02)N371:2.02,((Genista_michelii:2.693329,Genista_michelii2:2.693329)N386:2.693333,Genista_aristata:5.386658,Genista_cupanii:5.386658,Genista_madoniensis:5.386658)N385:2.693333,Retama_raetam&gussonei:8.079978)N370:2.02,Genista_sardoa:10.099976,(Genista_aquilana:6.733337,Genista_sericea&pollinensis:6.733337,(Genista_etnensis:3.366669,Genista_etnensis2:3.366669)N388:3.366667)N387:3.366667)N369:0.9)N365:46,(((Anthyllis_hermanniae&brutia:8,Anthyllis_hermanniae&corsica:8,Anthyllis_hermanniae&ichnusae:8,Anthyllis_hermanniae&sicula:8)N391:42.599998,((((Astragalus_parnassi&calabricus:8.266663,Astragalus_caprinus&huetii:8.266663,(Astragalus_nebrodensis:4.133331,Astragalus_siculus:4.133331)N396:4.133333,(Astragalus_genargenteus:4.133331,Astragalus_sirinicus:4.133331)N397:4.133333)N395:4.133333,(Astragalus_aquilanus:6.200012,Astragalus_muelleri&etruscus:6.200012)N398:6.2,((Astragalus_verrucosus:4.133331,Astragalus_maritimus:4.133331)N400:4.133333,Astragalus_raphaelis:8.266663)N399:4.133333)N394:1.525,Oxytropis_pilosa&caputoi:13.924987)N393:25.074999,(((Lathyrus_odoratus:24.32,(Vicia_sirinica:10.449524,(Vicia_giacominiana:2.612366,Vicia_tenuifolia&elegans:2.612366,Vicia_ochroleuca&ochroleuca:2.612366)N405:7.837143)N404:13.870476)N403:5.755001,(Trifolium_pratense&semipurpureum:23.727262,Trifolium_bivonae:23.72727)N406:6.347729)N402:1.075,Ononis_masquilleri:31.15)N401:7.849998)N392:11.599998)N390:3.700001,Bituminaria_morisiana:54.300003)N389:2.700001)N364:27.5,(Polygala_apiculata:27.266663,Polygala_carueliana:27.266663)N407:57.233334)N363:13.199997)N354:5.400002,(Elatine_gussonei:97.5,(((Euphorbia_corallioides:17.714294,(

Euphorbia_gasparrinii&samnitica:13.285706,Euphorbia_hyberna&insularis:13.285706,Euphorbia_papillaris:13.285706,(Euphorbia_ceratocarpa:8.857117,Euphorbia_ceratocarpa2:8.857117)N413:4.428572)N412:4.428571)N411:4.428572,(Euphorbia_pithyusa&cupanii:17.71426,((Euphorbia_meuseli:4.428589,Euphorbia_semiperfoliata:4.428589)N416:4.428571,Euphorbia_variabilis:8.857161)N415:8.857143)N414:4.428572)N410:47.357143,Mercurialis_corsica:69.5)N409:27.999996,(Hypericum_hircinum&hircinum:15,(Hypericum_scruglii:12.5,Hypericum_barbatum&calabricum:12.5)N418:2.5)N417:82.5,Linum_punctatum&punctatum:97.499985,(Salix_crataegifolia:89,((((Viola_aetnensis&aetnensis:2.397217,Viola_aetnensis&messanensis:2.397217,Viola_aetnensis&splendida:2.397217)N423:2.397222,Viola_bertolonii:4.794434,Viola_culminis:4.794434,Viola_etrusca:4.794434,Viola_merxmuelleri:4.794434,Viola_nebrodensis:4.794434,(Viola_pseudogracilis&cassinensis:2.397217,Viola_pseudogracilis&pseudogracilis2:2.397217)N424:2.397222,Viola_tineorum:4.794434,Viola_ucriana:4.794434)N422:2.397222,(Viola_corsica&ilvensis:3.595825,Viola_limbarae:3.595825,Viola_corsica&ilvensis2:3.595825)N425:3.595833,((Viola_eugeniae&eugeniae:2.397217,Viola_eugeniae&levieri:2.397217)N427:2.397222,Viola_ferrarinii:4.794434)N426:2.397222)N421:2.397223,(Viola_comollia:3.196289,Viola_magellensis:3.196289)N428:6.392592)N420:79.411118)N419:8.5)N408:5.599998)N353:5.900001)N293:7.73333,(((((Sedum_ochroleucum&mediterraneum:10,Sedum_ochroleucum&mediterraneum2:10)N433:10,Sempervivum_riccii:19.99999)N432:5,Sedum_magellense&magellense:25)N431:69.5,((Ribes_multiflorum&sandalioticum:44.399994,Ribes_sardoum:44.399994)N435:29.599998,((Saxifraga_hostii&rhaetica:6.090886,(Saxifraga_tombeanensis:4.872742,Saxifraga_vandellii:4.872742)N438:1.218182)N437:2.436364,(((Saxifraga_italica:1.421204,Saxifraga_italica2:1.421204)N441:1.421212,Saxifraga_arachnoidea:2.842416)N440:1.421212,(Saxifraga_exarata&ampullacea:1.421204,Saxifraga_pedemontana&cervicornis:1.421204)N442:2.842424)N439:4.263636)N436:65.472733)N434:20.5)N430:8.5,(Paeonia_officinalis&italica:97,(Paeonia_morisii:48.5,Paeonia_sandrae:48.5)N444:48.5)N443:6)N429:13.73333)N292:1.466669)N6:16.800009,(Berberis_vulgaris&aetnensis:97.299995,((Adonis_distorta:67.900009,((((Anemonoides_trifolia&brevidentata:4.597504,Anemonoides_trifolia&brevidentata2:4.597504)N451:21.392498,Callianthemum_kerneranum:25.98999)N450:20.609999,((Ranunculus_bilobus:16.825012,Ranunculus_bilobus2:16.825012,Ranunculus_magellensis:16.825012)N453:3.825,(Ranunculus_braunblanquetii:8.216675,Ranunculus_gortanii:8.216675,(Ranunculus_marsicus:4.108337,Ranunculus_marsicus2:4.108337,Ranunculus_marsicus3:4.108337,Ranunculus_marsicus4:4.108337)N455:4.108333,Ranunculus_silanus:8.216675)N454:12.433334,(((Ranunculus_apenninus:4,Ranunculus_pollinensis:4,Ranunculus_venetus:4)N458:1.216667,Ranunculus_spicatus&rupestris:5.216675,Ranunculus_monspeliacus&aspromontanus:5.216675,Ranunculus_monspeliacus&aspromontanus2:5.216675)N457:6.108334,(Ranunculus_angulatus:6.737488,Ranunculus_cordiger&diffusus:6.737488,Ranunculus_cymbalariifolius:6.737488,(Ranunculus_pratensis:1.612488,(Ranunculus_thomasii:0.537506,Ranunculus_thomasii2:0.537506)N461:1.075)N460:5.125)N459:4.5875)N456:9.325)N452:25.949999)N449:7.099998,((Helleborus_lividus&corsicus:8.339996,Helleborus_lividus&corsicus2:8.339996)N463:8.34,(Helleborus_viridis&bocconei:8.339996,Helleborus_viridis&bocconei2:8.339996,Helleborus_viridis&bocconei3:8.339996)N464:8.34)N462:37.019997)N448:14.200004)N447:7.1,((Aquilegia_lucensis:2.540009,Aquilegia_ophiolitica:2.540009,(Aquilegia_bertolonii:1.27002,Aquilegia_champagnatii:1.27002,Aquilegia_marcelliana:1.27002,Aquilegia_magellensis:1.27002)N467:1.27,(Aquilegia_barbaricina:1.27002,Aquilegia_nugorensis:1.27002,Aquilegia_nuragica:1.27002)N468:1.27,Aquilegia_sicula:2.54002)N466:25.029999,Thalictrum_calabricum:27.57)N465:47.43)N446:22.300003)N445:37.699997)N5:12.800003,(((((((((Acis_rosea:2.799988,Acis_rosea2:2.799988)N477:5.6,((Narcissus_supramontanus&cunicularium:5.333344,Narcissus_supramontanus&supramontanus:5.333344)N479:2.666667,Pancratium_illyricum:8,Pancratium_illyricum2:8)N478:0.4)N476:51.599998,((Allium_insubricum:27,Allium_vernale:27)N481:30.5,(((Allium_parciflorum:4.200012,Allium_parciflorum2:4.200012,Allium_parciflorum3:4.200012,Allium_hemisphaericum:4.199988)N484:22.799999,(Allium_nebrodense:20.25,Allium_garganicum:20.25,Allium_calabrum:20.25,Allium_anzalonei:20.25,(Allium_aetnense:13.5,Allium_agrigentinum:13.5,Allium_apulum:13.5,Allium_diomedeum:13.5,Allium_garbarii:13.5,Allium_lehmannii:13.5,Allium_lopadusanum:13.5,(Allium_castellanense:6.75,Allium_castellanense2:6.75)N487:6.75)N486:6.75)N485:6.75)N483:6,(Allium_franciniae:22,(Allium_obtusiflorum:11,Allium_obtusiflorum2:11)N489:11)N488:11,(Allium_cupanii:15,Allium_panormitanum:15,Allium_pelagicum:15,Allium_pentadactyli:15,Allium_samniticum:15)N490:18)N482:24.5)N480:2.5)N475:20.666664,((((Bellevalia_webbiana:6.857117,(Bellevalia_dubia:3.428571,Bellevalia_pelagica:3.428571)N495:3.428571)N494:3.428571,(Muscari_botryoides&longifolium:7.714282,Muscari_gussonei:7.71429)N496:2.571428)N493:2.571428,((Oncostema_dimartinoi:6.428589,Oncostema_ughii:6.428589,(Oncostem

a_sicula:3.214294,Oncostema_sicula2:3.214294)N499:3.214286)N498:3.214285,(Prospero_corsicum:4.821411,Prospero_hierae:4.821411)N500:4.821428)N497:3.214286)N492:12.142859,Charybdis_glaucophylla:25,(((Ornithogalum_corsicum:5.555555,Ornithogalum_corsicum2:5.555542,Ornithogalum_corsicum3:5.555542)N503:5.555555,(Ornithogalum_exscapum:5.555555,Ornithogalum_exscapum2:5.555542)N504:5.555555)N502:5.555555,(((Ornithogalum_etruscum&etruscum:4.166656,Ornithogalum_etruscum&etruscum2:4.166656,Ornithogalum_etruscum&etruscum3:4.166656)N507:4.166667,(Ornithogalum_etruscum&umbratile:4.166656,Ornithogalum_etruscum&umbratile2:4.166656,Ornithogalum_etruscum&umbratile3:4.166656)N508:4.166667)N506:4.166667,Ornithogalum_orthophyllum&orthophyllum:12.5)N505:4.166666)N501:8.333335)N491:55.666664)N474:20.333332,(((((Crocus_minimus:4.399994,Crocus_minimus2:4.399994,Crocus_minimus3:4.399994,Crocus_minimus4:4.399994,Crocus_minimus5:4.399994,Crocus_minimus6:4.399994,Crocus_minimus7:4.399994,Crocus_minimus8:4.399994,Crocus_minimus9:4.399994)N513:4.4,Crocus_imperati:8.799988,Crocus_suaveolens:8.799988)N512:4.4,(Crocus_siculus:6.599976,(Crocus_etruscus:3.299988,Crocus_ilvensis:3.299988)N515:3.3)N514:6.6,(Crocus_biflorus:6.6,Crocus_biflorus2:6.599976)N516:6.6)N511:3.3,(Romulea_linaresii&linaresii:4.125,Romulea_linaresii&linaresii2:4.125)N517:12.375)N510:47.5,(Iris_pseudopumila:30,Iris_pseudopumila2:30,Iris_pseudopumila3:30,Iris_pseudopumila4:30,Iris_pseudopumila5:30,(Iris_bicapitata:20,Iris_calabra:20,Iris_marsica:20,Iris_revoluta:20,Iris_setina:20,Iris_cengialti&cengialti:20,(Iris_relicta:10,Iris_relicta2:10)N520:10)N519:10)N518:34,(Romulea_bocchierii:32,Romulea_revelieri:32,Romulea_variicolor:32)N521:32,(Romulea_insularis:32,Romulea_requienii:32)N522:32)N509:37)N473:17,(((Epipactis_cupaniana:10.799988,(Epipactis_helleborine&aspromontana:5.399994,Epipactis_helleborine&schubertiorum:5.399994)N526:5.4)N525:32.200001,Limodorum_brulloi:43)N524:21,(Nigritella_buschmanniae:18,((((Ophrys_bertolonii&bertoloniiformis:3.375,Ophrys_bertolonii&bertoloniiformis2:3.375)N531:3.375,(Ophrys_parvimaculata2:4.5,Ophrys_peucetiae:4.5,(Ophrys_apulica:2.25,Ophrys_apulica2:2.25)N533:2.25,(Ophrys_chestermanii:2.25,Ophrys_chestermanii2:2.25)N534:2.25,(Ophrys_oxyrrhynchos&celiensis:2.25,Ophrys_oxyrrhynchos&oxyrrhynchos:2.25)N535:2.25)N532:2.25,Ophrys_pseudomelena:6.75,Ophrys_pseudomelena2:6.75,(Ophrys_biscutella:2.25,Ophrys_classica:2.25,Ophrys_crabonifera:2.25,Ophrys_crabonifera3:2.25,Ophrys_exaltata&morisii:2.25,Ophrys_lunulata:2.25,Ophrys_pollinensis:2.25,Ophrys_pollinensis2:2.25,Ophrys_promontorii:2.25,Ophrys_promontorii2:2.25,Ophrys_promontorii3:2.25,Ophrys_promontorii4:2.25,Ophrys_promontorii5:2.25,Ophrys_promontorii6:2.25,Ophrys_sipontensis:2.25,Ophrys_sipontensis2:2.25,Ophrys_tarentina:2.25)N536:4.5)N530:2.25,Ophrys_tardands:9)N529:2.25,((Orchis_brancifortii:2.25,Orchis_mascula&ichnusae:2.25)N538:6.75,(Serapias_intermedia&hyblaea:4.5,Serapias_orientalis&apulica:4.5,Serapias_orientalis&siciliensis:4.5)N539:4.5)N537:2.25)N528:6.75)N527:46)N523:54)N472:4,(((Carex_ferruginea:12.1,Carex_microcarpa:12.100006)N542:61.424995,(Luzula_calabra:37.125,Luzula_spicata&spicata:37.125)N543:36.399994)N541:7.475007,((((((((Agrostis_canina&montelucii:1.666656,Agrostis_canina&montelucii2:1.666656,Agrostis_canina&montelucii3:1.666656)N551:6.666668,Calamagrostis_corsica:8.333354)N550:1.666667,Phalaroides_arundinacea&rotgesii:10.00002)N549:7.200001,(((Drymochloa_drymeia&exaltata:12.899988,((((Festuca_alfrediana&alfrediana:2.100006,Festuca_alfrediana&ferrariniana:2.100006)N558:2.1,((Festuca_robustifolia:1.399994,Festuca_robustifolia2:1.399994)N560:1.4,Festuca_humifusa:2.799994,(Festuca_gamisansii&aethaliae:1.399994,Festuca_apuanica:1.399994)N561:1.4,Festuca_riccerii:2.799988,Festuca_veneris:2.799988)N559:1.4)N557:3.75,(Festuca_violacea&italica:2.700012,Festuca_violacea&italica2:2.700012,Festuca_violacea&puccinellii:2.700012,Festuca_violacea&puccinellii2:2.700012)N562:5.25)N556:1.650001,Festuca_sardoa:9.600012)N555:3.3)N554:0.9,((Sesleria_italica:5.52002,Sesleria_nitida:5.52002,(Sesleria_pichiana:2.76001,Sesleria_pichiana2:2.76001)N565:2.76)N564:2.76,Sesleria_calabrica:8.280014,(Sesleria_barbaricina:4.140015,Sesleria_insularis:4.140015)N566:4.14)N563:5.52)N553:1.7,Phleum_sardoum:15.499975)N552:1.7,Trisetaria_gracilis:17.200012,Trisetaria_villosum:17.200012)N548:7.8,Elytrigia_corsica:24.999994)N547:3,(Brachypodium_genuense:18.666687,Brachypodium_genuense2:18.666687)N567:9.333333)N546:9.5,Stipa_dasyvaginata&apenninicola:37.500011)N545:10,Bellardiochloa_variegata&aetnensis:47.5)N544:33.5)N540:41)N471:1,((Colchicum_arenasii:4.666656,(Colchicum_gonarei:2.333344,Colchicum_gonarei3:2.333344)N570:2.333333,(Colchicum_gracile:2.333344,Colchicum_gracile2:2.333344)N571:2.333333,(Colchicum_neapolitanum:2.333344,Colchicum_neapolitanum2:2.333344,Colchicum_neapolitanum3:2.333344,Colchicum_neapolitanum4:2.333344,Colchicum_neapolitanum5:2.333344,Colchicum_neapolitanum6:2.333344,Colchicum_neapolitanum7:2.333344)N572:2.333333)N569:105.333336,((Gagea_tisoniana:5.119049,Gagea_tisoniana2:5.119049)N574:10.238095,(Gagea_sicula:6.142853,Gagea_peruzzii:6.142853,(Gagea_chrysantha:3.071411,Gagea_chrysantha2:3.071411)N576:3.0714

29)N575:9.214285)N573:94.64286)N568:13)N470:8,(Arum_apulum:12.5,Arum_apulum2:12.5)N577:118.5)N469:16.800003)N4:2.300003,(Aristolochia_clusii:72,(Aristolochia_sicula:36,Aristolochia_tyrrhena:36)N579:36)N578:78.100006)N3:204.899994,Pinus_laricio:355)N2:70.500031,(Isoetes_malinverniana:309,(Isoetes_todaroana:154.5,Isoetes_todaroana2:154.5)N581:154.5)N580:116.500031)N1;
